# Transcriptome-Wide Analysis of Hepatitis B Virus-Mediated Changes to Normal Hepatocyte Gene Expression

**DOI:** 10.1371/journal.ppat.1005438

**Published:** 2016-02-18

**Authors:** Jason Lamontagne, Joshua C. Mell, Michael J. Bouchard

**Affiliations:** 1 Graduate Program in Microbiology and Immunology, Graduate School of Biomedical Sciences and Professional Studies, Drexel University College of Medicine, Philadelphia, Pennsylvania, United States of America; 2 Department of Microbiology and Immunology, Center for Genomic Sciences, Drexel University College of Medicine, Philadelphia, Pennsylvania, United States of America; 3 Department of Biochemistry and Molecular Biology, Drexel University College of Medicine, Philadelphia, Pennsylvania, United States of America; University of California, San Diego, UNITED STATES

## Abstract

Globally, a chronic hepatitis B virus (HBV) infection remains the leading cause of primary liver cancer. The mechanisms leading to the development of HBV-associated liver cancer remain incompletely understood. In part, this is because studies have been limited by the lack of effective model systems that are both readily available and mimic the cellular environment of a normal hepatocyte. Additionally, many studies have focused on single, specific factors or pathways that may be affected by HBV, without addressing cell physiology as a whole. Here, we apply RNA-seq technology to investigate transcriptome-wide, HBV-mediated changes in gene expression to identify single factors and pathways as well as networks of genes and pathways that are affected in the context of HBV replication. Importantly, these studies were conducted in an *ex vivo* model of cultured primary hepatocytes, allowing for the transcriptomic characterization of this model system and an investigation of early HBV-mediated effects in a biologically relevant context. We analyzed differential gene expression within the context of time-mediated gene-expression changes and show that in the context of HBV replication a number of genes and cellular pathways are altered, including those associated with metabolism, cell cycle regulation, and lipid biosynthesis. Multiple analysis pipelines, as well as qRT-PCR and an independent, replicate RNA-seq analysis, were used to identify and confirm differentially expressed genes. HBV-mediated alterations to the transcriptome that we identified likely represent early changes to hepatocytes following an HBV infection, suggesting potential targets for early therapeutic intervention. Overall, these studies have produced a valuable resource that can be used to expand our understanding of the complex network of host-virus interactions and the impact of HBV-mediated changes to normal hepatocyte physiology on viral replication.

## Author Summary

Chronic infection with the hepatitis B virus (HBV) is the leading global cause of primary liver cancer; however, therapeutics for the treatment of chronic HBV are limited in both scope and efficacy. Infection with HBV results in an incompletely understood, complex network of host-virus interactions. To attempt to better understand these interactions, we assessed HBV-mediated changes to normal hepatocyte gene expression on a transcriptome-wide scale. By identifying gene expression that is altered by HBV, we were able to demonstrate that HBV affects multiple cellular signaling pathways that previously have been associated with carcinogenesis. As most HBV-related studies have investigated either late-stage changes in hepatocyte physiology or looked at cellular changes on a more narrow scale, our results represent an important advancement towards identifying early events associated with HBV replication, upstream of the development of HBV-associated disease. Additionally, our studies allowed us to characterize transcriptome changes that occur in a primary hepatocyte culture model, an important advancement in the confirmation of this commonly used model system as a biologically relevant alternative to transformed cell lines.

## Introduction

Despite the availability of an effective vaccine, hepatitis B virus (HBV) infection remains a significant health concern with ~350 million people chronically infected worldwide [1]. Approximately 25% of these chronically infected individuals will go on to develop HBV-associated hepatocellular carcinoma (HCC), the most common primary liver cancer, making chronic infection with HBV the leading risk factor for the development of HCC [1–3]. Globally, liver cancer is the second leading cause of cancer-related death, with nearly 750,000 deaths annually and an incidence to mortality ratio near 1 [4]. Current treatment options for HBV-infected patients are limited to a small number of approved therapies, including reverse transcriptase inhibitors and interferon. Each of these treatments has its potential drawbacks, including side effects of treatment and the development of escape mutants, and no therapy has been developed that reaches the level of complete cure [5]. A better understanding of HBV-mediated cellular changes in the context of viral replication is needed to expand our knowledge of virus-dependent factors and pathways, ultimately leading to the identification of novel therapeutic targets.

Hepatocytes are the main target of an HBV infection, and numerous studies have been done to examine the impact of HBV replication on hepatocyte physiology. Like many viruses, HBV hijacks and manipulates cellular pathways to optimize conditions for viral replication and increase long-term survival of the virus. For example, because only 1 in 20,000 hepatocytes in the liver is actively dividing at any given time [6], HBV causes infected hepatocytes to exit G_0_ and enter the active cell cycle to stimulate viral replication [7, 8]. Previous studies have shown that HBV replication is cell-cycle dependent and that HBV modulates levels or activation of various cell-cycle regulators, including, CDK1, Cyclin D, Cyclin E, p21, and CDK2 [7, 9–16] (and reviewed in [17, 18]). In addition to cell-cycle regulation, many other cellular signal transduction pathways are altered during an HBV infection, including those related to metabolism, such as the PI3K/AKT pathway [19, 20], the mTOR pathway [21–24], and the MAPK pathway [13, 25, 26]. The results of some studies also suggest that the miRNA profile of hepatocytes changes in response to HBV replication (reviewed in [27–29]).

One caveat to many HBV-related studies is that they have often focused on specific factors or specific cellular signal transduction pathways to determine how these affect HBV replication. This targeted approach has precluded an unbiased understanding of the impact of HBV on the cell as a whole. Historically, these targeted interrogations of HBV effects have been necessary due to the limitations of technology and the lack of high-throughput approaches to examine cellular changes on a system-wide scale. The establishment of RNA-seq now offers a method to generate a complete expression profile of the cellular transcriptome and analyze treatment-mediated changes to gene expression on a transcriptome-wide scale. Because HBV can affect many different cellular signal transduction factors, pathways, and functions, the application of global approaches like RNA-seq to HBV-expressing cells should help establish a comprehensive picture of the cellular pathways altered simultaneously during HBV replication and how these pathways are interconnected.

Recently, several groups have utilized RNA-seq to analyze the transcriptome of the normal liver and to define gene-expression changes in the context of liver cancer or disease, including HBV infection [30–37]. While supplying valuable information, most of these studies have not examined the early cellular impact of HBV replication. Instead, they assessed chronic HBV infection or HBV-associated HCC through the use of chronically infected patient samples or cell lines stably expressing the HBV genome. Therefore, it is difficult to determine if the reported changes in gene expression reflect causes or consequences of advanced liver disease and how these changes relate to HBV replication. One example of a study that did directly analyze early changes to the transcriptome in the context of HBV replication, and not downstream disease, is a study that utilized the Huh7 human hepatoma cell line transfected with a plasmid expressing the HBV genome. This study used RNA-seq to investigate an early time point in HBV-mediated alteration of multiple cellular pathways and processes, including those related to the metabolic state of the cell and the innate immune response. However, potentially due to the use of a transformed cell line, some cellular signaling pathways previously reported as altered by HBV, such as cell-cycle regulation, were not altered in this system [34]. In addition, two related transcriptome-based studies utilized the woodchuck hepatitis virus (WHV) as a model to identify *in vivo* virus-mediated changes to the liver transcriptome in the context of either chronic or acute disease; WHV infection of woodchucks has served as a model system for understanding disease progression associated with the highly related human HBV. These WHV studies offered interesting insights into the immune response in both a chronic and self-resolving WHV infection, including characterization of WHV (and HBV by extension) as a stealth virus that avoids immune detection and induces only minimal gene-expression changes during the early stages of infection. Overall, the importance of virus-mediated changes to the hepatocyte transcriptome was demonstrated by showing that, based on the similarity of the transcriptional profile of infected animals, the hepatic transcriptome was enough to cluster animals into those which developed chronic disease or those which experienced an acute, self-resolving infection. Importantly, these studies focused on the transcriptome changes across the entire liver, and were centered on changes related to the immune response in an attempt to identify factors that could contribute to the development of a chronic disease instead of a self-limiting, acute infection [30, 38].

The narrow host range and cellular tropism of HBV have made it difficult to find a tractable model system that can be used to study the impact of HBV replication and HBV protein expression on normal hepatocyte physiology. This has led to a reliance on various liver cancer-derived cell lines, such as the Huh7 hepatoma cell line [39] and the HepG2 hepatoblastoma cell line [40]. Extended passages in culture of these cells have likely allowed them to adapt to an environment that is significantly different from the *in vivo* liver environment. In addition, these cell lines and most other commonly used liver cell lines are tumor-derived, and their cellular physiology will likely be more similar to that seen in a tumor than a normal hepatocyte. In fact, the results of a recent transcriptome study led to the conclusion that, because of the significant difference between HepG2 cells and primary liver tissue, researchers should be wary of using HepG2 cells as a model for gene expression of normal hepatocytes [37]. Similar differences from primary hepatocytes have also been observed in other immortalized hepatocyte cell lines [41]. Therefore, when using established cell lines as a model for HBV studies, it is important to consider their deviation from a normal hepatocyte because their already altered baseline of protein and RNA expression means that it might be difficult to determine if changes seen in these systems are an HBV-mediated effect that would be observed in normal hepatocytes.

Here, we report a comprehensive transcriptome-wide analysis of HBV-mediated changes in hepatocyte gene expression. Using RNA-seq, we generated a profile of HBV-mediated changes to the transcriptome of primary rat hepatocytes (PRHs) expressing HBV. We have previously shown that PRHs can serve as a surrogate model for HBV-mediated effects seen in primary human hepatocytes, suggesting that PRHs are a biologically relevant model system [8, 20]. Our experimental design also allowed us to characterize the systems being used, expanding the implications of our findings beyond HBV-specific effects. For example, we characterized changes in primary hepatocyte gene expression that are due to isolation and culture and confirmed the utility of our *ex vivo* PRH model as a biologically relevant system for liver studies. We also quantified adenoviral transcript expression and adenovirus-mediated effects on hepatocyte-gene expression that were associated with the recombinant adenoviruses used in our system and confirmed that the use of recombinant adenoviruses is an effective delivery system that has minimal cellular impact. Together, our findings will help to guide future research into the cellular mechanisms which both affect, and are affected by HBV replication, and could generate a better understanding of the molecular mechanisms associated with HBV-associated pathogenesis and lead to the identification of novel therapeutic targets for preventing the development of HBV-associated disease.

## Results

### Adenovirus infection of cultured primary rat hepatocytes

Because of the narrow host range of HBV, PRHs cannot be directly infected with HBV. Most systems that mimic a natural infection, including cultured primary human hepatocytes, have proven to be limited by low efficiency of infection or sample-to-sample variability [42–45]. Alternatively, recombinant adenovirus can be used as a surrogate vehicle for the delivery of the HBV genome, allowing studies of steps of the HBV life cycle post-entry, including replication and viral protein expression [20, 46, 47]. Contrary to infection with purified HBV, using recombinant adenovirus allows a very high infection efficiency of cultured primary hepatocytes. Similarly, cultured PRHs have served as an effective model for studying the cellular effects of the post-infection stages of the HBV life cycle, and effects seen in PRHs correlate well with HBV-mediated effects seen in cultured primary human hepatocytes [8, 20]. This allows us to confidently bypass many of the drawbacks of using cultured primary human hepatocytes, including their intermittent availability and high cost, and the unknowns associated with patient-to-patient variations and the fact that these hepatocytes are often isolated from resected samples of livers from patients with an underlying liver disease. Using a recombinant adenovirus that expresses a greater-than-unit length copy of the HBV genome, we routinely achieve a high infection efficiency (>75%) in cultured PRHs, and have previously identified HBV-mediated cellular changes and the presence of HBV replication as early as 24hr post-infection [48]. Although this experimental system only allows the study of transient HBV-mediated changes, we have been able to utilize it to better understand the network of hepatocyte-virus interactions during HBV replication.

**Fig 1:**
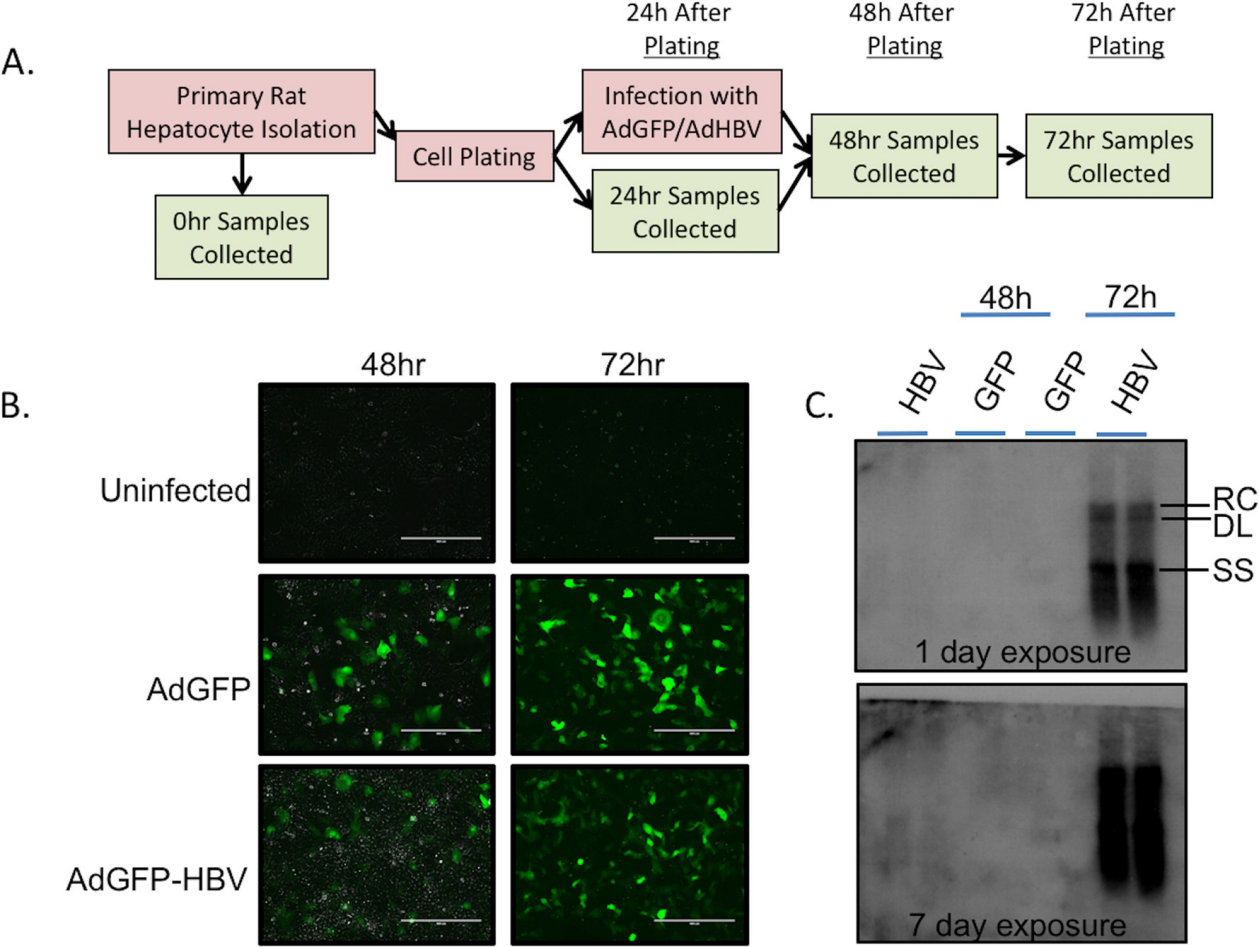
Confirmation of experimental system. **A**. Experimental setup for primary dataset. **B**. PRHs were infected with either AdGFP or AdGFP-HBV, and infection efficiency was determined by monitoring GFP expression at 48hr and 72hr (24hr and 48hr post-infection). **C.** HBV replication was monitored by Southern blot analysis of HBV core particle-associated DNA. Blot was exposed for 1 day (upper panel) or 7 days (lower panel) to allow visualization of HBV replication at both 48hr and 72hr. RC–relaxed circular DNA, DL–double-stranded linear DNA, SS–single stranded DNA.

For studies described here, uninfected PRHs were collected before plating (0hr), and at 24hr, 48hr, and 72hr after plating. In addition, PRHs were infected at 24hr after plating with either an adenoviral vector expressing hrGFP alone (AdGFP), or expressing hrGFP and a greater-than-unit length copy of the HBV genome (AdGFP-HBV). Infected PRHs were collected at 48hr (24hr after infection) and 72hr (48hr after infection) (Fig 1A). Infection of PRHs with either AdGFP or AdGFP-HBV resulted in an ~85% infection efficiency, as determined by GFP expression (Fig 1B). This level of infection was utilized to ensure that individual cells were not overloaded with virus, but that the large majority of hepatocytes had replicating HBV, similar to what is seen *in vivo* where nearly all hepatocytes of the liver have replicating HBV relatively soon after infection [49, 50]. HBV replication was measured by Southern blot for HBV core-particle-associated DNA. While HBV replication intermediates were clearly visible at 72hr after plating (48hr after infection) in AdGFP-HBV infected cells (Fig 1C, upper panel), a low level of replication at 48hr (24hr after infection) was also visible when the film was exposed for a longer time (Fig 1C, bottom panel), confirming the presence of replicating HBV in our system. Together with previously published reports [7, 20, 48], these results support the use of PRHs infected with HBV-expressing adenovirus as an effective model for studying the cellular impact of HBV replication.

### Analysis of RNA-seq data

A simplified workflow of the complete RNA-seq analysis is shown in S1A Fig. While multiple tools were used for independent cross-validation at each step, the analysis reported here was done with a pipeline consisting of: STAR aligner > Sambamba filter for unique reads > GenomicAlignments (within R) > DESeq2 (within R) > iPathwayGuide. A comparison of the output of differential expression analyses from multiple pipelines, including substituting BWA for STAR, edgeR for DESeq2, or use of the complete Tuxedo pipeline, can be found in the supporting information (S1B and S1C Fig and S1 Table).

**Fig 2:**
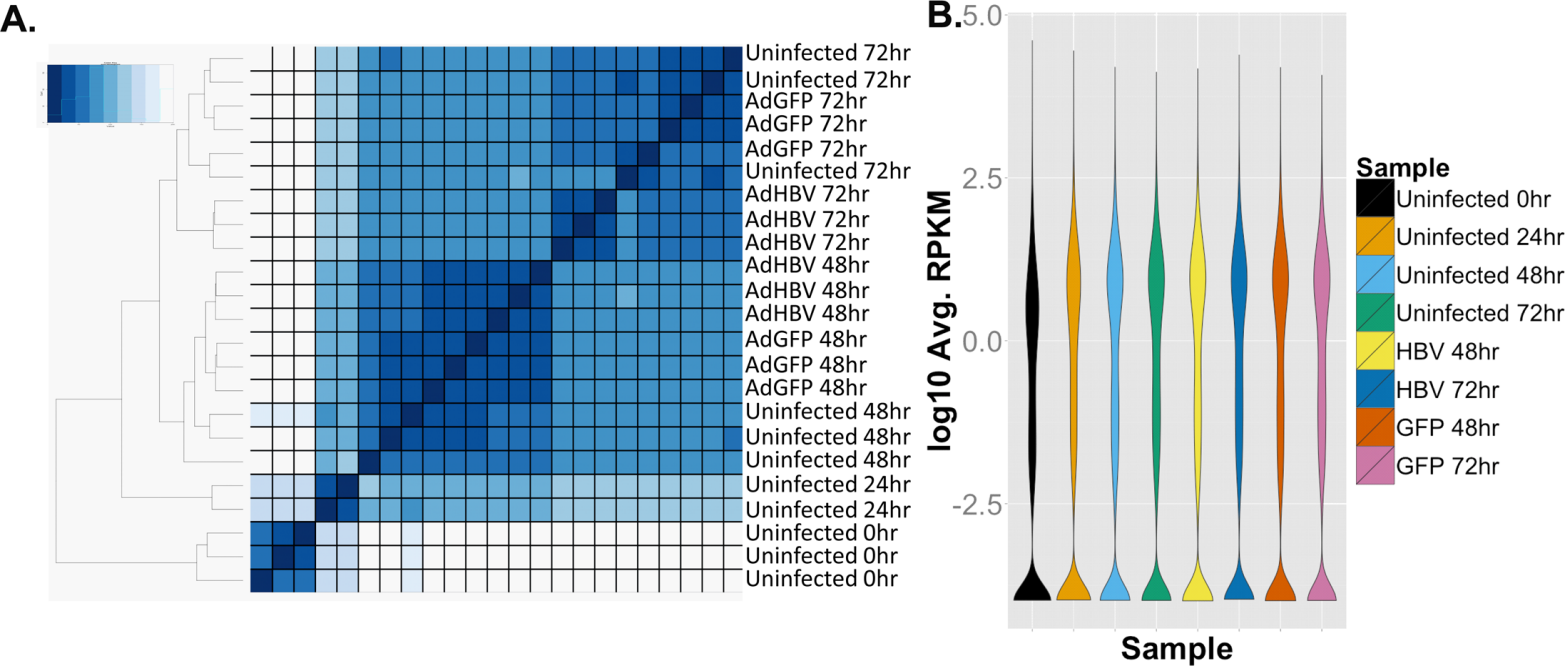
RNA-seq overview. **A.** Euclidean sample distance was mapped allowing unbiased ordering of samples based on sample similarity. **B.** Plot of distribution of average RPKM values per sample.

Sequencing of the primary dataset resulted in ~35–47 million reads per sample, for a total of ~950 million reads across 23 samples (Table 1). Importantly, all samples had between 89% and 95% of total reads mapping, with a high percentage of uniquely mapping reads (~80–86%). Investigation of the Euclidean distance between all samples showed that time point was the principal variable, with samples grouped by replicate and treatment within each time point (Fig 2A). Overall, a total of 20,700 transcripts (78% of features in the gene annotation) were identified in the RNA-seq analysis as having at least one read aligned, and 12,343 genes had at least 1 aligned read in all samples. After normalization of the data by calculating reads per kilobase of transcript per megabase library size (RPKM), 8,033 genes had an RPKM value of at least 1 in each sample. The mean RPKM value across all samples was 16.23, and the mean RPKM was 49.42 for genes with an RPKM ≥1. Additionally, the distribution of average RPKM per transcript was uniform between the eight sample groups (Fig 2B). These results indicate a strong degree of coverage, a general uniformity in transcriptome composition, and a lack of global shifts in transcript levels across samples. Therefore, it is unlikely that any differences seen in subsequent analysis are the result of sequencing or sample bias, and are instead the result of time point or treatment-mediated effects.

**Table 1:** RNA-seq overview.

Sample	Total Reads	Mapped	% Mapped	Uniquely Mapped	% Unique Mapped	% Unmapped	Multi-mapping Reads	% Multiple Mapping	Total Mappings of Reads
Uninf 0hr 1	37.03	34.24	92.48%	30.89	83.43%	7.52%	3.35	9.05%	40.53
Uninf 0hr 2	37.66	35.55	94.38%	32.21	85.52%	5.62%	3.34	8.86%	41.55
Uninf 0hr 3	36.43	33.95	93.20%	30.71	84.31%	6.80%	3.24	8.89%	39.74
Uninf 24hr 1	34.64	31.94	92.22%	28.02	80.90%	7.78%	3.92	11.31%	39.40
Uninf 24hr 2	45.74	43.03	94.07%	37.84	82.73%	5.93%	5.19	11.34%	53.01
Uninf 48hr 1	39.69	37.03	93.29%	32.58	82.08%	6.71%	4.45	11.21%	45.66
Uninf 48hr 2	40.57	36.70	90.46%	32.40	79.87%	9.54%	4.30	10.59%	45.10
Uninf 48hr 3	43.95	41.01	93.32%	35.82	81.51%	6.68%	5.19	11.81%	52.14
AdHBV 48hr 1	44.40	41.78	94.08%	36.85	82.99%	5.92%	4.93	11.09%	51.16
AdHBV 48hr 2	44.44	41.21	92.73%	36.42	81.96%	7.27%	4.79	10.77%	50.24
AdHBV 48hr 3	41.48	38.16	91.99%	33.68	81.19%	8.01%	4.48	10.80%	46.89
AdGFP 48hr 1	45.56	42.86	94.08%	37.80	82.97%	5.92%	5.06	11.12%	52.36
AdGFP 48hr 2	46.73	43.77	93.67%	38.65	82.72%	6.33%	5.12	10.95%	53.19
AdGFP 48hr 3	44.49	41.53	93.35%	36.60	82.27%	6.65%	4.93	11.08%	50.75
Uninf 72hr 1	44.59	40.28	90.33%	35.55	79.73%	9.67%	4.73	10.60%	50.92
Uninf 72hr 2	36.99	34.23	92.55%	30.53	82.55%	7.45%	3.70	10.00%	41.36
Uninf 72hr 3	40.47	38.08	94.10%	34.07	84.18%	5.90%	4.02	9.92%	45.30
AdHBV 72hr 1	36.58	33.72	92.17%	30.09	82.24%	7.83%	3.63	9.93%	40.48
AdHBV 72hr 2	40.48	36.99	91.38%	33.08	81.71%	8.62%	3.91	9.67%	44.23
AdHBV 72hr 3	40.09	37.41	93.31%	33.45	83.44%	6.69%	3.96	9.87%	44.64
AdGFP 72hr 1	42.45	39.69	93.50%	35.47	83.55%	6.50%	4.23	9.95%	47.52
AdGFP 72hr 2	39.18	36.44	93.00%	32.55	83.08%	7.00%	3.89	9.92%	43.52
AdGFP 72hr 3	37.67	33.60	89.20%	29.66	78.74%	10.80%	3.94	10.45%	42.21

*Values represent millions of reads

### HBV-mapping reads

HBV has a unique and highly compact genomic organization that results in every base being used within at least one open reading frame. As part of this compact organization, all of the viral transcripts share the same polyadenylation (polyA) signal, creating a series of overlapping transcripts with each longer transcript a 5' sequence extension of each of the shorter transcripts [51]. For example, as the shortest transcript, the transcript encoding the HBV X protein (HBx) is also included in the 3' end of all other HBV transcripts. It would be expected, therefore, that since multiple transcripts contain the HBx sequence, a higher number of reads in this RNA-seq analysis should be generated from the HBx region of the HBV genome than from other regions of the genome. Similarly, we expect to see an incremental decrease in coverage as we move away from the polyA signal toward the 5' end of the transcripts, and as the number of viral transcripts incorporating each base decreases.

**Fig 3:**
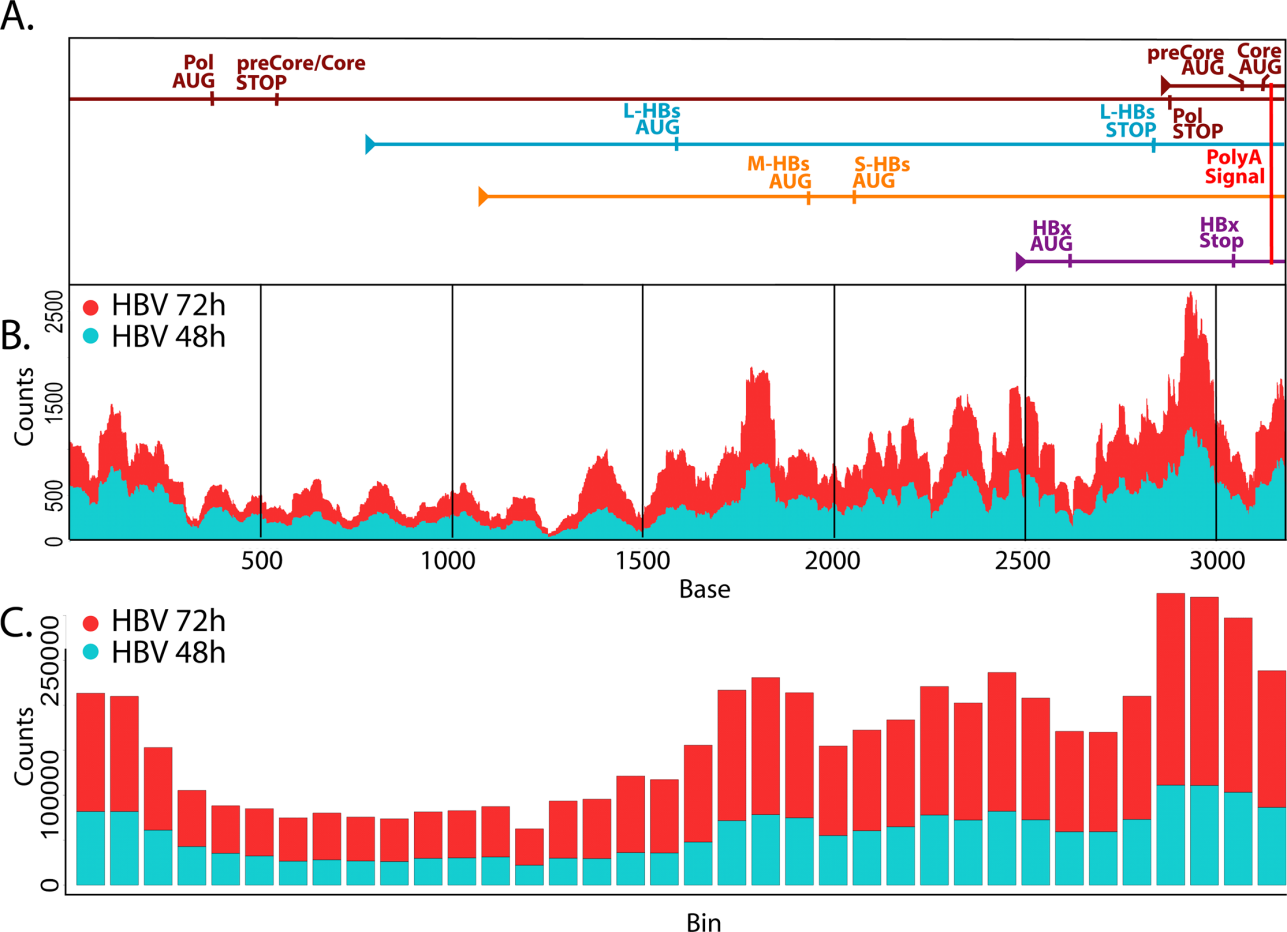
Mapping HBV-derived reads. **A.** HBV transcripts were aligned based on the location of the shared polyA signal plus an additional 10 bases. Genomic transcripts are greater-than-genome length, with the terminally redundant portion apparent as overlap within the same transcript. **B.** Average reads-per-base (normalized to library size) from AdGFP-HBV 48hr (blue) and 72hr (red) samples were plotted along the HBV genome/transcripts. **C.** The HBV genome was broken down into 172 base sliding bins with 50% overlap between bins. Average number of reads-per-bin for AdGFP-HBV-infected PRHs at 48hr (blue) and 72hr (red) were plotted.

To test this, we reordered the HBV genomic sequence such that the TATAAA polyA signal plus 10bp [52] were situated at the 3' end of the sequence, and we lined up the transcripts with each aligned by the 3' end to show the overlapping nature of the transcripts (Fig 3A). We then plotted depth-per-base across the HBV sequence and identified a distinct pattern of expression that was reproduced in AdGFP-HBV-infected PRHs at both time points (48hr and 72hr) in both the primary (Fig 3B) and secondary datasets (secondary dataset described below). To account for variation in coverage due to sequencing bias, the genome (organized as described above) was broken down into overlapping bins (172bp bins with 86bp overlap), and total reads were counted within each bin (Fig 3C). Apart from a slightly higher level of coverage at the beginning of the sequence, an obvious pattern of increased base usage was seen from 5' to 3' approaching the polyA signal, as expected. It is possible that higher coverage at the beginning of the sequence could be due to variable lengths of the transcripts after the polyA signal, which would wrap around to the earlier bases on the figure; however, additional studies would be needed to address this hypothesis. While these results do not allow us to determine the kinetics of expression for the individual HBV transcripts, they do support the overall hypothesis of overlapping transcripts with bases closer to the polyA signal being present in more transcripts.

### Adenovirus-mapping reads

**Fig 4:**
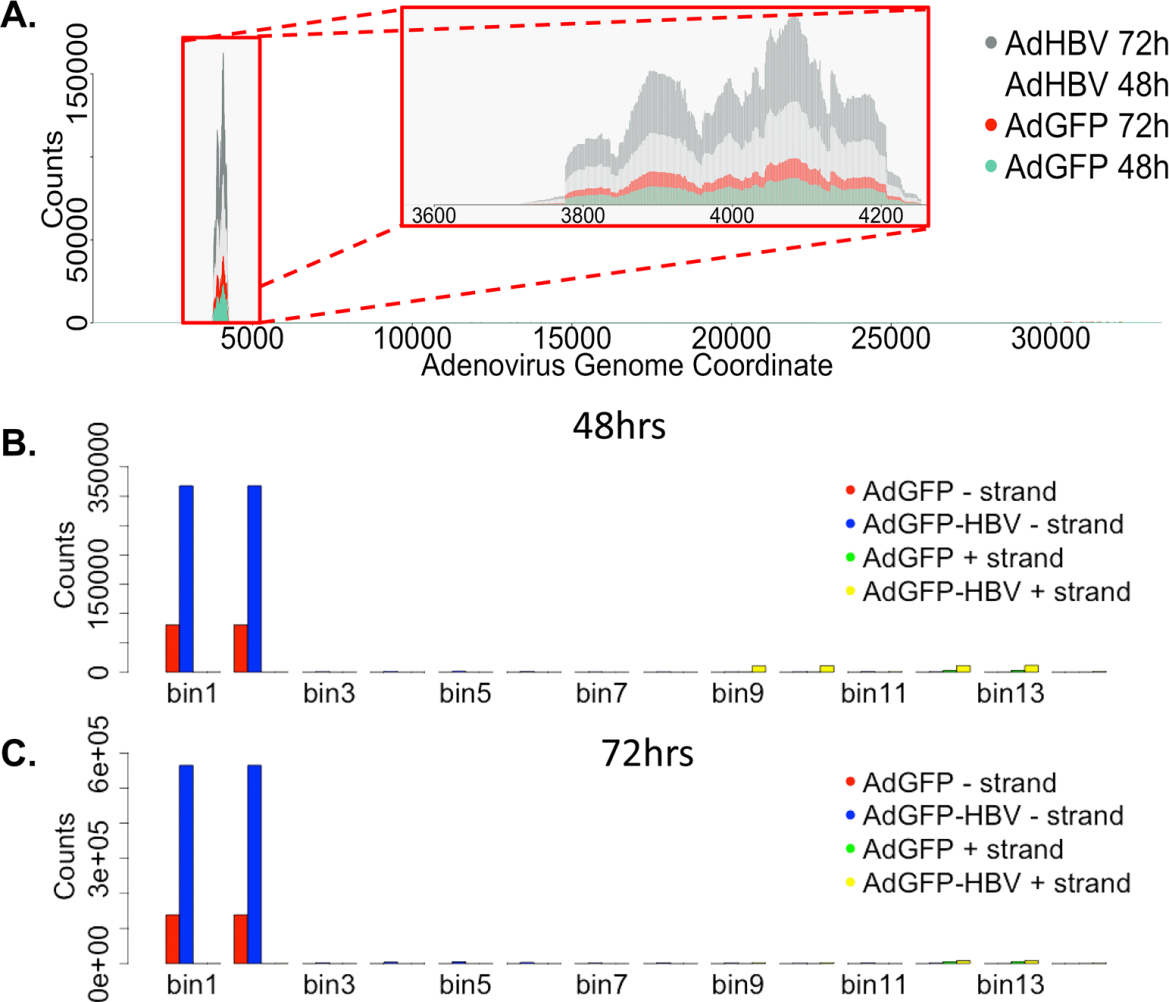
Mapping adenovirus-derived reads. **A.** Average reads per base (normalized to library size) from AdGFP-HBV 48hr (light grey), AdGFP-HBV 72hr (dark grey), AdGFP 48hr (green), and AdGFP 72hr (red) was plotted along the sequence of the pAdEasy-1 vector. **B.** The pAdEasy-1 sequence was broken down into 5kb sliding bins with 50% overlap between bins. Average number of reads per bin (for both the + and—strand) was plotted for 48hr samples. AdGFP—strand (red), AdGFP-HBV-strand (blue), AdGFP + strand (green), AdGFP-HBV—strand (yellow). **C.** Reads per bin were plotted for 72hr samples as in B.

Because we used recombinant adenovirus as the delivery vehicle for the HBV genome, we investigated the potential expression of adenoviral genes in our system. Although reads that mapped to the pAdEasy-1 sequence were detected in all infected samples, plotting reads-per-base across the genome showed nearly all reads clustered within a very small part of the pAdEasy-1 sequence and the adenovirus genome (Fig 4A). When breaking down the pAdEasy-1 sequence and adenovirus genome into overlapping bins (5000bp bins with 2500bp overlap), an average of 95% of all mapping reads aligned to a region that makes up less than 1.5% of the total pAdEasy-1 sequence (Fig 4B and 4C). This ~420bp region is adjacent to the multiple cloning site of the pAdEasy-1 plasmid, and therefore is immediately downstream of inserted DNA for both constructs (GFP and HBV-GFP). The sequence correlating to the mapped reads encompasses the coding region for adenovirus protein IX (pIX), which is a minor adenoviral capsid protein that may play a role in packaging of the adenovirus genome [53]. Previous studies have shown that the use of the CMV promoter to drive expression of genes inserted into pAdEasy-1 can also cause expression of pIX, and at a higher level than other promoters tested in the pAdEasy-1 vector [54]. This correlates well with our findings, as AdGFP-HBV-infected cells have higher levels of pIX transcript compared to AdGFP-infected cells, and expression of HBx has been previously shown to activate the CMV promoter [55]. Our data suggests that pIX is the only adenoviral transcript that is expressed with any abundance, supporting the use of this system as a mechanism for the delivery of exogenous DNA with minimal adenoviral transcript expression.

### Differential-expression analysis

We next examined differential-gene expression across our dataset. For differential-expression analysis, all samples were analyzed together within DESeq2, followed by individual comparisons within the uninfected cells (e.g. uninfected 0hr vs uninfected 24hr) and within the infected cells (e.g. AdGFP 48hr vs AdGFP-HBV 48hr). In addition, differentially expressed genes (DEG), defined as genes with an adjusted p-value after multiple testing correction of < 0.05, in each comparison were also intersected with DEG from each other comparison to determine the number of genes that are differentially expressed in both groups (Table 2, upper half of chart). A 2-fold threshold was also sometimes used to emphasize biological significance, and number of overlapping DEG with ≥ 2-fold change in both comparisons was also calculated (Table 2, bottom half of chart). For infection comparisons, AdGFP was treated as the control to negate any potential adenovirus-mediated effects.

**Table 2:** Number of overlapping differentially expressed genes between comparisons.

	Individual Comparisons			Overlapping Genes Between Comparisons											
Row	Comparison	DE genes	2-Fold Change	U0 to U24	U0 to U48	U0 to U72	U24 to U48	U24 to U72	U48 to U72	G48 to H48	G72 to H72	G48 to G72	H48 to H72	G48 to H72	H48 to G72
1	Uninf 0hr to Uninf 24hr	10245	5846	-	8764	8662	7196	7470	5078	2749	4071	5202	5543	5914	5731
2	Uninf 0hr to Uninf 48hr	11056	6740	4602	-	9957	7907	8137	5589	2980	4364	5714	6117	6471	6289
3	Uninf 0hr to Uninf 72hr	11323	6862	4377	5771	-	7891	8401	5734	2934	4399	5874	6263	6605	6418
4	Uninf 24hr to Uninf 48hr	9431	3284	1801	2482	2407	-	7993	4956	2660	3815	5079	5482	5759	5582
5	Uninf 24hr to Uninf 72hr	10188	4177	2106	2692	3020	2556	-	5445	2663	4051	5616	5885	6100	6146
6	Uninf 48hr to Uninf 72hr	6698	1586	793	1110	1150	916	1181	-	2157	3034	5488	4800	4764	5396
7	GFP 48hr to HBV 48hr	3323	115	60	84	90	71	73	44	-	2064	2205	2341	2580	2534
8	GFP 72hr to HBV 72hr	5070	512	256	333	371	256	325	175	56	-	3065	3320	3732	3460
9	GFP 48hr to GFP 72hr	6973	1814	841	1172	1239	1001	1272	1268	77	215	-	5289	5316	5705
10	HBV 48hr to HBV 72hr	7447	1899	876	1239	1244	1009	1173	1054	54	215	1246	-	6135	5460
11	GFP 48hr to HBV 72hr	7910	2007	947	1262	1305	1007	1184	1006	56	261	1274	1458	-	5152
12	HBV 48hr to GFP 72hr	7713	2132	996	1391	1418	1149	1446	1286	65	242	1493	1393	1143	-

*Number above “-”indicates total DEG in common between indicated comparisons. Number below “-”indicates number of these DEG with at least a 2-fold change.

*Abbreviations: U = Uninfected, G = AdGFP-infected, H = AdGFP-HBV-infected

### Culture-dependent expression changes

**Fig 5:**
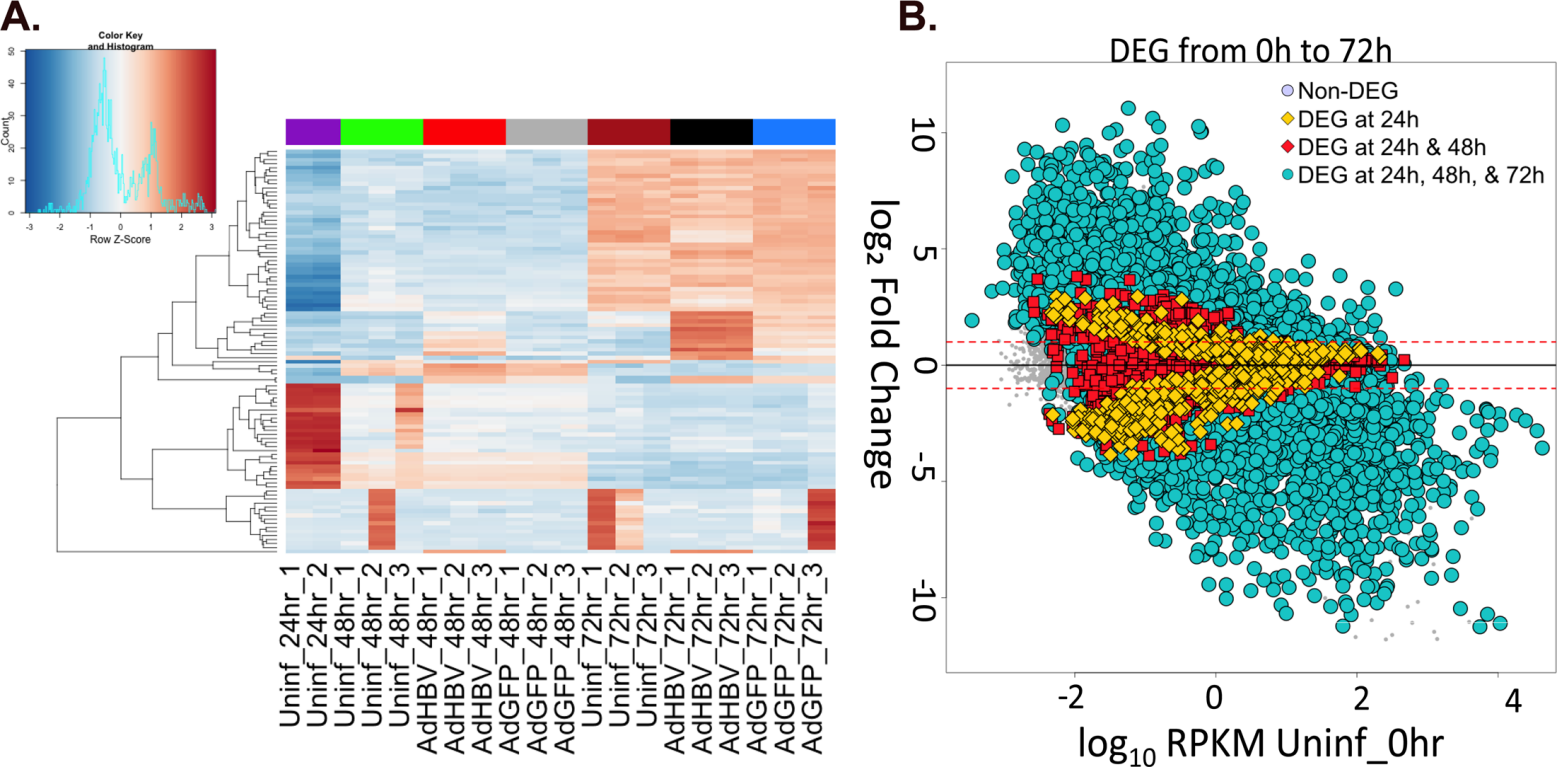
Differential-gene expression due to time in culture. **A.** The top 100 most variable genes across all samples were visualized by heatmap. Gene-expression variation was calculated by Z-score, with red indicating an increase in expression compared to the mean across all samples, and blue indicating a decrease in expression. Uninfected 0hr samples were removed from the analysis to prevent a heavy bias due to the large changes within the first 24hr. **B.** Gene-expression changes in uninfected PRHs over time were plotted as the log_10_ RPKM value for genes in uninfected 0hr samples versus the log_2_ fold change compared to uninfected 0hr for genes in the indicated comparison. Yellow diamonds indicate DEG that were only differentially expressed in the first 24hr, red squares represent DEG at 24hr and 48hr but not 72hr, and blue circles represent DEG at 24hr, 48hr, and 72hr.

We first analyzed differential gene expression by investigating which genes had the highest variance from the mean expression value across all samples, i.e. those with the highest Z-scores. Within the top 100 genes with the highest Z-scores, the majority exhibited time-dependent changes in gene expression. This is clearly seen by the clustering of samples by time point, with treatment-type clustering within each time point set (Fig 5A).

Pairwise comparisons of different time points in the uninfected cells support the observation of time as the principal variable in the dataset (Table 2, rows 1–6). Within the first 24hr after plating the PRHs, there were 10,245 DEG, and this trend continued upwards as time progressed resulting in 11,323 DEG between purification of PRHs at 0hr and 72hr after plating. In fact, the shift between 0hr and 24hr was large enough that the inclusion of the 0hr samples in the analysis of expression variation distorted the clustering of samples to the point that all non-0hr time points looked nearly identical by comparison (S2 Fig). Since the 0hr time point samples were collected immediately after hepatocyte purification without spending any time in culture, a number of these changes are likely the result of cellular adaptation to the culture environment. Two observations support this; first, the overall number of DEG is going down with each 24hr comparison (i.e. 0hr to 24hr, 24hr to 48hr, and 48hr to 72hr), and second, the percentage of DEG with ≥ 2-fold change steadily decreased in each time comparison after the 0hr to 24hr comparison (Table 2).

Although a large number of genes were differentially expressed as a result of purification and plating, when examining the three potential 48hr to 72hr comparisons, ~65% of DEG in the uninfected PRHs in this time comparison are also differentially expressed in this time comparison in both AdGFP- and AdGFP-HBV-infected PRHs. This similarity across treatment types indicates that a specific subset of genes is altered in a time-dependent fashion, regardless of treatment type, and despite minimal morphological changes observed over the time-course of the experiment. However, it is important to note that while 55% of all genes were DEG from 0hr to 72hr, most DEG in this analysis had low levels of expression or small fold changes, which can skew the interpretation of the results. A gene that is not expressed in one sample, but is expressed with only a single read in another, could be interpreted as a DEG, but whether this represents a biologically significant increase is less clear. To that end, only 58% of DEG from 0hr to 72hr had an RPKM value ≥ 1 in all samples and only 24% had an RPKM ≥ 1 and a fold change ≥ 2. Similarly, 61% of the DEG that are in common between all three 48hr to 72hr comparisons have an RPKM value ≥ 1, but only 2% have an RPKM value ≥ 1 and a fold change ≥ 2. For the 48hr to 72hr comparison in particular, these cutoffs alter the interpretation of DEG, as a pathway analysis of the full set of common DEG identified ion binding and intracellular signaling as pathways associated with the these DEG, but a pathway analysis of only the DEG with an RPKM value ≥ 1 and a fold change ≥ 2 identified multiple cell-cycle and DNA-replication pathways. Overall, this highlights the importance of considering differential expression analyses in the context of the biological significance of the changes identified, especially when these include genes expressed at very low levels.

By analyzing differential-gene expression over the time course of our experiment, we established a baseline understanding of what is happening to cultured PRHs after isolation from the liver. Based on the large number of DEG, the process of culturing and maintaining PRHs is a significant variable in the use of this model, and these changes are important to address, although, as described above, the biological relevance of changes to minimally expressed genes must also be part of this consideration. For example, pathway analysis of DEG in uninfected, time-based comparisons identified multiple metabolic processes, as well as pathways associated with focal adhesions, as being significantly altered under these conditions. On the other hand, while the number of DEG seems high between the 0hr and later time points, these changes are not completely reflective of the overall transcriptome. For example, analyzing the correlation between the total expression profiles of 0hr and 24hr uninfected PRHs showed a very high correlation (r^2^ = 0.87). A relatively high correlation (r^2^ = 0.65) is even maintained between 0hr and 72hr uninfected PRHs, despite 55% of the genes being identified as differentially expressed between these samples. Similar correlations were previously seen when comparing cultured hepatocytes to *in vivo* liver samples, and is in stark contrast to the correlation between hepatocyte cell lines and *in vivo* liver samples (r^2^ = 0.1) [41], supporting the use of the PRH model as a biologically relevant system. However, similar to acknowledging that cell lines express a transcriptome that is significantly different than a normal hepatocyte, it is important to consider that cultured PRHs are also changing due to experimental conditions, regardless of treatment.

### HBV-mediated expression changes

Although the largest sets of DEG were seen when comparing time points, HBV had a distinct effect on gene expression (Table 2, rows 7 & 8). For example, 3,323 genes were differentially expressed when comparing expression between 48hr AdGFP-HBV-infected PRHs and 48hr AdGFP-infected PRHs. Of these, only 115 had a ≥ 2-fold change, including several cell cycle regulators previously linked to HBV-mediated changes in hepatocyte physiology. These include *CDK2*, *CDK1*, *CCNE1* (Cyclin E1), and *CCNE2* (Cyclin E2) [7, 10, 15, 56] (and reviewed in [18]). When comparing expression at 72hr, there were 5,070 DEG between AdGFP-infected PRHs and AdGFP-HBV-infected PRHs, and 512 of these had ≥ 2-fold change (Table 2). In particular, 25 members of the solute carrier (SLC) family of transmembrane proteins had an HBV-mediated ≥ 2-fold change at 72hr, including up-regulation of Glut1 (*SLC2A1*), and down-regulation of Glut2 (*SLC2A2*). Glut2 is the primary transporter of glucose between the blood and the liver, and has previously been reported to be down-regulated by HBV [34].

**Fig 6:**
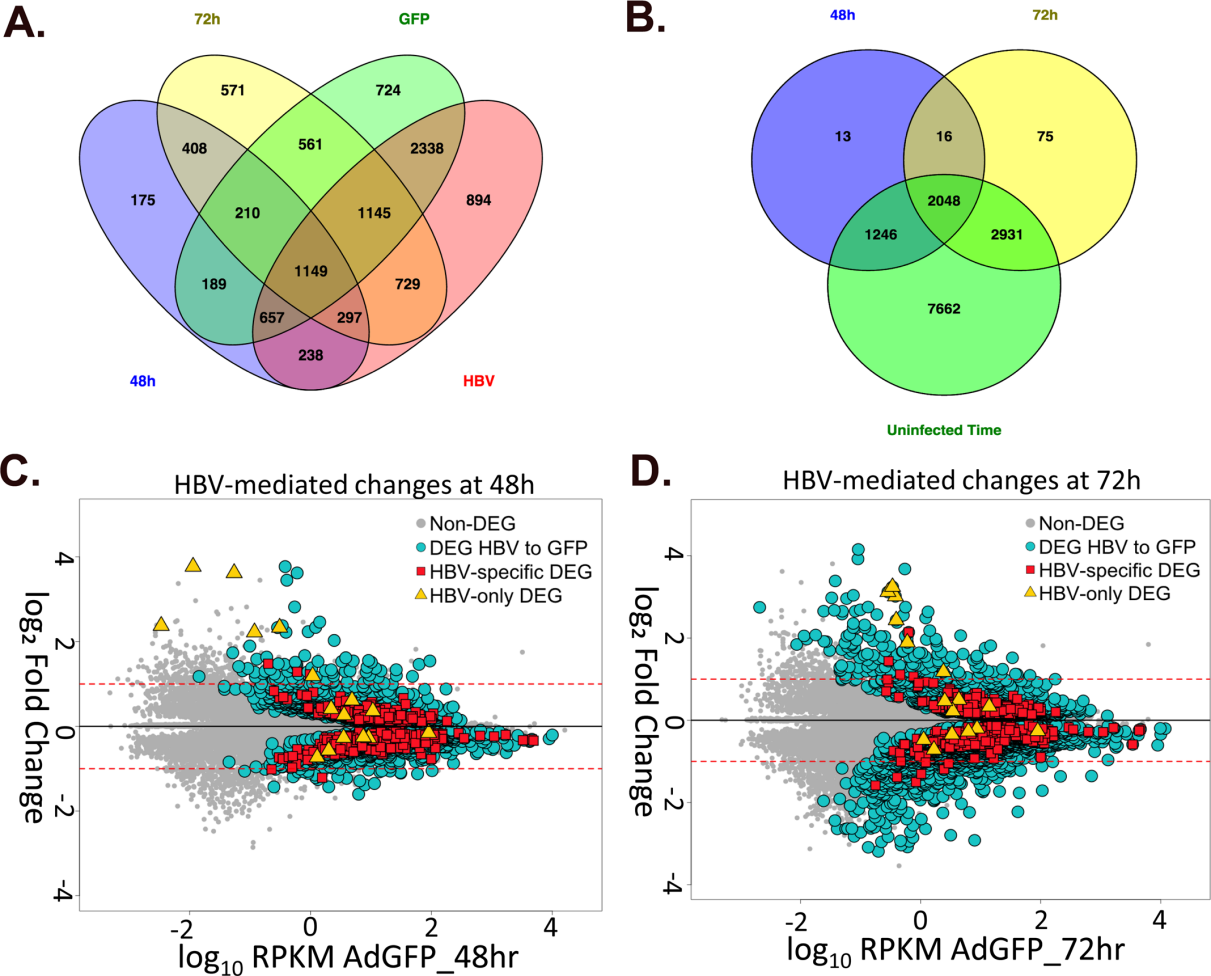
Overall analysis of HBV-mediated changes in gene expression. **A.** Venn diagram of DEG from AdGFP 48hr to AdGFP-HBV 48hr (blue), AdGFP 72hr to AdGFP-HBV 72hr (yellow), AdGFP 48hr to AdGFP 72hr (green) and AdGFP-HBV 48hr to AdGFP-HBV 72hr (red) comparisons. The "HBV-specific" subset (described in text) is circled. **B.** Venn diagram of DEG from AdGFP 48hr to AdGFP-HBV 48hr (blue), AdGFP 72hr to AdGFP-HBV 72hr (yellow), and any DEG from all Uninfected/time-mediated comparison (green). The "HBV-only" subset is circled. **C-D.** HBV-mediated gene-expression changes were plotted for 48hr samples (C.) and 72hr samples (D.) as the log_10_ RPKM value for genes in AdGFP samples versus the log_2_ fold change compared to AdGFP for genes in the indicated comparison. Yellow diamonds indicate DEG in the "HBV-only" subset, red squares represent DEG in the "HBV-specific" subset, and blue circles represent all DEG in the AdGFP to AdGFP-HBV comparison at the indicated time point.

Of the total HBV-mediated changes, 2,064 genes were differentially expressed at both 48hr and 72hr, including *CDK1* and two of the SLC genes (*SLCO5A1*, *SLC29A*). 56 of these genes had a ≥ 2-fold change at both time points (Table 2, row 8), including down-regulation of *Wnt11*, an important regulator of cell fate that has been associated with development of HCC [57], and up-regulation of *G0s2*, which was originally described as a regulator of early stages of the cell cycle, but is now well established as being involved in the regulation of lipolysis and activation of apoptosis [58–60] (and reviewed in [61]). To further isolate changes in gene expression that are specifically mediated by HBV, genes that were also differentially expressed in corresponding time-mediated comparisons (AdGFP 48hr to 72hr, AdGFP-HBV 48hr to 72hr) were removed, resulting in 408 genes that are differentially expressed at both 48hr and 72hr solely due to the expression of HBV (Fig 6A and 6B, red squares, and 6C, yellow and blue overlap). This subset of genes, described as the "HBV-specific" subset, is listed with expression level and fold change values in S2 Table.

**Fig 7:**
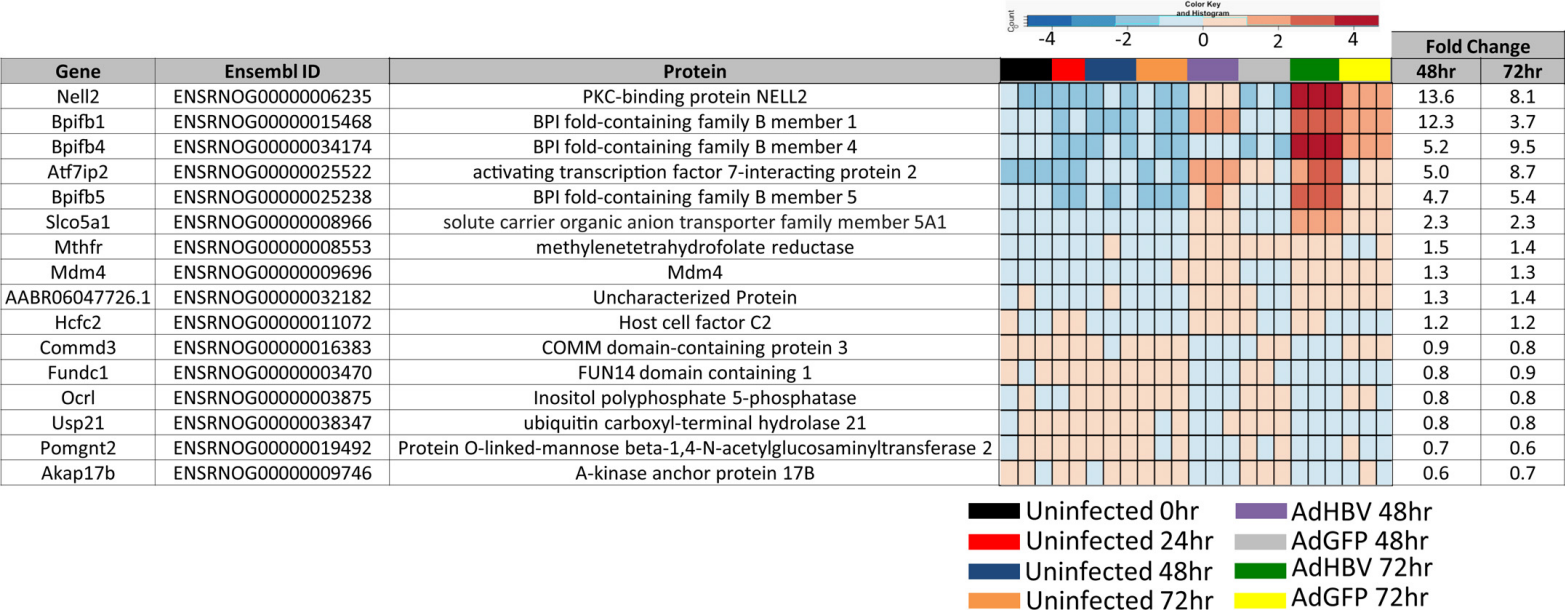
Description of HBV-only subset of differentially expressed genes. Gene name, Ensembl ID, and associated protein name are given for genes within the HBV-only subset of genes. Fold changes comparing expression in AdGFP to AdGFP-HBV at the indicated time points is included, along with a heatmap depicting expression variation (by Z-score) across all samples.

When genes that are differentially expressed in any of the uninfected time-mediated comparisons were removed, this left a small subset of 16 genes that specifically change as a result of HBV expression, but not due to time spent in culture (Fig 6A and 6B, yellow triangles, and 6D, yellow and blue overlap). These genes, referred to as the "HBV-only" subset, are described in Fig 7. Despite their HBV-mediated regulation in this study, none of the 16 DEG in the HBV-only subset has been previously shown to be directly regulated by HBV, although some of the DEG are within pathways that have been reported to be regulated by HBV. For example, Mdm4 is involved in apoptosis and p53 regulation, both of which have previously been associated with HBV replication (reviewed in [18]). Of additional interest was the identification of three BPI family B (BPIFB) proteins. These proteins are involved in lipid binding and innate immunity, and have very low endogenous expression in PRHs that increases dramatically in HBV-expressing PRHs.

### HBV effect on time-mediated changes

While identifying genes that exhibit expression changes solely due to the presence of HBV is of particular interest, simply removing genes that exhibit time-dependent expression changes is likely too strict of a selection criteria (the “HBV-only” and “HBV-specific” subsets above). This is because analyzing HBV-mediated changes in the context of time-dependent changes can also yield important information about the cellular impact of HBV replication, which becomes apparent when analyzing the 48hr to 72hr comparisons across treatment types.

When comparing uninfected PRHs at 48hr and 72hr, 6,698, or 32% of all expressed genes, are significantly differentially expressed (Table 2, row 6). The same 48hr to 72hr comparison in AdGFP-infected PRHs results in 4% more (6,973 total) DEG, which could be partially explained due to sample-to-sample variation (Table 2, row 9). In addition, the presence of adenovirus in this system could potentially induce some changes in gene expression. Adenovirus can activate the innate immune response, along with other cellular pathways, through the binding of cell surface receptors (reviewed in [62]), although the minimal expression of adenoviral transcripts in our system (Fig 4) suggests adenovirus is not likely having a significant direct effect on the cells.

In contrast, when examining the AdGFP-HBV-infected 48hr to 72hr comparison (Table 2, row 10), ~16% more genes (7,447 total DEG) were differentially expressed than in uninfected PRHs, and 11% more than in AdGFP-infected PRHs. When examining overlap between these three subsets of DEG, 82% of DEG in uninfected PRHs between 48hr and 72hr were also differentially expressed in AdGFP-infected PRHs between 48hr and 72hr; however, only 72% of DEG were shared between uninfected and AdGFP-HBV-infected cells in the same time comparison. This means that despite the fact that more genes were differentially expressed in AdGFP-HBV-infected PRHs, fewer DEG were in common with uninfected PRHs. Overall, these comparisons show that although changes in gene expression occur over time in culture, the presence of HBV clearly affects the expression of genes that also exhibit time-dependent expression changes.

**Fig 8:**
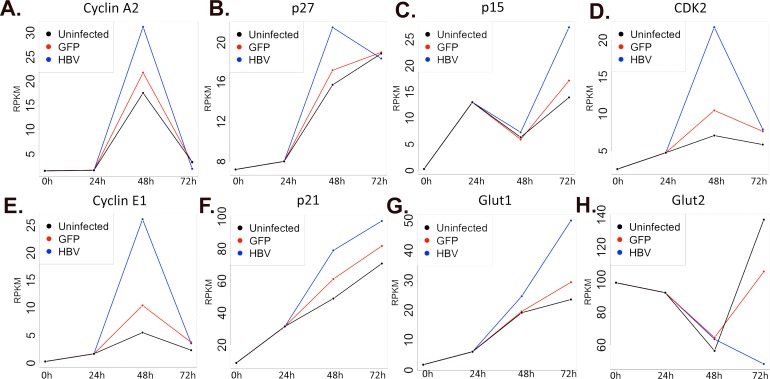
HBV-mediated impact on time-mediated, differential-gene expression. **A-H.** Individual gene expression was plotted as RPKM value over time for uninfected PRHs (black), AdGFP-infected PRHs (red), and AdGFP-HBV-infected PRHs (blue).

To further illustrate the complexity involved in determining the potential importance of genes with time-mediated changes in expression in the context of HBV infection, we directly examined the expression of a number of cell-cycle regulatory genes. These genes, including Cyclin A2 (*Ccna2*), p21 (*Cdkn1a*), p27 (*Cdkn1b*), Cdk1, and Cyclin E1, are each differentially expressed in a time-mediated fashion in uninfected cells (Fig 8). As the data suggest, the process of isolating and plating the PRHs seems to be moving the PRHs out of G_0_ and into the active cell cycle, with a number of genes either steadily increasing or peaking at 48hr after plating. If the PRHs are undergoing changes that are allowing them to adapt to culture conditions, as suggested by the large shift in gene expression in uninfected PRHs, altered expression of genes closely involved in regulating the cell cycle is expected. When the infected PRHs are considered, similar patterns of expression are maintained; however, the level of alteration to expression is often more dramatic in the presence of replicating HBV. For example, while *Ccna2* drops 6.7-fold (from an RPKM of 21.8 to 3.3) between 48hr and 72hr in AdGFP-infected PRHs, the drop is nearly 15-fold (from an RPKM of 31.2 to 2) in AdGFP-HBV-infected PRHs (Fig 8A). In uninfected PRHs, *Ccna2* is expressed at an RPKM of about 1.5 before increasing up to 17.6 at 48hr and returning down to 3.4 at 72hr, demonstrating that while there is a distinct time-mediated pattern of expression for this gene, the presence of HBV has a significant impact on gene expression as well. Such HBV-mediated differences would be ignored if time-dependent factors serve as exclusion criteria for potentially HBV-mediated changes in gene expression.

### Confirmation of differential gene expression

Multiple methods of validation were used to confirm differential expression of genes in this transcriptome analysis. As one method, we conducted an additional overall comprehensive confirmation by completing an independent RNA-seq experiment in AdGFP- and AdGFP-HBV-infected PRHs (S3 Fig). By assessing the expression of all genes across the entire PRH transcriptome an additional time, we are able to more accurately verify the observed HBV-mediated changes to the primary hepatocyte transcriptome. This experiment also served as an independent biological replicate to rule out effects specific to a single rat or a single preparation of AdGFP-HBV or AdGFP (S4 Fig and S3 Table). Although, overall, there were fewer HBV-mediated effects on gene expression in the second dataset, this difference could actually be due to different cDNA library preparation methods, something that has previously been described to influence RNA-seq results [63]. However, despite the lower level of overall gene expression changes, a high degree of overlap existed between the two datasets. Specifically, of the 547 HBV-mediated DEG at 48hr in the second dataset, 80% (435) were also differentially expressed in the primary dataset in the same comparison. Similarly, of the 2,122 DEG at 72hr in the second dataset, 70% (1,476) were also differentially expressed in the same comparison in the primary dataset. The overlap between these two datasets is similar to the amount of overlap seen when analyzing the primary dataset with multiple differential expression analysis pipelines. For example, 76% of DEG between AdGFP- and AdGFP-HBV-infected PRHs at 48hr in the primary dataset were identified as DEG when using edgeR instead of DESeq2 (S1 Fig and S1 Table). In fact, 55% of DEG in the 48hr comparison were identified as differentially expressed in all four pipelines used (S1 Fig). Importantly, of the 16 genes identified as being regulated specifically by HBV in the primary dataset (Fig 7), 13/16 were DEG in the BWA pipeline, 15/16 were DEG in the edgeR pipeline, and 12/16 were DEG in the Tuxedo pipeline. While minimal, the variation between these pipelines represents differences in how the algorithms deal with individual data points and supports the interpretation that much of the "noise" in the primary dataset, and when comparing datasets, could be attributed to genes with low expression levels or to genes with small fold changes that are handled differently across algorithms.

**Fig 9:**
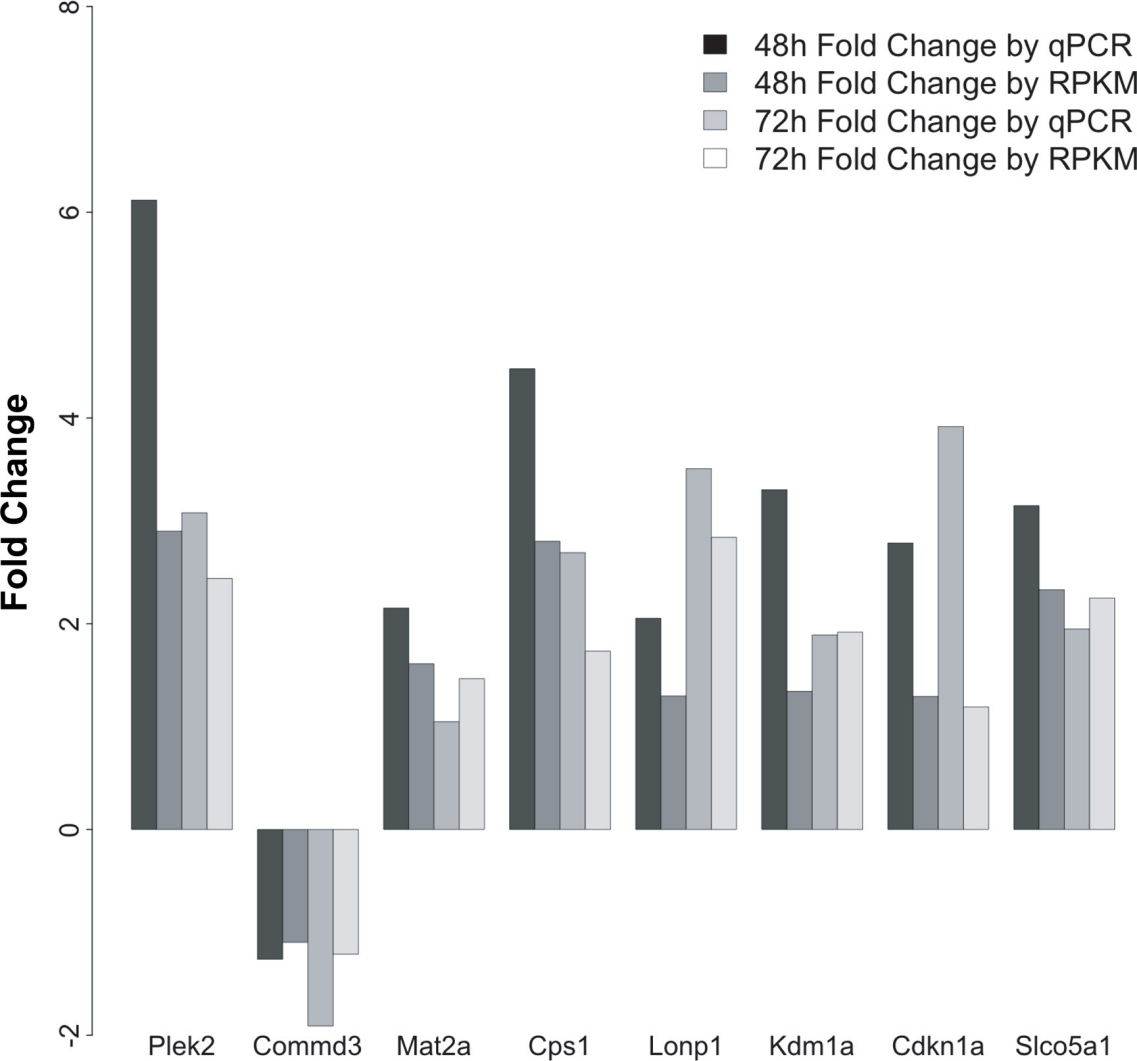
Confirmation of differentially expressed genes by qRT-PCR. Expression of genes from the "HBV-only" and "HBV-specific" was confirmed by qRT-PCR (bars labeled qPCR). Expression is presented as fold change of AdGFP-HBV-infected cells compared to AdGFP-infected cells at the indicated time point. Fold change using the RPKM values described in the primary RNA-seq dataset was included for comparison of expression patterns (bars labeled RPKM).

As another approach to confirm expression of DEGs in the same samples utilized for the primary RNA-seq dataset, altered expression was confirmed by qRT-PCR for genes from multiple subsets within the primary dataset, including the "HBV only" and "HBV specific" subsets. Importantly, the genes investigated by qRT-PCR demonstrated a similar pattern of HBV-mediated regulation as was seen in the transcriptome analysis (Fig 9), including significant reduction of the "HBV only" gene *Commd3*.

Finally, a similar analysis of a small set of DEG was also conducted in cultured primary human hepatocytes as a confirmation of the relevance of observed HBV effects in PRHs (S5 Fig). For this analysis, cultured primary human hepatocytes were infected with either AdGFP or AdGFP-HBV 24hr after plating. Due to limited sample availability, only the 48hr time point (24hr after infection) was collected. In this context, adenovirus was used to deliver the HBV genome instead of direct infection with HBV due to the overall low efficiency of infection of cultured primary human hepatocytes with purified HBV, while adenovirus infections can achieve nearly 100% infection efficiency. This is an important aspect of these studies, as the early stages of an *in vivo* HBV infection result in a high percentage of infected hepatocytes, something that is not achieved with direct HBV infection of cultured primary human hepatocytes. Moreover, the low efficiency of HBV infection in cultured primary human hepatocytes would likely prevent an accurate transcriptome analysis as most of the cultured human hepatocytes would not be HBV-infected. Although only two human hepatocyte samples were used, and therefore there was not enough power to confidently determine statistical significance, the pattern of HBV-mediated gene regulation in AdGFP-HBV-infected primary human hepatocytes resembles that seen for these genes in primary rat hepatocyte samples (Fig 9). Specifically, four out of five genes analyzed by qRT-PCR in primary human hepatocyte samples mimicked the pattern of expression seen when examining these genes in HBV-expressing PRHs. These data, in combination with our previously published studies, support the use of PRHs as a surrogate model for studying effects of HBV in human hepatocytes.

Overall, the overlap between the primary and secondary RNA-seq datasets, the identification of DEG by multiple analysis pathways, and the confirmation of a subset of DEG by qRT-PCR support the results of our RNA-seq analysis. These results represent an important dataset describing the significant impact of HBV on hepatocyte gene expression, which will serve as an important tool in understanding the network of hepatocyte signaling pathways that are affected during an HBV infection.

### Pathway analysis

To analyze cellular pathways impacted by HBV, we utilized the iPathwayGuide analysis tool, which uses an "Impact Analysis" to identify differentially regulated pathways, biological functions, and cellular processes. This analysis scores pathway impact based on overrepresentation of DEG as well as the impact of differential expression of a gene across all of the annotated interactions of the gene [64]. A number of cellular pathways or biological functions previously associated with HBV replication was identified as being altered in the presence of HBV (Table 3). As expected based on the differential-expression analysis described above, cell cycle and related cellular processes were some of the most affected cellular processes. This result is of particular interest because of the well-established role of cell-cycle regulation in HBV replication. While some cell-cycle-related processes were altered at both time points, the impact of HBV on the cell cycle was most obvious at the earlier infection time-point (48hr). On the other hand, metabolic processes were also significantly impacted by HBV, but more so at the later infection time point (72hr), when pathways and processes, including PI3K/AKT signaling and lipid and amino acid metabolism, were significantly perturbed. HBV-mediated disruption of metabolic pathways has been previously shown by RNA-seq analyses in multiple model systems [34, 65].

**Table 3:** HBV-mediated pathway perturbation.

Pathway	p-value at 48hr	p-value at 72hr
ECM-receptor interaction	0.068	< 0.001
Central carbon metabolism in cancer	0.167	< 0.001
Carbon metabolism	0.143	< 0.001
Nitrogen metabolism	0.434	0.001
Butanoate metabolism	0.538	0.002
Metabolic pathways	0.654	0.002
Glycolysis / Gluconeogenesis	NA	0.002
PI3K-AKT signaling pathway	**0.038**	**0.002**
Biosynthesis of amino acids	**0.013**	**0.002**
Glycine, serine and threonine metabolism	**0.010**	**0.004**
Taurine and hypotaurine metabolism	NA	0.004
Bile secretion	0.104	0.004
Focal adhesion	**0.021**	**0.004**
Galactose metabolism	NA	0.005
Aminoacyl-tRNA biosynthesis	0.244	0.006
Arginine and proline metabolism	0.563	0.006
Hematopoietic cell lineage	**0.022**	**0.007**
Fructose and mannose metabolism	0.666	0.007
Amoebiasis	0.691	0.009
Glycosphingolipid biosynthesis	0.306	0.009
Glycosphingolipid biosynthesis	0.410	0.009
Glycosphingolipid biosynthesis	0.410	0.009
Drug metabolism	0.539	0.010
Calcium signaling pathway	0.127	0.010
Neuroactive ligand-receptor interaction	0.168	0.012
Tryptophan metabolism	NA	0.013
Small cell lung cancer	0.086	0.017
PPAR signaling pathway	0.415	0.019
Rap1 signaling pathway	0.853	0.024
Insulin secretion	0.490	0.026
Glycosphingolipid biosynthesis	0.306	0.026
Glycosphingolipid biosynthesis	0.410	0.026
Glycosphingolipid biosynthesis	0.410	0.026
Maturity onset diabetes of the young	0.341	0.031
Cytokine-cytokine receptor interaction	0.570	0.033
Retinol metabolism	0.123	0.038
Alanine, aspartate and glutamate metabolism	0.083	0.040
Inflammatory mediator regulation of TRP channels	0.942	0.041
Cholinergic synapse	0.140	0.042
Pathways in cancer	**0.033**	**0.043**
Glycosphingolipid biosynthesis	0.306	0.043
Glycosphingolipid biosynthesis	0.410	0.043
Glycosphingolipid biosynthesis	0.410	0.043
Butirosin and neomycin biosynthesis	NA	0.045
Cyanoamino acid metabolism	0.150	0.045
Valine, leucine and isoleucine biosynthesis	0.150	0.045
Glutathione metabolism	0.244	0.046
Vascular smooth muscle contraction	0.179	0.048
Protein digestion and absorption	0.004	0.053
Dilated cardiomyopathy	0.044	0.111
Hypertrophic cardiomyopathy (HCM)	0.030	0.153
Arrhythmogenic right ventricular cardiomyopathy (ARVC)	0.036	0.281
MicroRNAs in cancer	< 0.001	0.339
Fanconi anemia pathway	0.010	0.460
Bladder cancer	0.028	0.520
p53 signaling pathway	0.007	0.738

*NA indicates genes within that pathway were not identified as significantly altered in this at this time point

*Bold indicates pathways that are significantly perturbed in both comparisons

### miRNAs in cancer

One HBV-mediated pathway alteration identified in the iPathwayGuide analysis was "miRNAs in cancer." This is particularly interesting because multiple groups have recently begun to determine the role that miRNAs may have in the HBV life cycle and HBV-associated disease, including HBV-associated HCC (reviewed in [27–29]). Within the results of these published studies, a small subset of miRNAs have been identified more often than others as having HBV-mediated changes in expression. One such miRNA is miR-125a, which was also identified in the current iPathway analysis as a miRNA for which a number of its targets exhibited HBV-mediated differential expression. Interestingly, multiple studies have previously shown up-regulation of miR-125a in HBV-expressing cell lines and HBV-infected patient samples [66], and miR-125a has also been proposed to inhibit HBV replication either through direct targeting of the HBV RNAs or through targeting of cellular mRNAs required for HBV replication [67, 68]. The iPathway analysis also identified multiple members of the let-7 family of tumor-suppressor miRNAs. In the normal liver, four of the top nine total miRNAs expressed are members of the let-7 family, and expression is significantly decreased in undifferentiated cells and cancers, including HCC [69]. In addition, expression of the let-7 family has previously shown HBV-mediated regulation [70–73], in agreement with the effects seen in the iPathwayGuide analysis.

In addition to RNA-seq analysis, total RNA from the secondary dataset (described above) was used to perform a qPCR analysis of expression for a set of 84 liver-enriched miRNAs. This identified only minimal HBV-mediated regulation of miRNA expression occurring in our system (S6 Fig). Specifically, of the 84 miRNAs examined, only miR-224-5p had a ≥ 2-fold change in expression when comparing AdGFP and AdGFP-HBV infected cells at 48hr. Four additional miRNAs had statistically significant (but < 2-fold) HBV-mediated differential expression at this time point (miR-148b-3p, miR-221-3p, miR-222-3p, and miR-378a-3p); all but miR-224-5p were considered significantly altered miRNAs in the iPathwayGuide analysis at 72hr. In fact, miR-378a had the most altered targeting of all miRNAs at 72hr. In addition, both miR-221 and miR-222 have previously been shown to have HBV-mediated regulation.

It is important to note that in identifying "miRNAs in cancer" as a significantly altered cellular pathway, the iPathway analysis is specifically identifying differential expression of miRNA target genes, and not expression of the miRNA itself. Because of the specific steps in cDNA library generation, levels of mature miRNAs cannot be assessed in this transcriptome analysis. For example, miR-125a itself, which is described by the pathway analysis as being an altered miRNA, is not present in the transcriptome dataset. Instead, alteration of a miRNA's "pathway" implies that the iPathway analysis is more specifically assessing the function of miRNAs, instead of relying on analysis of miRNA expression levels, by reporting changes in annotated targets for that particular miRNA. This is a significant distinction because as technology and our understanding of miRNA function and miRNA-mediated regulation has improved, it has become important to consider the targeting profile of miRNAs, and not just their overall expression level. For example, while only minimal HBV-mediated changes to miRNA expression were identified in these studies, it is possible that HBV could be significantly altering the function of specific miRNAs by influencing the mRNAs being targeted by specific miRNAs. This interpretation is supported by the identification of differential expression of target genes associated with miRNAs whose expression level does not seem to change in our cell-culture system. In addition, previous studies investigating the impact of HBV on the profile of miRNA expression using next-generation sequencing of HBV-infected liver samples identified only minimal HBV-mediated changes to miRNA expression levels [69, 74]. This is contrary to the significant level of HBV-mediated changes identified in various cell-culture models using immortalized cell lines [71, 75, 76] and further supports the use of cultured primary hepatocytes, and our model system, as a more biologically relevant model than immortalized cell lines.

## Discussion

HBV is a non-cytopathic virus that establishes a chronic infection that can last for decades. For many chronically infected individuals, the endpoint of disease is the development of HBV-associated HCC. Because of this, many HBV-related studies attempt to identify HBV-mediated changes to the host cell that give the cell a more cancer-like phenotype. These studies, however, often do not take the time-frame of HCC development into consideration; if HBV causes dramatic tumorigenic changes immediately after infection, HCC likely would take weeks or maybe years to develop, instead of decades. In fact, HBV-mediated changes to any one factor or pathway are likely not dramatic, and may be overshadowed by cumulative changes over the course of disease progression. Our transcriptome analysis supports this hypothesis; at 48hr (24hr after infection) only 3% of DEG had a ≥ 2-fold HBV-mediated change, and at 72hr (48hr after infection) this was 10%. On the other hand, a more drastic cellular insult, such as isolation of hepatocytes and adaptation to culture may be expected to cause more dramatic cellular changes. This is supported by the observation that 57% of DEGs between 0hr and 24hr in uninfected PRHs have ≥ 2-fold change. The observation that HBV induces subtle changes to hepatocytes underscores the importance of working in a model system that closely mimics normal hepatocyte physiology. This is an important aspect of our studies, which were specifically designed as an unbiased approach to identify HBV-mediated changes to primary hepatocytes that occur early after an HBV infection and, although we are limited by the experimental system in our interpretation of long-term changes, may influence both our understanding of HBV replication and downstream HBV-associated disease. Hence, a goal of these studies was to provide a better understanding of the complex network of host-virus interactions in HBV-infected hepatocytes.

In analyzing changes to the PRH transcriptome, it became immediately obvious that both the initial isolation and plating of hepatocytes and the amount of time spent in culture have a distinct impact on the hepatocyte transcriptome. Although the number of DEG is high when comparing freshly isolated hepatocytes to later time points, this is not entirely unexpected and has been reported previously for cultured primary hepatocytes [41]. As with many organs, the *in vivo* environment of the liver has a number of distinct characteristics that make it difficult to replicate in a cell culture system. In particular, the liver is a collection of multiple cell types, and while hepatocytes make up the large majority of cells in the liver, our culture model lacks the additional cell types that have previously been shown to help maintain primary hepatocyte viability and differentiation [77]. In addition, only around 1 in every 20,000 hepatocytes is undergoing replication at any given time in a normal liver [6]; however, previous studies have shown activation of pathways resembling liver regeneration, including entry into the G_1_ phase of the cell cycle, following liver perfusion and hepatocyte isolation [56, 78, 79]. While minimal rounds of replication occur after plating of primary hepatocytes, some of these replicative pathways do appear to be activated. Evidence of this can be seen by the up-regulation of cell-cycle-related and DNA-replication-related genes in our uninfected cultured PRHs over time. On the other hand, despite the time-mediated changes, primary hepatocytes remain a much more physiologically relevant model than most immortalized and transformed cell lines. In particular, transformed cell lines, which are often used for liver-based studies, have drastically different phenotypes than primary hepatocytes or *in vivo* samples. By definition, these cell lines retain many characteristics that were affected by the tumorigenic environment from which they were originally isolated, particularly the ability to replicate indefinitely. For example, while a recent RNA-seq analysis of HBV-mediated gene expression changes in the Huh7 cell line did identify metabolic pathways as being altered by HBV, only minimal identification of cell cycle-related DEG was reported [34]. This may be due to the altered baseline of cell cycle regulation occurring in these transformed cells, limiting the ability to detect alteration to genes whose expression is already drastically altered. Such changes are more easily identified in a primary hepatocyte model, as time-mediated changes which separate cultured primary hepatocytes from freshly isolated cells can be directly identified and serve as a baseline for expression changes, instead of the endogenously altered baseline present in cell lines. In fact, multiple studies have demonstrated the significant phenotypic differences between primary hepatocytes and cell lines [41, 80], which were also recently supported by a transcriptome analysis of the HepG2 hepatoblastoma cell line. This study compared HepG2 cells to primary liver tissue and confirmed that the majority of up-regulated genes were associated with cancer and cellular proliferation pathways, prompting the authors to warn against the use of HepG2 cells in liver cancer-related studies of the transcriptome [37]. On the other hand, while we do identify time-mediated gene expression changes in our PRHs model, these numbers are likely biased by genes with a low expression level or small fold change. This is supported by the high correlation when the complete gene expression profile in cultured PRHs is compared between 0hr samples and later time points. Based on this, and on the data reported here, the use of primary hepatocytes seems to be an appropriate cell-culture-based model for identifying alteration of cellular pathways, including those altered in the context of HBV infection.

A potential concern about our studies is the use of recombinant adenovirus as a delivery vehicle for the HBV genome. However, we were able to analyze two important aspects of the use of the recombinant adenovirus-based system: the impact of an adenovirus infection on the PRH transcriptome and the level of expression of adenoviral transcripts. The results of the differential gene expression analysis suggest that adenovirus infection had only a minimal effect on the PRH transcriptome. This is most obvious when infected PRHs are compared directly to uninfected PRHs. Specifically, at 48hr only 3% of genes were differentially expressed between AdGFP-infected and uninfected PRHs, while 25% of genes are differentially expressed between AdGFP-HBV-infected and uninfected PRHs. In addition, alignment of reads to the pAdEasy sequence showed expression of only a single adenoviral transcript, pIX, which is immediately downstream of the insertion site of the GFP/HBV DNA. In fact, only ~5% of all reads aligning to pAdEasy-1 aligned outside of this region, demonstrating that expression of adenoviral transcripts, which could be a concern when using these vectors, is minimal in our system. These results allow us to attribute the majority of AdGFP-HBV-mediated effects to the presence of HBV, as adenovirus seems to be having minimal, if any, impact on the cells.

The results of our RNA-seq analyses represent an extensive dataset describing the impact of HBV on hepatocyte gene expression, which can be used to drive future research towards understanding the overall network of host-virus interactions and contribute to the development of novel therapeutic strategies. In this report, we have described, as an example, a small subset of ways in which this dataset can be utilized, such as the description of the "HBV-only" and "HBV-specific" subsets. The applications of this dataset extend well beyond these subsets of genes, however, and this dataset will be used in the future to investigate many additional aspects of the host-virus interactions. An example of this includes the identification of regulatory nodes directly impacted by HBV, which ultimately result in the altered expression of subsets of downstream genes. These nodes could represent important potential points of therapeutic intervention.

Interestingly, applying this dataset to previously published HBV-related studies also acts to confirm both our results and the biological relevance of these findings. For example, a number of DEG identified in these studies, such as the cell-cycle regulators Cyclin A, Cdk2, and Cyclin D [7, 9], the metabolic factor Glut2 [34], the immune mediators PD-L1 [81], and PI3K/AKT signaling [20] have previously been reported to be affected by HBV. In the case of cell cycle regulators and PI3K/AKT signaling, we have previously shown HBV-mediated alterations of associated proteins and the role of these proteins in regulating HBV replication[8, 20]. Corroborating results were seen when investigating these genes in our transcriptome dataset. Importantly, while these published results were initially done in PRHs, with the results correlating well with our PRH RNA-seq results, each of these results were also confirmed in HBV-expressing primary human hepatocytes. This serves as significant support for the biological relevance of both the PRH system and the HBV-mediated changes to hepatocyte gene expression that were identified in our RNA-seq analyses.

For many other differentially expressed genes that we report in this study, the association with HBV replication is novel, highlighting the utility of using an unbiased, transcriptome-wide approach to identify cellular changes on a broader scale. While only one aspect of this extensive dataset, the list of 16 "HBV-only" genes offers an interesting set of genes altered in an HBV-dependent, time-independent fashion. Of these 16 genes, none has previously been shown to be affected by HBV. Three of these 16 are members of the BPI family [82, 83], which are typically associated with lung tissue and saliva and play a role in lipid binding and transfer. In our analysis, these three identified DEG exhibit an interesting pattern of very little endogenous expression, which was increased at least 4.5-fold at both time points for all three genes in the presence of HBV. Additional lipid-related genes, such as *G0s2*, were also significantly altered by HBV at both time points. This relationship between HBV and cellular lipids is an interesting point of further research. For example, cellular lipids are required for viral envelopment [84], and studies have shown that decreased cellular cholesterol alters the HBV envelope protein and inhibits envelopment of the HBV genome-containing capsid [85]. Our pathway analysis identified multiple cholesterol-related pathways as being altered by HBV, and further investigation into viral utilization of cellular lipid pathways could be an important step towards fully understanding methods of capsid envelopment and particle secretion.

Much of the previous HBV-mediated gene expression profiling work has focused on identifying immune mediators responsible for influencing the development from acute to chronic disease and factors involved in viral clearance. For example, this is true for interesting work in chimpanzees directly infected with HBV [86], as well as studies of both acute [38] and chronic [30] WHV infection in woodchucks. While our experimental system limits our ability to investigate the response of immune mediators to HBV, analysis of a number of factors suggests a correlation between our findings and these *in vivo* datasets. For example, a trend exists within our dataset suggesting that many of the genes identified as having peak expression in the “middle” of viral clearance in HBV-infected chimpanzees are similarly induced by HBV at 24 hr post-infection in our system. This includes genes encoding PI3K, pituitary tumor-transforming gene 1, which has previously been associated with expression of HBx [87], solute carrier 7A7, Rab proteins (*RAB20*, *RAB27A*, *RAB31*), and ribonucleotide reductase M2, among others. Similarly, a number of genes identified in the study of WHV-infected woodchucks are also differentially expressed in our studies. Importantly, while we would not expect to see *PD-1* expression in our system, we do see an HBV-mediated increase in *CD274* (PD-L1) expression, as was seen in both chronic and acute WHV infection, as well as in HBV-infected patients [88]. Together, these studies support the overall relevance of our findings, and offer support in our interpretation of HBV-mediated differential gene expression.

Technological advances allowing the generation of "-omics" data have greatly increased the large-scale generation of data. In addition to the studies described here, additional application of these types of broad techniques could help to generate a more complete understanding of the changes in hepatocyte physiology induced by an HBV infection. Such goals could be achieved through the combination of transcriptome-wide and proteomic studies to better understand the role that gene regulation plays in HBV-mediated changes to the hepatocyte proteome. This relationship between gene regulation and protein regulation has become increasingly important in light of recent findings suggesting that specific cellular conditions, specifically pathogen exposure, can impact the relative importance of mRNA abundance and protein production in the cell [89]. These findings seem to correlate, at least phenotypically, with the results suggested by our PRH transcriptome analysis. For example, while significant changes to cell-cycle-related genes were seen in our analysis, there were no noticeable changes to the levels of cell proliferation. Further investigation could determine the role, if any, HBV is playing in directly influencing the relationship between mRNA abundance and protein production.

Similarly, recent studies have also suggested that the cellular environment can influence the profile of functional miRNAs without impacting the overall levels of miRNAs [90, 91], which is again supported by the results described here and in other studies [69, 74]. We showed minimal changes in miRNA expression, though transcriptome analysis of annotated miRNA targets suggested altered function of multiple miRNAs which have been previously associated with HBV and HBV-associated HCC. Future research utilizing newer technologies that directly assess the profile of functional miRNAs, instead of profiling the overall expression level of miRNAs, could help to determine the role that miRNAs play in HBV replication and HBV-associated pathogenesis.

While our studies attempt to address the early events that result in the cellular changes associated with replicating HBV, some caveats do exist. For example, while the use of primary hepatocytes instead of cell lines increases the relevance of our studies, using purified hepatocytes does mean that additional cell types of the liver are not present. Although hepatocytes make up 80% of the cellular mass of the liver [92], and cultured primary hepatocytes are a well-accepted model of liver biology, future *in vivo* studies investigating the early time-points of an HBV infection may help to further strengthen our understanding of HBV-mediated transcriptome changes. Similarly, the use of recombinant adenovirus to deliver the HBV genome bypasses the infection-related steps of the HBV life cycle. The use of transgenic animals expressing the recently-described HBV receptor, sodium taurocholate cotransporting peptide(NTCP/*SLC10A1*), may be the next step in addressing HBV-mediated transcriptome-wide changes in gene expression that follow direct infection of hepatocytes, although significant hurdles with post-entry species restriction limit the current application of these models. Finally, because of the technical limitations of the experimental system, this represents a transient model of HBV replication and limits the interpretation of the long-term impact of HBV replication on hepatocyte gene expression. Despite these caveats, the studies described here are an important step in understanding the HBV-mediated affects on hepatocyte physiology. Additional studies using these datasets will help to identify specific factors that may be playing a central role in HBV replication, and therefore in the development of HBV-associated pathogenesis, allowing the identification of potential targets for the development of novel therapeutics.

## Materials and Methods

### Animal studies

Surgery and isolation of hepatocytes from rats were approved by the Institutional Animal Care and Use Committee of the Drexel University College of Medicine (Protocol # 20057) and complied with the Animal Welfare Act, the Public Health Service Policy on Humane Care and Use of Laboratory Animals, and the NIH Guide for the Care and Use of Laboratory Animals (2011).

### Primary rat hepatocyte isolation and maintenance

PRHs were isolated from 5–7 week old male Sprague-Dawley rats following a two-step perfusion protocol which has been described in detail elsewhere [93, 94]. For culture and maintenance of PRHs, cells were plated on 6cm tissue culture dishes coated with rat-tail collagen (~200μg/ml). PRHs were maintained in William's E medium (Life Technologies, Carlsbard, CA) supplemented with 2.0 mM L-glutamine, 1.0 mM sodium pyruvate, 4.0 μg/ml Insulin/Transferrin/Selenium, 5.0 μg/ml hydrocortisone, 5.0 ng/ml epidermal growth factor, 10 μg/ml gentamycin, and 2% dimethyl sulfoxide and maintained at 37°C in 5% CO_2_ as previously described; medium was changed daily.

### Maintenance of primary human hepatocytes

Normal primary human hepatocytes were obtained in suspension from the Liver Tissue Cell Distribution System (Pittsburgh, PA). This distribution system is supported by NIH contract number HHSN276201200017C. Approximately 1.5 x 10^6^ primary human hepatocytes were plated per collagen-coated well in a 6 well plate with Williams E medium, as described for primary rat hepatocytes.

### Recombinant adenovirus infections

The construction of the recombinant adenoviruses, containing either hrGFP alone (AdGFP) or hrGFP and a greater than unit length copy of the HBV serotype ayw genome (AdGFP-HBV), has been described previously [46]. The infection protocol, along with detailed descriptions of determination of the amount of virus to use for infection, has also previously been described [46, 47]. Briefly, because recombinant adenovirus does not replicate in PRHs, the exact amount of HBV delivered on a per-cell basis cannot be directly determined. Instead, recombinant adenovirus titers are calculated on Ad293 cells to ensure an equal amount of virus is used. Then, knowing the titer of virus in Ad293 cells, PRHs are infected with viral titrations to determine the amount of virus needed to give the desired infection efficiency, in this case ~80%, based on expression of GFP. All samples are then infected with the same amount of recombinant adenovirus. When calculated strictly considering the titer determined in Ad293 cells, an M.O.I. of 0.01 was required for infecting 80% of PRHs and this relative M.O.I. was used for infection. It is important to note, however, that this M.O.I. can only be considered as a relative number because of the increased susceptibility of PRHs to adenovirus infection as compared to Ad293 cells and the fact that the recombinant adenovirus titer must be determined in Ad293 cells. Since recombinant adenovirus does not replicate in PRHs, any GFP expression can be directly attributed to delivery of hrGFP DNA by infection of PRH with GFP-expressing recombinant adenovirus. Similarly, because HBV can replicate in, but not infect, PRH, any HBV replication can be directly attributed to delivery of HBV DNA by infection with HBV genome-containing recombinant adenovirus.

For these studies, approximately 3.5 x 10^6^ cells were plated per sample in a 6cm plate and infected with AdGFP-HBV or AdGFP 24hr after plating. Cells were infected in 150μl total volume at a relative M.O.I. of approximately 0.01, based on titer values determined in Ad293 cells, and allowed to incubate for 1hr with rocking every 15mins. After infection, 3ml of culture medium was added to the cells, and cells were incubated overnight at 37°C with 5% C0_2_. Medium was changed daily. Infection efficiency was monitored by GFP expression using an EVOS FL cell imaging system (Life Technologies, Carlsbad, CA), and HBV replication was analyzed by Southern blot for HBV core particle-associated DNA as described previously [95].

### RNA extraction, cDNA synthesis, and sequencing

For the primary dataset, hepatocytes were isolated from a single rat liver, and total RNA was isolated from triplicate samples of uninfected, AdGFP-HBV- or AdGFP-infected PRHs at indicated time points for a total of 24 samples. The 0hr time point refers to PRHs that were isolated and purified, but not plated, while all other samples were plated and maintained for the indicated time. RNA was isolated from all samples following the total RNA protocol from the mirVana RNA isolation kit (Life Technologies, Carlsbad, CA). Total RNA was submitted to the Drexel University College of Medicine Center for Genomic Sciences (DUCOM CGS) for quality control, polyA selection, cDNA library preparation, and sequencing. The cDNA library was prepared using the TruSeq Stranded mRNA Prep Kit (Illumina), and was sequenced using the Illumina NextSeq 500 platform over two runs with 75 cycle High Output kits (ver. 1). One uninfected 24hr sample was lost during cDNA library creation, leaving a final total of 23 samples that were sequenced.

For the secondary dataset, total RNA was isolated using the mirVana RNA isolation kit from triplicate samples of PRHs; these PRHs were isolated from a separate rat liver and were infected with different preparations of AdGFP and AdGFP-HBV than were used to generate the primary dataset. The PRHs in the secondary dataset were infected with either AdGFP or AdGFP-HBV 24hr after plating. Total RNA was divided for use in downstream miRNA analyses (see below) or for quality control, polyA selection, cDNA library preparation, and the first round of sequencing by Genewiz, Inc (South Plainfield, NJ). cDNA library preparation was done using the NEBNext Ultra RNA Library Preparation Kit (New England Biolabs, Ipswich, MA), and the first round of sequencing was done using the Illumina HiSeq2500 platform to generate 1 x 50bp reads. The cDNA library was then used for a second round of sequencing by the DUCOM CGS using the Illumina NextSeq 500 platform to generate an additional set of 1x75bp reads. Analysis of sample variation within the secondary dataset showed one sample, a GFP 48hr replicate, was an outlier and was removed from subsequent analysis. Downstream analysis of differential gene expression showed high overlap between the Genewiz and DUCOM CGS sequencing results within the secondary dataset. This similarity supported merging the aligned reads into a single alignment file per sample consisting of both the Genewiz- and DUCOM CGS-generated reads that was used for the downstream differential gene analysis of the secondary dataset.

The quality of the sequencing reads from all datasets was confirmed using FastQC (v0.11.2) [96].

### Mapping of RNA-seq reads

For read alignments, a custom genome was created in which HBV serotype ayw (NCBI accession X02496.1), hrGFP (extracted from NCBI accession EU048698.1), and the pAdEasy-1 adenoviral vector (NCBI accession AY370909.2) sequences were concatenated to the Rnor_5.0 build of the *Rattus norvegicus* reference genome (Ensembl, 5.0.78, updated 07–2014). A custom annotation was similarly built from downloaded GFF files, but with pAdEasy-1 and hrGFP treated as single features.

Several alignment algorithms were used to independently map all samples to the custom reference. Specifically, the Burrows-Wheeler Aligner mem algorithm (BWA, v0.7.10) [97], the STAR aligner (v2.4.0h) [98], and TopHat2 (v2.0.13) [99] utilizing Bowtie2 (v2.2.4) [100] were each used in conjunction with downstream analysis tools to independently arrive at a set of differentially expressed genes. Importantly, only uniquely mapping reads were retained by filtering with Sambamba (v0.5.0) [101] using a mapping quality of ≥1 for BWA, 255 for STAR, or 50 for TopHat. General mapping statistics were generated using the Sambamba flagstat tool or by the STAR aligner.

### Generating count tables

A series of Bioconductor packages for R (v3.1.2, "Pumpkin Helmet") [102] were used to create count tables reporting the number of reads mapping to each transcript in the reference gene set using either the BWA or STAR alignments. Specifically, these packages were RSamtools (v1.18.2) [103], GenomicFeatures (v1.18.3), and GenomicAlignments (v1.2.1) [104]. The output of this workflow was a data table with counts per annotated feature per sample, suitable for direct use in the differential expression analysis. In addition, counts per feature were normalized using the reads per kilobase of transcript per megabase library size (RPKM) method [105] by dividing total counts aligned to each feature by the kilobase length of that feature and the total number of millions of reads in the sample. As independent confirmation of results of the BWA/STAR pipelines, the "Tuxedo" pipeline [106] was also utilized to determine differential gene expression. For this pipeline, TopHat-aligned reads were assembled into a set of expressed transcripts, and counts of these transcripts were determined using Cufflinks (v2.2.1).

### Differential gene expression analysis

While independent differential gene expression analyses were tested, the majority of differential gene expression analysis was done using the DESeq2 (v1.6.3) [107] package in R. Individual contrasts (e.g. AdGFP-HBV 24hr vs AdGFP 24hr, Uninfected 0hr vs Uninfected 24hr) were conducted within the DESeq2-generated object on raw counts normalized by the median ratio method [108], and statistical significance was determined after multiple testing correction and calculation of fold changes based on a fitted model of normalized counts[107]. Similarly, differential gene analysis was also determined using the edgeR (v3.8.5) [109] package in R. For both analyses, significant differential gene expression was determined using an FDR adjusted p-value of 0.05 (adjusted with the Benjamini-Hochberg adjustment [110]). Venn diagrams were generated using the Venny tool [111]. Additional data visualization was done using R, including the ggplot2 package (v1.0.0) [112]. To follow general convention, and to give an estimated idea of expression levels, plotting was done using RPKM values, however, statistical significance was generated using the median ratio normalized values within DESeq2 and edgeR.

For the Tuxedo pipeline, differential expression analysis was performed using the Cuffdiff package. Each assembled transcript file, along with the reference transcriptome, was merged to a single annotation file using the Cuffmerge package. DEG were then determined using Cuffdiff by supplying the treatment groupings (Uninfected 0hr, Uninfected 24hr, Uninfected 48hr, Uninfected 72hr, GFP 48hr, HBV 48hr, GFP 72hr, HBV 72hr), custom reference genome, the transcript quantifications from Cufflinks, and the merged annotation file (Cuffmerge output). Significant differential expression was determined with a q-value of 0.05, and Cuffdiff output was visualized using the CummeRbund package (v2.8.2) for R.

### HBV and adenovirus genome coverage

Coverage per base of both the HBV genome and the pAdEasy sequence was determined using bedtools coverage (v2.22.0) [113]. For the purposes of plotting HBV, the bases of the HBV genome were reordered relative to the polyA signal, so that 10 bases after the last base of the polyA signal became base 3182, and the 11th base after the polyA signal was used as base 1. It has previously been reported that the HBV transcripts continue for 10bp after the polyA signal [52]. In addition, relative coverage across the HBV genome was determined using bedtools coverage to quantitate total reads mapping to 172bp bins sliding in 86bp increments across the entire genome. For the pAdEasy sequence, relative coverage was determined by counting reads in 5000bp bins in 2500bp increments, with the final 2 bins containing only the bases up to the final base of the sequence.

### qRT-PCR

For quantitative polymerase chain reaction (qPCR), 5 μg of total RNA (described above for primary dataset) was reverse transcribed using M-MuLV reverse transcriptase (New England Biolabs) with an oligo d(T)_18_ primer. qPCR analysis was then done on 100ng of cDNA using Power SYBR Green 2X mastermix (Life Technologies) with 250nM forward and reverse primers (S4 Table). PCR was performed using a BioRad CFX96 real-time thermocycler with the following cycling conditions: an initial denaturation at 95° for 10 min followed by 40 cycles of 95° for 15 sec and 55° for 30 sec. Fold change was determined using the 2^-ΔΔC(t)^ method.

### Pathway analysis and gene ontology

Two tools were used for pathway enrichment and gene ontology analysis. Pathway analysis on specific gene subsets, such as within time point comparisons, was done using the Biomart online enrichment tool (www.biomart.org) [114]. Additional pathway analysis involving full datasets was carried out using the iPathwayGuide online tool (Advaita Bio, http://www.advaitabio.com/ipathwayguide). Briefly, differential expression analysis results from DESeq2 were uploaded to the iPathwayGuide tool. The uploaded data contained differential expression analysis results for each comparison, across all genes. A fold change of 1.5 and an adjusted p-value of 0.05 were used as cut-offs for significant differences in expression. Pathways were considered significantly altered by an Impact Analysis, a combination of over-representation analysis and pathway perturbation [64, 115]. For over-representation analysis, the number of altered genes associated with a pathway was compared between treatment and control groups. To determine pathway perturbation, the significance of a particular gene to a pathway was considered in determining the overall impact on the pathway by examining all annotated functions/interactions of the gene.

### Liver miRNA qPCR array

Expression of a panel of liver-enriched miRNAs was determined using the Liver miFinder miRNA qPCR array platform (Qiagen). For each sample, 1 μg total RNA (described above for secondary dataset), was reverse transcribed using the miScript RT II kit following the manufacturer's protocol(Qiagen). cDNA quality was assured using the miScript miRNA QC qPCR array plate (Qiagen) before proceeding to miRNA expression analysis. For qPCR arrays, each cDNA sample was applied to a single 96-well plate, and qPCR was carried out according to the manufacturer's protocol using a Bio-rad CFX96 thermocycler. Cycling conditions were 95° for 10 min, followed by 40 cycles of 94° for 15 sec, 55° for 30 sec, and 70° for 30 sec with a 1°C/sec ramp speed. Data analysis and differential expression were determined using the miScript miRNA PCR array data analysis tool (Qiagen, http://pcrdataanalysis.sabiosciences.com/mirna/arrayanalysis.php).

### Data accession

RNA sequencing data from both datasets was deposited in the Gene Expression Omnibus and is available as a GEO SuperSeries using accession number GSE68113.

## Supporting Information

S1 Fig Confirmation of differential gene expression through multiple RNA-seq analysis pipelines. A. Diagram of multiple analysis pipelines used. B-C. Venn diagram indicating overlap of differentially expressed genes identified using the STAR -> DESeq2 pathway, STAR -> edgeR pipeline, BWA -> DESeq2, or Tuxedo suite. Comparisons are for DEG identified between AdGFP-infected or AdGFP-HBV-infected PRHs at 48hr (B) or 72hr (C).
(TIF)

S2 Fig Heatmap of most variable genes across complete dataset. Heatmap of the 100 most variable genes across all samples based on Z-score.
(TIF)

S3 Fig RNA-seq overview for secondary dataset. **A.** Euclidean sample distance was mapped allowing unbiased ordering of samples based on sample similarity. **B.** Plot of distribution of average RPKM values per sample.
(TIF)

S4 Fig Confirmation of differential gene expression by independent RNA-seq. **A-D.** Venn diagrams indicating DEG overlap between primary (light) and secondary (dark) datasets for AdGFP-HBV to AdGFP at 48hr (A) and 72hr (B), AdGFP 48hr to 72hr, and AdGFP-HBV 48hr to 72hr (D). Percent overlap indicated represents number of DEG identified in secondary dataset that are also differentially expressed in the primary dataset.
(TIF)

S5 Fig Confirmation of differential gene expression by qRT-PCR in primary human hepatocytes. The expression of a small subset of genes, identified as altered by HBV in primary rat hepatocytes (PRH), was analyzed by qRT-PCR in HBV-expressing or control primary human hepatocytes (PHH). Samples were collected 48hr after plating (24hr after infection), and data is presented as fold change in AdGFP-HBV-infected cells compared to AdGFP-infected cells.
(TIFF)

S6 Fig qPCR analysis of miRNA expression. A-B. Expression of a panel of liver-enriched miRNAs was analyzed by qPCR array and plotted to visualize differential expression at 48hr (A) and 72hr (B) between AdGFP-infected and AdGFP-HBV-infected PRHs. Outer diagonal lines indicate a 2-fold change.
(TIF)

S1 Table Comparison of differential gene expression analysis pipelines. (DOCX)

S2 Table HBV-specific subset of differentially expressed genes. (DOCX)

S3 Table Secondary dataset RNA-seq overview. (DOCX)

S4 Table qPCR primers for detection of differentially expressed transcripts. (DOCX)
