# Supplementary figures and images for: Transcriptome-Wide Analysis of Hepatitis B Virus-Mediated Changes to Normal Hepatocyte Gene Expression
Source: PLoS Pathog. 2016 Feb 18;12(2):e1005438. doi: 10.1371/journal.ppat.1005438 (PMC4758756; doi:10.1371/journal.ppat.1005438)

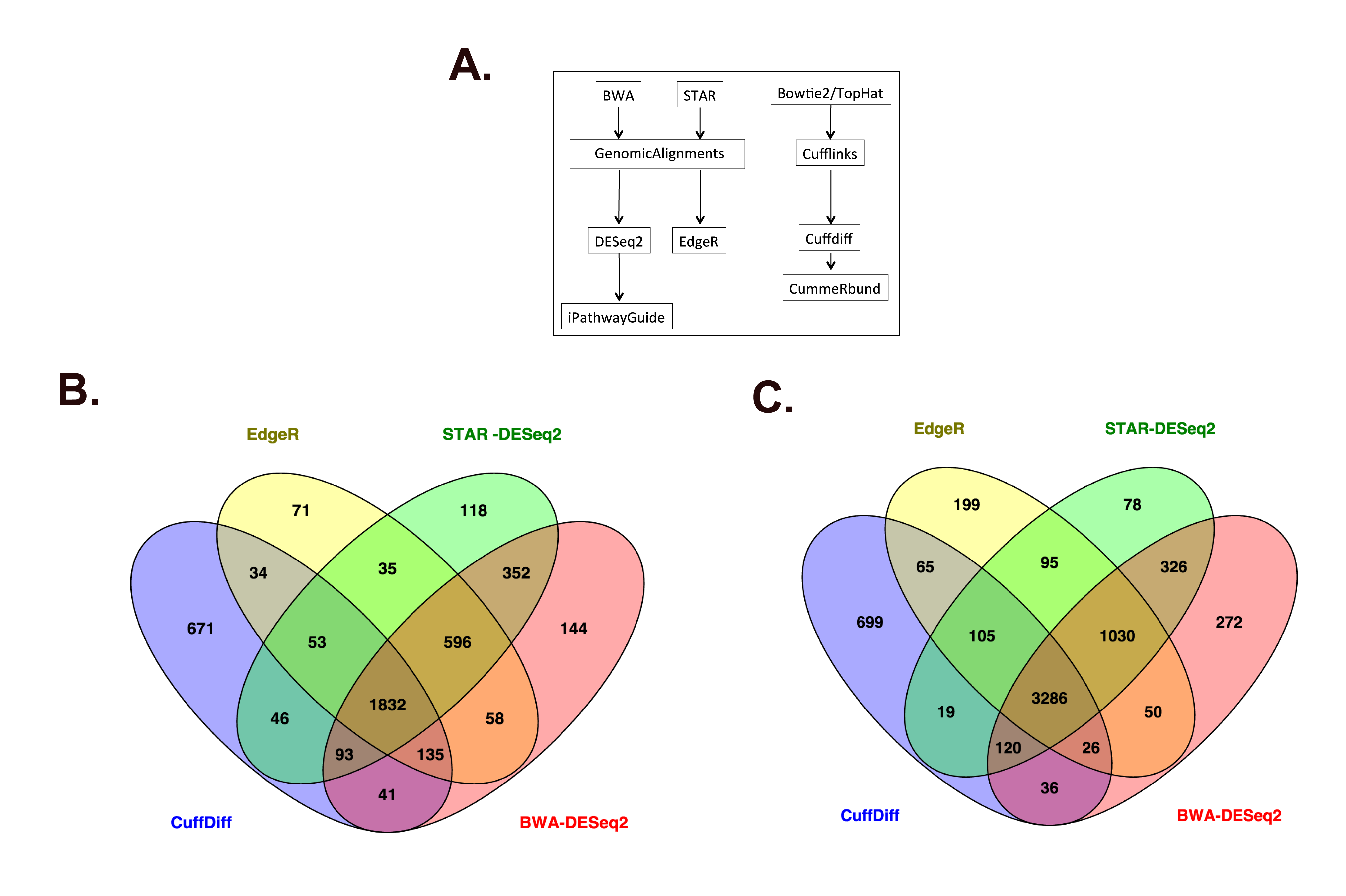

Supplement: S1 Fig — A. Diagram of multiple analysis pipelines used. B-C. Venn diagram indicating overlap of differentially expressed genes identified using the STAR -> DESeq2 pathway, STAR -> edgeR pipeline, BWA -> DESeq2, or Tuxedo suite. Comparisons are for DEG identified between AdGFP-infected or AdGFP-HBV-infected PRHs at 48hr (B) or 72hr (C). (TIF) [file ppat.1005438.s001.tif]

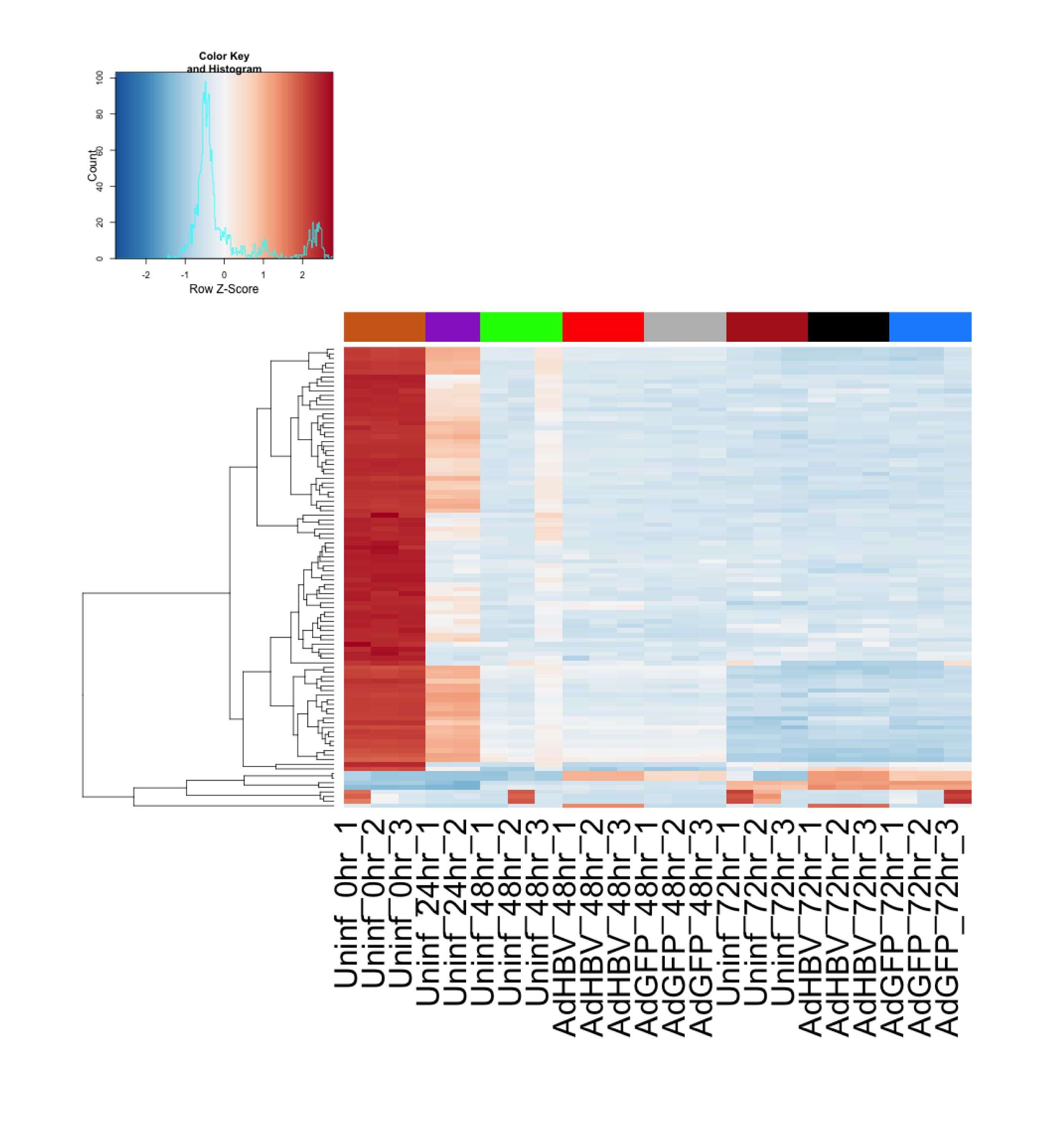

Supplement: S2 Fig — Heatmap of the 100 most variable genes across all samples based on Z-score. (TIF) [file ppat.1005438.s002.tif]

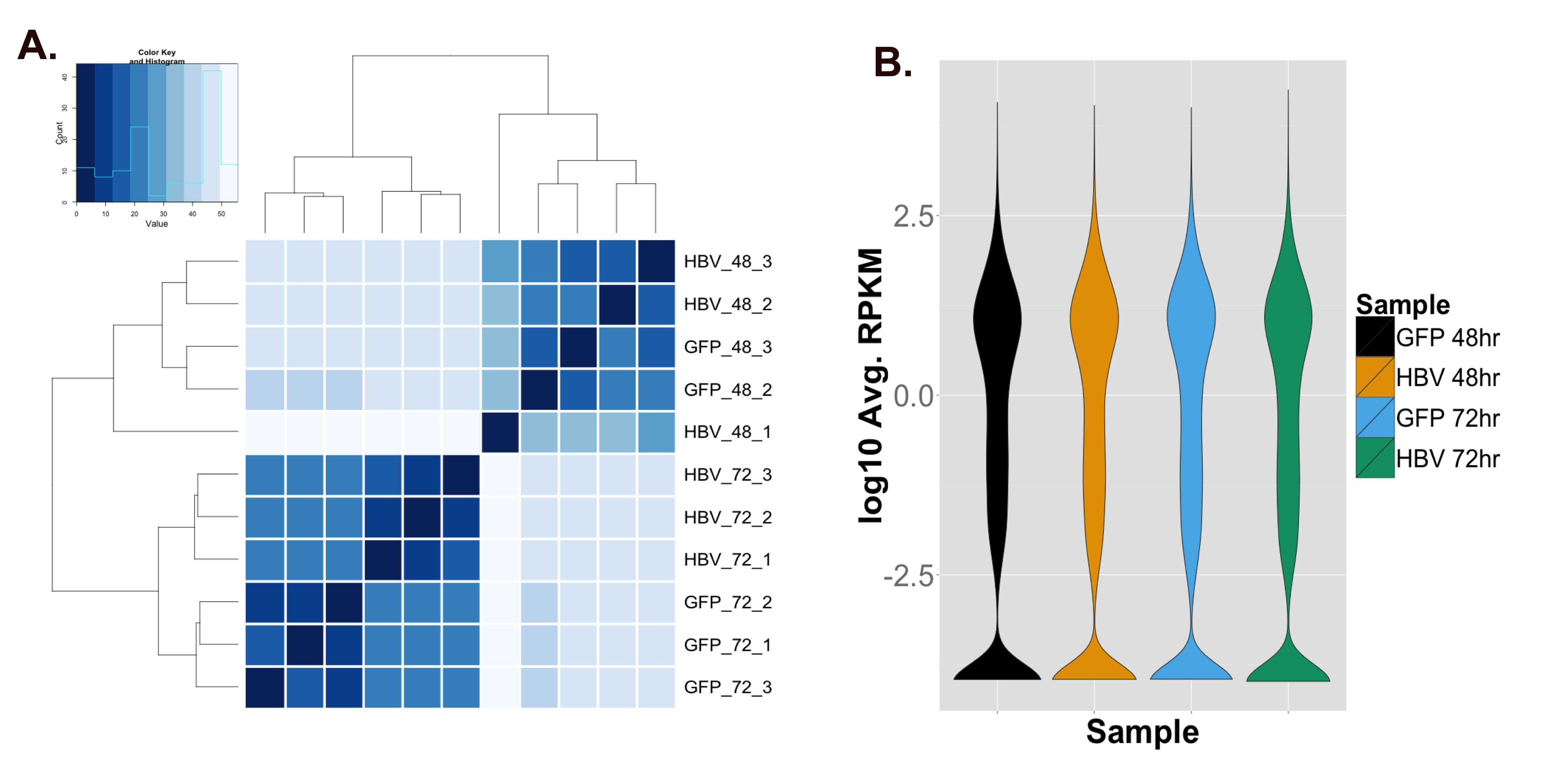

Supplement: S3 Fig — A. Euclidean sample distance was mapped allowing unbiased ordering of samples based on sample similarity. B. Plot of distribution of average RPKM values per sample. (TIF) [file ppat.1005438.s003.tif]

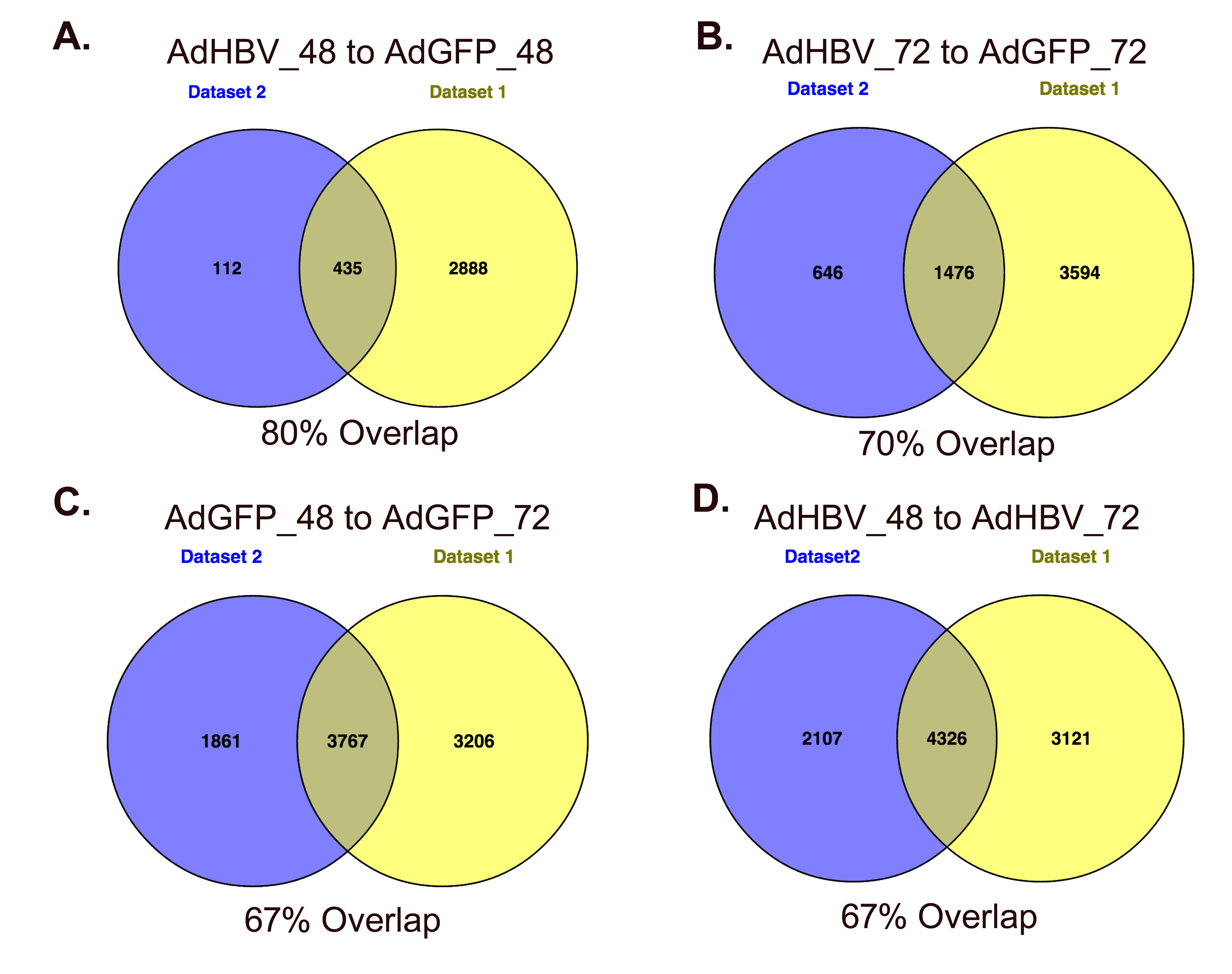

Supplement: S4 Fig — A-D. Venn diagrams indicating DEG overlap between primary (light) and secondary (dark) datasets for AdGFP-HBV to AdGFP at 48hr (A) and 72hr (B), AdGFP 48hr to 72hr, and AdGFP-HBV 48hr to 72hr (D). Percent overlap indicated represents number of DEG identified in secondary dataset that are also differentially expressed in the primary dataset. (TIF) [file ppat.1005438.s004.tif]

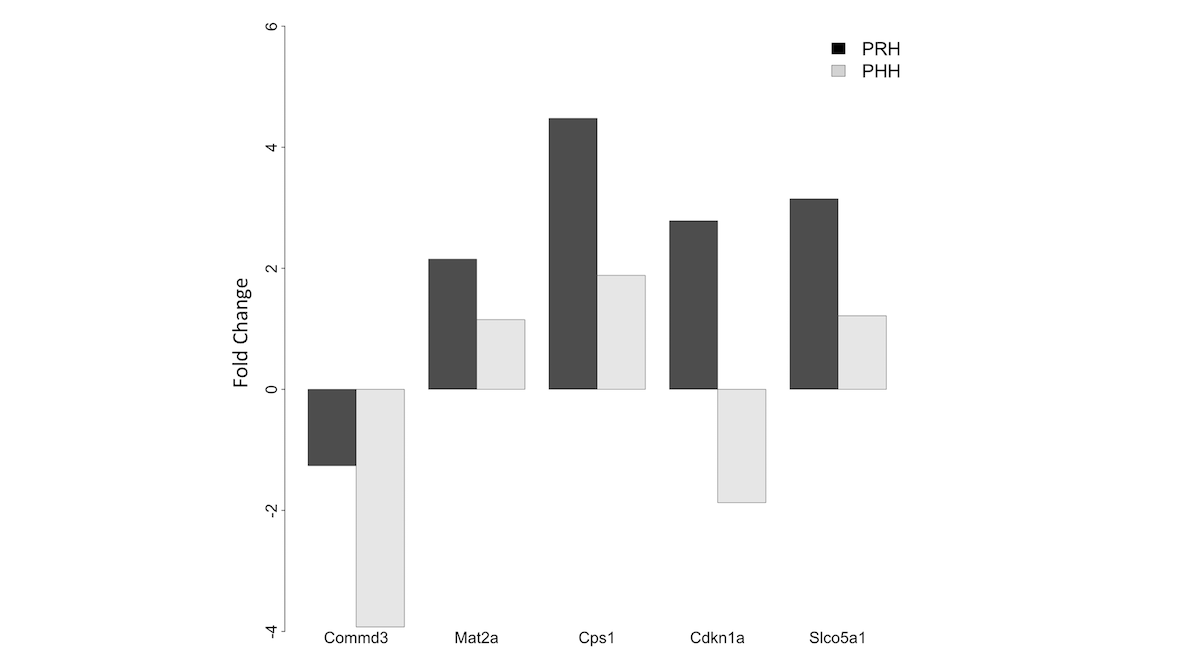

Supplement: S5 Fig — The expression of a small subset of genes, identified as altered by HBV in primary rat hepatocytes (PRH), was analyzed by qRT-PCR in HBV-expressing or control primary human hepatocytes (PHH). Samples were collected 48hr after plating (24hr after infection), and data is presented as fold change in AdGFP-HBV-infected cells compared to AdGFP-infected cells. (TIFF) [file ppat.1005438.s005.tiff]

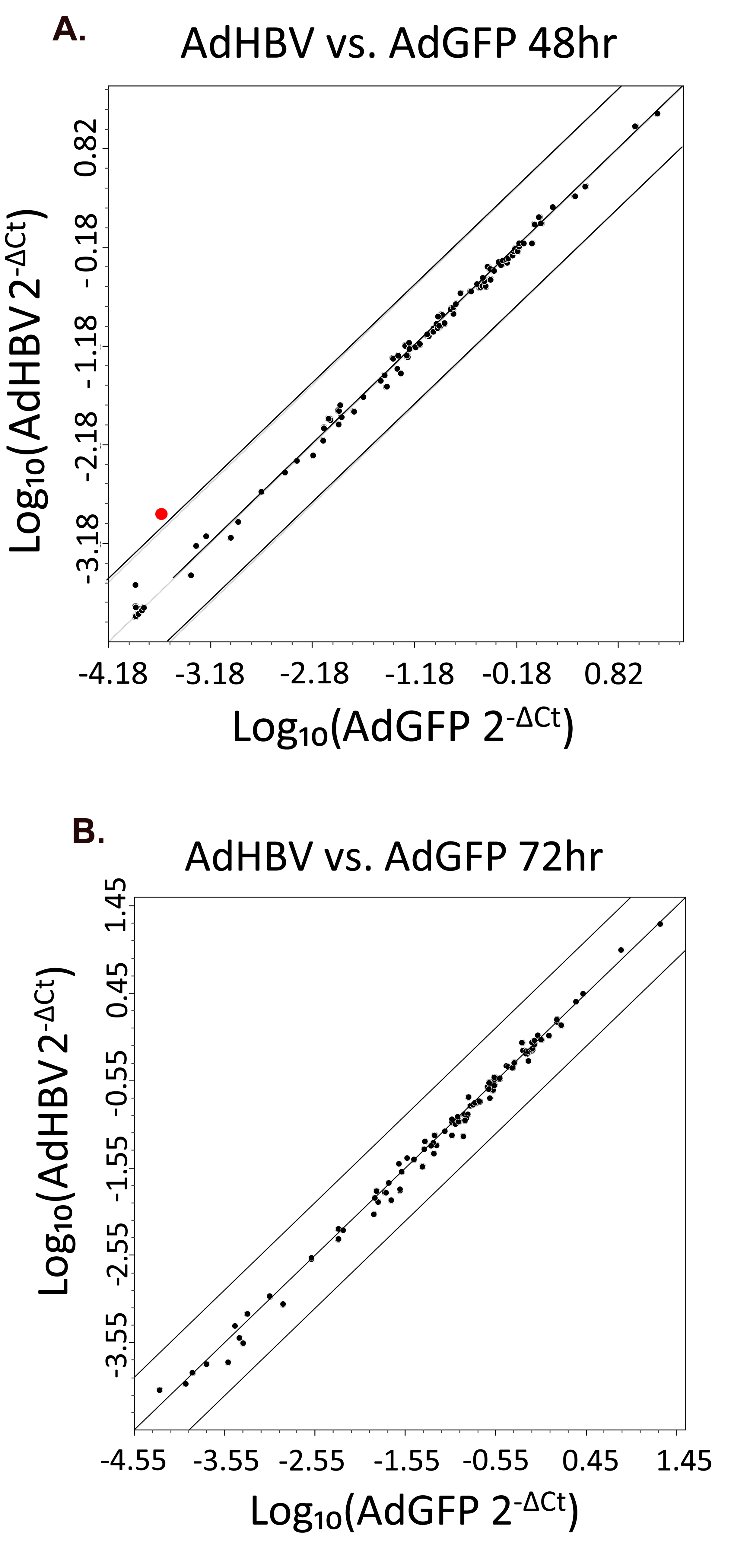

Supplement: S6 Fig — A-B. Expression of a panel of liver-enriched miRNAs was analyzed by qPCR array and plotted to visualize differential expression at 48hr (A) and 72hr (B) between AdGFP-infected and AdGFP-HBV-infected PRHs. Outer diagonal lines indicate a 2-fold change. (TIF) [file ppat.1005438.s006.tif]
