# Supplementary material for: Transcriptome-Wide Analysis of Hepatitis B Virus-Mediated Changes to Normal Hepatocyte Gene Expression
Source: PLoS Pathog. 2016 Feb 18;12(2):e1005438. doi: 10.1371/journal.ppat.1005438 (PMC4758756; doi:10.1371/journal.ppat.1005438)
Supplement: S1 Table — (DOCX) [file ppat.1005438.s007.docx]

S1 Table. Comparison of differential gene expression analysis pipelines.

|  | **STAR 🡺 DESeq2** | | **STAR 🡺 EdgeR** | | **BWA 🡺 DESeq2** | | **TopHat 🡺 Cufflinks** | |
| --- | --- | --- | --- | --- | --- | --- | --- | --- |
|  | **DEG** | **DEG ≥ 2-fold change** | **DEG** | **DEG ≥ 2-fold change** | **DEG** | **DEG ≥ 2-fold change** | **DEG** | **DEG ≥ 2-fold change** |
| **HBV 48hr to GFP 48hr** | 3323 | 115 | 2816 | 192 | 3255 | 133 | 2914 | 127 |
| **HBV 72hr to GFP 72hr** | 5070 | 512 | 4867 | 713 | 5159 | 571 | 4371 | 439 |
| **GFP 48hr to GFP 72hr** | 6973 | 1814 | 6904 | 2024 | 7122 | 1908 | 5575 | 1274 |
| **HBV 48hr to HBV 72hr** | 7447 | 1899 | 7240 | 2018 | 7519 | 1855 | 6067 | 1473 |
