# Supplementary material for: Transcriptome-Wide Analysis of Hepatitis B Virus-Mediated Changes to Normal Hepatocyte Gene Expression
Source: PLoS Pathog. 2016 Feb 18;12(2):e1005438. doi: 10.1371/journal.ppat.1005438 (PMC4758756; doi:10.1371/journal.ppat.1005438)
Supplement: S2 Table — (DOCX) [file ppat.1005438.s008.docx]

S2 Table. HBV-specific subset of differentially expressed genes.

|  |  | **RPKM** | | | | | | | | **Fold Change** | |
| --- | --- | --- | --- | --- | --- | --- | --- | --- | --- | --- | --- |
| **Gene** | **Ensembl ID** | **Uninf 0hr** | **Uninf 24hr** | **Uninf 48hr** | **Uninf 72hr** | **HBV 48hr** | **HBV 72hr** | **GFP 48hr** | **GFP 72hr** | **HBV to GFP 48h** | **HBV to GFP 72h** |
| Clec4m | ENSRNOG00000001017 | 0.00 | 0.33 | 0.76 | 0.47 | 2.34 | 2.81 | 0.98 | 0.63 | 2.34 | 4.41 |
| Fam107a | ENSRNOG00000033261 | 0.00 | 0.02 | 0.30 | 0.27 | 0.11 | 0.06 | 0.23 | 0.18 | -2.02 | -2.99 |
| Itgb8 | ENSRNOG00000006569 | 0.00 | 0.02 | 0.99 | 0.97 | 0.32 | 0.29 | 0.58 | 0.84 | -1.85 | -2.83 |
| Fut1 | ENSRNOG00000020995 | 0.05 | 0.36 | 0.16 | 0.20 | 0.57 | 0.79 | 0.20 | 0.29 | 2.79 | 2.71 |
| Serpina5 | ENSRNOG00000009855 | 0.54 | 0.18 | 0.31 | 0.55 | 0.20 | 0.15 | 0.35 | 0.41 | -1.71 | -2.61 |
| Lurap1 | ENSRNOG00000023459 | 0.27 | 0.69 | 1.19 | 1.06 | 0.52 | 0.41 | 0.90 | 1.02 | -1.73 | -2.47 |
| Galnt12 | ENSRNOG00000008099 | 0.02 | 0.35 | 1.84 | 2.80 | 1.30 | 1.06 | 1.99 | 2.45 | -1.56 | -2.28 |
| B3gnt9 | ENSRNOG00000015172 | 0.02 | 0.37 | 0.54 | 0.80 | 1.22 | 0.99 | 0.71 | 0.46 | 1.68 | 2.17 |
| Il5ra | ENSRNOG00000005954 | 0.00 | 0.21 | 0.76 | 0.42 | 1.99 | 1.59 | 1.11 | 0.76 | 1.75 | 2.09 |
| 42253.00 | ENSRNOG00000043182 | 0.04 | 0.21 | 2.60 | 3.29 | 1.61 | 1.40 | 2.51 | 2.93 | -1.59 | -2.06 |
| Lilra5 | ENSRNOG00000027808 | 0.10 | 0.05 | 2.57 | 1.52 | 0.67 | 0.59 | 1.56 | 1.22 | -2.32 | -2.03 |
| Krt20 | ENSRNOG00000027139 | 0.00 | 0.24 | 0.29 | 0.51 | 0.25 | 0.23 | 0.50 | 0.47 | -1.96 | -2.00 |
| Eps8 | ENSRNOG00000007047 | 0.02 | 0.21 | 0.39 | 0.24 | 0.65 | 0.56 | 0.41 | 0.28 | 1.55 | 1.99 |
| Zfp354b | ENSRNOG00000029678 | 0.32 | 1.16 | 1.22 | 0.83 | 2.04 | 2.32 | 1.48 | 1.22 | 1.35 | 1.91 |
| Btc | ENSRNOG00000002728 | 0.13 | 9.22 | 15.44 | 19.61 | 9.92 | 8.56 | 15.40 | 16.49 | -1.57 | -1.91 |
| Ccdc23 | ENSRNOG00000007404 | 1.42 | 8.37 | 6.98 | 9.71 | 4.86 | 4.09 | 6.37 | 7.88 | -1.33 | -1.90 |
| LOC100125368 | ENSRNOG00000043289 | 0.95 | 0.80 | 0.73 | 0.45 | 1.46 | 1.73 | 0.87 | 0.93 | 1.65 | 1.88 |
| Rdh5 | ENSRNOG00000040155 | 13.32 | 6.32 | 2.20 | 1.09 | 2.76 | 3.04 | 1.95 | 1.64 | 1.39 | 1.88 |
| Stxbp5l | ENSRNOG00000002496 | 0.38 | 0.52 | 0.31 | 0.17 | 0.50 | 0.54 | 0.31 | 0.29 | 1.60 | 1.87 |
| Cav1 | ENSRNOG00000006694 | 0.09 | 15.62 | 122.07 | 133.55 | 59.11 | 53.32 | 99.39 | 100.00 | -1.71 | -1.86 |
| Mafb | ENSRNOG00000016037 | 12.31 | 0.64 | 0.98 | 0.80 | 0.56 | 0.51 | 0.89 | 0.95 | -1.59 | -1.84 |
| Sytl4 | ENSRNOG00000003526 | 0.66 | 0.89 | 1.44 | 1.25 | 0.93 | 0.68 | 1.36 | 1.24 | -1.48 | -1.79 |
| Cd274 | ENSRNOG00000016112 | 1.29 | 0.24 | 0.53 | 0.52 | 1.51 | 1.59 | 0.60 | 0.90 | 2.44 | 1.77 |
| Tspan17 | ENSRNOG00000018122 | 0.01 | 5.78 | 14.70 | 18.60 | 9.72 | 8.32 | 14.62 | 14.70 | -1.52 | -1.75 |
| Slc1a3 | ENSRNOG00000016163 | 0.11 | 0.16 | 0.49 | 0.53 | 0.27 | 0.26 | 0.44 | 0.45 | -1.65 | -1.73 |
| Slc16a6 | ENSRNOG00000000245 | 0.89 | 2.42 | 2.40 | 2.19 | 5.18 | 5.17 | 3.12 | 3.06 | 1.64 | 1.70 |
| Gatsl2 | ENSRNOG00000001484 | 0.34 | 0.38 | 0.72 | 0.83 | 0.46 | 0.46 | 0.73 | 0.79 | -1.62 | -1.70 |
| Ccnjl | ENSRNOG00000003893 | 0.02 | 0.01 | 0.42 | 0.59 | 0.18 | 0.21 | 0.30 | 0.35 | -1.66 | -1.68 |
| Hspa4l | ENSRNOG00000010819 | 1.55 | 2.33 | 3.17 | 2.76 | 4.55 | 5.00 | 3.17 | 3.05 | 1.41 | 1.65 |
| Pomgnt2 | ENSRNOG00000019492 | 0.90 | 1.91 | 2.11 | 2.06 | 1.37 | 1.00 | 2.00 | 1.67 | -1.48 | -1.64 |
| Mir568 | ENSRNOG00000036329 | 4.70 | 22.44 | 21.02 | 24.50 | 15.13 | 11.60 | 21.36 | 19.22 | -1.43 | -1.63 |
| AABR06018038.2 | ENSRNOG00000028120 | 0.03 | 0.30 | 0.23 | 0.23 | 0.45 | 0.44 | 0.25 | 0.27 | 1.78 | 1.63 |
| Slc36a4 | ENSRNOG00000011455 | 0.14 | 0.70 | 1.92 | 1.83 | 1.27 | 1.17 | 1.77 | 1.89 | -1.41 | -1.60 |
| Lin52 | ENSRNOG00000043441 | 2.54 | 2.43 | 3.29 | 2.66 | 4.57 | 4.47 | 3.00 | 2.82 | 1.49 | 1.59 |
| Mtfr1 | ENSRNOG00000021359 | 6.42 | 6.09 | 8.35 | 7.99 | 12.38 | 13.66 | 9.04 | 8.68 | 1.35 | 1.59 |
| Chst14 | ENSRNOG00000045997 | 0.07 | 1.10 | 2.71 | 3.77 | 1.99 | 1.90 | 2.62 | 3.04 | -1.34 | -1.58 |
| Mybl1 | ENSRNOG00000021669 | 0.49 | 2.69 | 3.12 | 3.20 | 6.33 | 5.67 | 4.13 | 3.63 | 1.51 | 1.58 |
| Ccdc38 | ENSRNOG00000021892 | 1.45 | 1.23 | 1.10 | 1.02 | 0.74 | 0.57 | 1.12 | 0.90 | -1.52 | -1.57 |
| RGD1564541 | ENSRNOG00000012564 | 1.82 | 5.06 | 4.50 | 4.84 | 2.98 | 3.03 | 4.36 | 4.78 | -1.49 | -1.56 |
| Zfp358 | ENSRNOG00000000974 | 1.34 | 6.32 | 10.73 | 12.95 | 7.43 | 7.23 | 10.66 | 11.41 | -1.45 | -1.56 |
| Cflar | ENSRNOG00000012473 | 5.64 | 3.04 | 6.91 | 6.71 | 10.47 | 12.31 | 7.89 | 7.98 | 1.31 | 1.56 |
| Hes6 | ENSRNOG00000020194 | 47.99 | 16.13 | 19.71 | 21.92 | 13.87 | 12.50 | 18.19 | 19.64 | -1.33 | -1.55 |
| Snx21 | ENSRNOG00000015181 | 6.22 | 5.98 | 6.72 | 7.04 | 4.54 | 3.88 | 5.94 | 5.99 | -1.33 | -1.53 |
| Tceal1 | ENSRNOG00000002387 | 0.30 | 2.91 | 5.49 | 7.16 | 3.76 | 3.72 | 4.92 | 5.74 | -1.32 | -1.52 |
| Gjb3 | ENSRNOG00000014372 | 0.00 | 5.23 | 8.52 | 8.25 | 14.38 | 14.86 | 9.91 | 9.83 | 1.43 | 1.52 |
| Tgfbr1 | ENSRNOG00000007036 | 2.64 | 3.69 | 6.53 | 8.47 | 5.69 | 5.20 | 7.46 | 8.00 | -1.33 | -1.52 |
| Hdac8 | ENSRNOG00000003122 | 1.89 | 2.92 | 4.40 | 5.55 | 3.63 | 3.19 | 4.29 | 4.90 | -1.20 | -1.52 |
| Gdpd1 | ENSRNOG00000005596 | 4.34 | 5.37 | 12.09 | 12.71 | 8.23 | 7.50 | 11.11 | 11.50 | -1.37 | -1.52 |
| Ntpcr | ENSRNOG00000019922 | 14.80 | 12.85 | 9.01 | 11.46 | 7.21 | 6.47 | 8.57 | 9.90 | -1.21 | -1.52 |
| Nop58 | ENSRNOG00000016486 | 4.31 | 38.49 | 29.53 | 27.49 | 54.72 | 52.19 | 37.44 | 34.76 | 1.44 | 1.51 |
| AABR06068818.1 | ENSRNOG00000027116 | 0.21 | 21.73 | 9.36 | 9.14 | 6.55 | 5.31 | 9.49 | 8.10 | -1.47 | -1.51 |
| RGD1309821 | ENSRNOG00000023208 | 4.83 | 2.57 | 3.28 | 4.70 | 2.47 | 2.43 | 3.34 | 3.70 | -1.37 | -1.51 |
| Nuak2 | ENSRNOG00000000034 | 4.98 | 4.01 | 13.26 | 15.48 | 8.07 | 7.98 | 11.59 | 12.11 | -1.46 | -1.50 |
| Tmsb4x | ENSRNOG00000047931 | 142.12 | 1067.58 | 3756.25 | 3975.06 | 2633.68 | 2232.87 | 3256.04 | 3390.10 | -1.25 | -1.50 |
| Sec31b | ENSRNOG00000025781 | 0.34 | 0.68 | 0.56 | 0.73 | 0.32 | 0.36 | 0.51 | 0.55 | -1.63 | -1.50 |
| Iqcb1 | ENSRNOG00000038868 | 0.85 | 1.46 | 2.72 | 2.91 | 1.89 | 1.71 | 2.49 | 2.60 | -1.33 | -1.50 |
| Gmeb1 | ENSRNOG00000010910 | 0.71 | 2.47 | 3.41 | 3.29 | 5.09 | 5.25 | 4.19 | 3.56 | 1.20 | 1.49 |
| Lrrc16b | ENSRNOG00000025518 | 0.02 | 0.51 | 0.58 | 0.74 | 0.42 | 0.35 | 0.60 | 0.52 | -1.46 | -1.49 |
| Pak1ip1 | ENSRNOG00000023799 | 1.44 | 11.47 | 7.50 | 6.62 | 12.13 | 12.48 | 8.54 | 8.48 | 1.40 | 1.48 |
| Sh3bgr | ENSRNOG00000028238 | 0.03 | 2.00 | 14.29 | 16.20 | 8.14 | 9.33 | 11.94 | 13.97 | -1.49 | -1.48 |
| Ppp2r3b | ENSRNOG00000049270 | 0.55 | 2.28 | 3.41 | 3.63 | 5.98 | 6.14 | 3.87 | 4.18 | 1.52 | 1.48 |
| Abhd14b | ENSRNOG00000012073 | 113.52 | 47.87 | 16.12 | 22.28 | 13.26 | 11.79 | 16.89 | 17.61 | -1.29 | -1.48 |
| Actn2 | ENSRNOG00000017833 | 0.34 | 1.04 | 0.43 | 0.39 | 0.59 | 0.63 | 0.36 | 0.43 | 1.62 | 1.48 |
| LOC691909 | ENSRNOG00000028771 | 0.31 | 1.90 | 2.42 | 3.33 | 1.74 | 1.68 | 2.23 | 2.51 | -1.30 | -1.48 |
| LOC100363915 | ENSRNOG00000013520 | 2.19 | 28.65 | 28.22 | 22.88 | 55.38 | 51.12 | 34.38 | 34.89 | 1.59 | 1.48 |
| Arl6ip5 | ENSRNOG00000006818 | 12.20 | 114.41 | 110.05 | 127.05 | 81.05 | 76.99 | 106.03 | 114.59 | -1.33 | -1.47 |
| Marcksl1 | ENSRNOG00000009113 | 0.01 | 0.36 | 3.54 | 4.36 | 2.53 | 2.54 | 3.75 | 3.77 | -1.50 | -1.47 |
| Neil1 | ENSRNOG00000018577 | 2.59 | 4.88 | 4.53 | 5.61 | 3.07 | 3.17 | 4.20 | 4.69 | -1.39 | -1.46 |
| Larp1b | ENSRNOG00000038366 | 16.50 | 16.81 | 14.77 | 13.95 | 10.43 | 9.78 | 14.43 | 14.27 | -1.40 | -1.45 |
| Sdad1 | ENSRNOG00000022229 | 0.85 | 9.20 | 6.04 | 5.51 | 9.20 | 9.22 | 7.10 | 6.44 | 1.28 | 1.44 |
| Nap1l3 | ENSRNOG00000029087 | 0.19 | 1.84 | 1.81 | 1.92 | 1.20 | 1.11 | 1.73 | 1.63 | -1.46 | -1.44 |
| Srms | ENSRNOG00000013003 | 1.96 | 29.53 | 4.20 | 3.36 | 5.64 | 6.05 | 4.22 | 4.23 | 1.32 | 1.44 |
| Lrp3 | ENSRNOG00000011451 | 13.96 | 4.71 | 4.15 | 4.79 | 3.12 | 2.90 | 3.78 | 4.22 | -1.23 | -1.44 |
| Rmi1 | ENSRNOG00000019108 | 3.83 | 8.57 | 14.68 | 13.64 | 22.48 | 23.99 | 17.80 | 16.84 | 1.24 | 1.44 |
| Cep76 | ENSRNOG00000021918 | 0.85 | 2.20 | 4.03 | 3.28 | 7.34 | 6.22 | 4.91 | 4.36 | 1.47 | 1.44 |
| Kitlg | ENSRNOG00000005386 | 0.01 | 3.27 | 7.34 | 7.85 | 15.58 | 15.67 | 10.31 | 11.00 | 1.49 | 1.44 |
| Tspan5 | ENSRNOG00000015913 | 0.06 | 1.97 | 3.50 | 4.73 | 3.02 | 2.89 | 4.08 | 4.19 | -1.37 | -1.44 |
| Fam25a | ENSRNOG00000010401 | 0.04 | 1.73 | 83.74 | 90.08 | 45.35 | 51.22 | 63.68 | 74.16 | -1.42 | -1.43 |
| Cdc42se1 | ENSRNOG00000021112 | 7.84 | 28.71 | 41.65 | 35.21 | 54.61 | 57.87 | 45.88 | 40.76 | 1.17 | 1.43 |
| Nek3 | ENSRNOG00000012757 | 2.23 | 4.11 | 3.26 | 3.02 | 2.35 | 2.00 | 2.99 | 2.90 | -1.29 | -1.43 |
| Rasl10a | ENSRNOG00000008951 | 0.29 | 2.57 | 5.10 | 8.47 | 3.92 | 4.67 | 5.50 | 6.75 | -1.42 | -1.43 |
| Gstz1 | ENSRNOG00000047708 | 175.89 | 44.06 | 12.98 | 15.91 | 8.88 | 8.43 | 10.94 | 12.16 | -1.25 | -1.43 |
| Ago1 | ENSRNOG00000027320 | 1.04 | 2.51 | 5.41 | 4.57 | 3.45 | 3.09 | 5.15 | 4.44 | -1.52 | -1.43 |
| Ttll4 | ENSRNOG00000017129 | 1.08 | 4.46 | 5.76 | 4.25 | 8.47 | 7.41 | 6.44 | 5.24 | 1.29 | 1.42 |
| Rhpn1 | ENSRNOG00000007597 | 0.05 | 2.11 | 3.93 | 3.77 | 2.15 | 2.41 | 3.30 | 3.47 | -1.56 | -1.42 |
| Ccdc64 | ENSRNOG00000001145 | 0.27 | 1.70 | 3.17 | 2.84 | 5.49 | 5.55 | 3.64 | 3.94 | 1.48 | 1.42 |
| Nradd | ENSRNOG00000020936 | 0.05 | 0.47 | 23.93 | 30.25 | 15.51 | 17.46 | 21.37 | 25.02 | -1.40 | -1.42 |
| Dhtkd1 | ENSRNOG00000023587 | 70.49 | 4.29 | 3.13 | 2.84 | 1.99 | 1.58 | 2.60 | 2.26 | -1.33 | -1.42 |
| AABR06058720.1 | ENSRNOG00000014180 | 1.40 | 2.27 | 1.60 | 2.25 | 1.14 | 1.19 | 1.58 | 1.70 | -1.41 | -1.42 |
| Ahnak2 | ENSRNOG00000028545 | 0.00 | 1.03 | 1.96 | 2.01 | 1.51 | 1.38 | 2.25 | 1.98 | -1.51 | -1.41 |
| Gpsm1 | ENSRNOG00000018666 | 0.21 | 2.02 | 5.44 | 6.37 | 4.42 | 4.19 | 5.61 | 5.98 | -1.29 | -1.41 |
| Trmt61a | ENSRNOG00000011398 | 0.64 | 11.92 | 6.56 | 5.93 | 8.98 | 8.95 | 6.94 | 6.39 | 1.27 | 1.41 |
| Vasn | ENSRNOG00000004141 | 3.07 | 6.50 | 12.81 | 15.03 | 9.15 | 9.24 | 12.58 | 13.18 | -1.40 | -1.41 |
| Cnnm2 | ENSRNOG00000020113 | 1.16 | 1.43 | 1.77 | 2.00 | 1.53 | 1.26 | 1.87 | 1.81 | -1.24 | -1.41 |
| Mthfr | ENSRNOG00000008553 | 1.36 | 3.42 | 4.05 | 3.36 | 7.55 | 6.07 | 4.86 | 4.34 | 1.53 | 1.41 |
| Trak2 | ENSRNOG00000010881 | 2.53 | 22.87 | 119.62 | 117.60 | 82.73 | 76.27 | 116.01 | 107.69 | -1.42 | -1.40 |
| Sec24a | ENSRNOG00000004563 | 6.97 | 5.49 | 6.06 | 4.65 | 8.30 | 8.19 | 6.59 | 5.93 | 1.24 | 1.39 |
| Bloc1s6 | ENSRNOG00000037160 | 3.49 | 11.22 | 13.22 | 14.44 | 10.12 | 9.23 | 12.62 | 12.97 | -1.27 | -1.39 |
| Akap17b | ENSRNOG00000009746 | 0.47 | 1.03 | 1.20 | 1.22 | 0.79 | 0.80 | 1.29 | 1.12 | -1.66 | -1.39 |
| Nrcam | ENSRNOG00000004067 | 0.16 | 0.93 | 1.34 | 1.04 | 1.69 | 1.63 | 1.34 | 1.19 | 1.24 | 1.39 |
| Slc12a6 | ENSRNOG00000005196 | 0.28 | 0.87 | 1.95 | 2.30 | 1.41 | 1.45 | 1.84 | 2.03 | -1.32 | -1.38 |
| Nbeal2 | ENSRNOG00000027880 | 0.59 | 1.78 | 2.10 | 1.77 | 3.17 | 3.15 | 2.56 | 2.30 | 1.22 | 1.38 |
| Gar1 | ENSRNOG00000009877 | 1.33 | 16.58 | 8.55 | 9.02 | 13.30 | 13.74 | 9.43 | 10.02 | 1.39 | 1.38 |
| Rhob | ENSRNOG00000021403 | 21.51 | 54.38 | 54.11 | 61.58 | 42.00 | 42.38 | 54.37 | 59.19 | -1.31 | -1.38 |
| Rn50_X_0564.1 | ENSRNOG00000051191 | 0.37 | 2.45 | 3.30 | 4.13 | 2.18 | 2.56 | 3.13 | 3.57 | -1.46 | -1.38 |
| Unc93b1 | ENSRNOG00000017703 | 1.01 | 6.62 | 8.93 | 10.14 | 6.98 | 6.78 | 8.56 | 9.46 | -1.24 | -1.38 |
| AABR06047726.1 | ENSRNOG00000032182 | 0.75 | 1.84 | 2.08 | 1.86 | 2.96 | 3.46 | 2.19 | 2.52 | 1.33 | 1.38 |
| Rhpn2 | ENSRNOG00000011885 | 2.22 | 3.75 | 4.95 | 4.33 | 8.23 | 8.15 | 6.19 | 5.96 | 1.31 | 1.38 |
| Acbd4 | ENSRNOG00000003108 | 6.67 | 7.19 | 5.78 | 6.86 | 4.49 | 4.27 | 5.29 | 5.93 | -1.19 | -1.38 |
| Mpzl1 | ENSRNOG00000003248 | 0.14 | 0.63 | 1.35 | 1.61 | 1.09 | 1.00 | 1.44 | 1.39 | -1.33 | -1.38 |
| Serf1 | ENSRNOG00000017945 | 5.53 | 17.89 | 19.26 | 24.67 | 15.03 | 15.15 | 18.27 | 21.03 | -1.23 | -1.38 |
| Zfp90 | ENSRNOG00000020087 | 0.19 | 2.13 | 2.34 | 2.73 | 1.99 | 1.82 | 2.50 | 2.52 | -1.28 | -1.37 |
| Inpp5b | ENSRNOG00000048506 | 0.68 | 0.57 | 0.84 | 0.86 | 1.26 | 1.44 | 0.92 | 1.06 | 1.35 | 1.37 |
| Pard6b | ENSRNOG00000010883 | 0.88 | 3.84 | 4.07 | 4.48 | 3.61 | 3.21 | 4.94 | 4.42 | -1.39 | -1.36 |
| Ptprv | ENSRNOG00000005277 | 0.00 | 0.13 | 0.80 | 0.92 | 0.64 | 0.63 | 0.93 | 0.87 | -1.47 | -1.36 |
| Mblac2 | ENSRNOG00000016252 | 1.85 | 3.32 | 5.55 | 5.13 | 4.30 | 3.77 | 5.86 | 5.16 | -1.38 | -1.36 |
| Rrp9 | ENSRNOG00000012927 | 1.24 | 13.06 | 9.23 | 9.80 | 12.75 | 14.13 | 10.02 | 10.50 | 1.25 | 1.36 |
| Nol9 | ENSRNOG00000010109 | 1.38 | 10.72 | 9.75 | 8.80 | 13.31 | 13.65 | 11.09 | 10.15 | 1.18 | 1.36 |
| Nup153 | ENSRNOG00000001456 | 2.88 | 8.04 | 11.00 | 10.15 | 17.70 | 17.53 | 13.01 | 13.05 | 1.34 | 1.35 |
| Chuk | ENSRNOG00000022485 | 13.70 | 13.81 | 12.79 | 10.97 | 17.38 | 17.57 | 13.95 | 13.09 | 1.23 | 1.35 |
| Pknox1 | ENSRNOG00000001184 | 1.59 | 2.51 | 4.24 | 3.24 | 4.72 | 4.58 | 3.81 | 3.41 | 1.22 | 1.35 |
| P4ha1 | ENSRNOG00000050655 | 3.17 | 7.12 | 10.15 | 10.21 | 15.41 | 16.20 | 11.95 | 12.10 | 1.27 | 1.35 |
| Tmx3 | ENSRNOG00000045740 | 3.66 | 9.61 | 18.26 | 16.96 | 23.39 | 24.51 | 19.75 | 18.32 | 1.17 | 1.35 |
| Sidt2 | ENSRNOG00000017871 | 18.74 | 20.68 | 29.10 | 29.91 | 20.06 | 19.36 | 28.08 | 26.36 | -1.42 | -1.35 |
| R3hdm4 | ENSRNOG00000011489 | 14.52 | 22.78 | 22.12 | 25.55 | 17.84 | 16.27 | 21.59 | 22.15 | -1.23 | -1.35 |
| Ptbp2 | ENSRNOG00000010827 | 0.88 | 3.58 | 5.08 | 6.46 | 4.57 | 4.41 | 5.54 | 6.01 | -1.23 | -1.35 |
| Tmem59 | ENSRNOG00000009778 | 87.11 | 80.41 | 120.98 | 128.17 | 96.46 | 86.96 | 110.90 | 117.98 | -1.17 | -1.34 |
| Tsr1 | ENSRNOG00000002980 | 3.71 | 16.69 | 14.12 | 12.52 | 21.88 | 21.41 | 16.98 | 16.10 | 1.27 | 1.34 |
| Dph1 | ENSRNOG00000003116 | 2.11 | 17.16 | 9.05 | 8.77 | 11.81 | 12.32 | 10.00 | 9.27 | 1.16 | 1.34 |
| Zc3h10 | ENSRNOG00000040281 | 0.71 | 1.91 | 2.49 | 3.40 | 2.25 | 2.30 | 2.77 | 3.11 | -1.25 | -1.34 |
| Hs6st1 | ENSRNOG00000014516 | 2.68 | 10.44 | 17.72 | 17.89 | 12.29 | 12.46 | 15.95 | 16.83 | -1.32 | -1.34 |
| Mxd4 | ENSRNOG00000015033 | 20.24 | 52.55 | 89.11 | 103.85 | 70.70 | 67.84 | 87.94 | 91.59 | -1.26 | -1.34 |
| Paics | ENSRNOG00000002101 | 5.51 | 11.65 | 13.76 | 11.53 | 18.59 | 18.24 | 14.47 | 13.77 | 1.27 | 1.34 |
| Fam20a | ENSRNOG00000003969 | 9.54 | 11.30 | 7.19 | 10.55 | 6.49 | 6.53 | 7.88 | 8.78 | -1.23 | -1.33 |
| Dexi | ENSRNOG00000002635 | 13.78 | 11.62 | 13.65 | 15.65 | 10.64 | 10.74 | 13.04 | 14.41 | -1.24 | -1.33 |
| Myo1d | ENSRNOG00000003276 | 5.88 | 7.19 | 13.88 | 15.04 | 10.62 | 10.69 | 12.74 | 14.32 | -1.22 | -1.33 |
| Zfp36l1 | ENSRNOG00000030024 | 119.74 | 73.37 | 79.72 | 84.92 | 62.10 | 60.51 | 80.33 | 81.12 | -1.31 | -1.33 |
| Srebf1 | ENSRNOG00000003463 | 101.95 | 21.53 | 7.79 | 6.73 | 10.15 | 9.69 | 8.02 | 7.37 | 1.25 | 1.33 |
| Mettl16 | ENSRNOG00000002764 | 1.59 | 6.86 | 6.23 | 6.40 | 9.14 | 10.00 | 7.03 | 7.62 | 1.28 | 1.33 |
| Galnt11 | ENSRNOG00000008117 | 9.54 | 5.64 | 7.63 | 8.62 | 6.24 | 5.63 | 7.70 | 7.52 | -1.25 | -1.32 |
| AABR06026181.1 | ENSRNOG00000025804 | 1.86 | 4.60 | 3.18 | 2.50 | 4.41 | 4.42 | 3.42 | 3.36 | 1.27 | 1.32 |
| Ttc21b | ENSRNOG00000005868 | 1.28 | 2.70 | 5.29 | 6.01 | 4.38 | 4.11 | 5.08 | 5.48 | -1.18 | -1.32 |
| Gfer | ENSRNOG00000013370 | 9.53 | 16.15 | 10.87 | 9.83 | 15.73 | 14.71 | 12.01 | 11.24 | 1.29 | 1.32 |
| Mesdc1 | ENSRNOG00000012349 | 0.36 | 2.67 | 4.57 | 5.11 | 7.36 | 8.32 | 5.62 | 6.36 | 1.29 | 1.32 |
| RGD1559896 | ENSRNOG00000018836 | 2.21 | 7.11 | 14.09 | 15.15 | 10.43 | 9.53 | 13.34 | 12.69 | -1.30 | -1.32 |
| Sh3bgrl | ENSRNOG00000002280 | 29.15 | 29.09 | 141.70 | 150.65 | 112.43 | 103.49 | 132.18 | 137.64 | -1.19 | -1.32 |
| Abcd3 | ENSRNOG00000011929 | 39.46 | 24.69 | 22.57 | 25.13 | 19.31 | 18.21 | 22.69 | 24.22 | -1.19 | -1.32 |
| Mid1ip1 | ENSRNOG00000003228 | 21.70 | 26.88 | 15.88 | 18.02 | 24.31 | 23.72 | 17.77 | 18.19 | 1.35 | 1.32 |
| Palld | ENSRNOG00000010107 | 4.13 | 22.58 | 73.21 | 64.09 | 43.27 | 44.56 | 67.36 | 59.07 | -1.58 | -1.31 |
| Fam110b | ENSRNOG00000009114 | 0.55 | 2.57 | 9.38 | 7.98 | 6.99 | 6.09 | 8.55 | 8.06 | -1.24 | -1.31 |
| Azin2 | ENSRNOG00000042650 | 0.15 | 2.94 | 12.92 | 12.31 | 9.71 | 8.12 | 11.42 | 10.75 | -1.19 | -1.31 |
| Ndufb10 | ENSRNOG00000014568 | 53.78 | 69.40 | 44.48 | 50.75 | 37.95 | 32.24 | 46.19 | 42.58 | -1.23 | -1.31 |
| Rrs1 | ENSRNOG00000007240 | 3.86 | 14.86 | 9.43 | 9.45 | 13.42 | 12.97 | 10.75 | 10.01 | 1.23 | 1.31 |
| Lamc1 | ENSRNOG00000002680 | 2.31 | 20.61 | 60.70 | 74.73 | 56.05 | 54.89 | 65.43 | 72.50 | -1.18 | -1.31 |
| Prdx2 | ENSRNOG00000003520 | 40.38 | 106.46 | 151.95 | 183.16 | 132.54 | 122.69 | 150.39 | 161.90 | -1.15 | -1.31 |
| Eif2s1 | ENSRNOG00000009432 | 12.35 | 67.95 | 56.17 | 52.09 | 78.15 | 82.21 | 63.57 | 63.55 | 1.21 | 1.31 |
| Sdr9c7 | ENSRNOG00000004459 | 17.36 | 11.05 | 9.13 | 7.86 | 11.19 | 10.61 | 9.08 | 8.20 | 1.21 | 1.30 |
| Riok2 | ENSRNOG00000012692 | 2.30 | 13.15 | 10.73 | 9.58 | 13.02 | 13.66 | 10.85 | 10.57 | 1.18 | 1.30 |
| Pgs1 | ENSRNOG00000002949 | 1.67 | 9.17 | 8.52 | 7.65 | 11.47 | 10.88 | 9.49 | 8.43 | 1.19 | 1.30 |
| Orai1 | ENSRNOG00000001336 | 10.82 | 25.40 | 38.33 | 43.32 | 25.96 | 27.60 | 36.92 | 36.27 | -1.44 | -1.30 |
| Ttll7 | ENSRNOG00000031997 | 0.28 | 2.72 | 6.72 | 6.42 | 9.11 | 9.79 | 7.72 | 7.60 | 1.16 | 1.30 |
| Rfxap | ENSRNOG00000028382 | 2.82 | 8.14 | 6.45 | 8.11 | 5.48 | 5.87 | 7.06 | 7.71 | -1.31 | -1.30 |
| Haus8 | ENSRNOG00000023534 | 0.79 | 5.85 | 6.96 | 6.32 | 10.28 | 9.03 | 7.76 | 7.01 | 1.31 | 1.30 |
| Ppp1r14a | ENSRNOG00000020676 | 9.21 | 31.47 | 41.18 | 46.32 | 25.60 | 29.43 | 34.16 | 38.60 | -1.35 | -1.30 |
| LOC691931 | ENSRNOG00000010875 | 3.35 | 5.03 | 7.51 | 6.56 | 10.09 | 11.06 | 7.94 | 8.60 | 1.25 | 1.30 |
| Rnf126 | ENSRNOG00000009028 | 5.03 | 9.06 | 9.89 | 9.22 | 13.66 | 14.06 | 11.37 | 10.95 | 1.18 | 1.30 |
| Nf2 | ENSRNOG00000007948 | 2.30 | 5.95 | 10.31 | 10.52 | 7.64 | 7.46 | 9.95 | 9.76 | -1.32 | -1.30 |
| Rbm15 | ENSRNOG00000047499 | 1.79 | 3.17 | 3.15 | 3.06 | 4.91 | 4.32 | 3.75 | 3.36 | 1.29 | 1.30 |
| Lrp8 | ENSRNOG00000013064 | 0.02 | 1.44 | 1.47 | 1.58 | 2.47 | 2.63 | 1.76 | 2.05 | 1.38 | 1.29 |
| Suco | ENSRNOG00000026542 | 2.75 | 2.63 | 5.61 | 5.36 | 7.52 | 7.64 | 5.96 | 5.96 | 1.24 | 1.29 |
| Alkbh8 | ENSRNOG00000024525 | 0.81 | 1.48 | 1.58 | 1.48 | 2.10 | 2.11 | 1.60 | 1.65 | 1.29 | 1.29 |
| Scamp1 | ENSRNOG00000010774 | 6.24 | 10.27 | 12.10 | 13.77 | 9.84 | 9.73 | 11.66 | 12.65 | -1.20 | -1.29 |
| Csrnp1 | ENSRNOG00000033433 | 2.97 | 4.34 | 5.58 | 5.50 | 4.79 | 4.22 | 5.91 | 5.49 | -1.25 | -1.29 |
| RGD1307315 | ENSRNOG00000013690 | 0.15 | 2.95 | 3.20 | 3.38 | 2.29 | 2.25 | 3.02 | 2.92 | -1.34 | -1.29 |
| Ssna1 | ENSRNOG00000011093 | 27.08 | 37.55 | 27.85 | 26.44 | 35.55 | 35.51 | 26.94 | 27.86 | 1.30 | 1.29 |
| Gna11 | ENSRNOG00000005446 | 4.45 | 8.96 | 12.55 | 13.15 | 18.65 | 19.57 | 14.05 | 15.36 | 1.31 | 1.29 |
| Galt | ENSRNOG00000014766 | 14.12 | 10.33 | 10.58 | 12.49 | 9.55 | 8.22 | 10.97 | 10.66 | -1.17 | -1.28 |
| Urb1 | ENSRNOG00000002080 | 0.83 | 3.34 | 3.46 | 3.54 | 5.12 | 4.94 | 3.87 | 3.89 | 1.30 | 1.28 |
| Helb | ENSRNOG00000004189 | 0.72 | 1.73 | 2.88 | 2.83 | 3.89 | 3.80 | 3.22 | 2.99 | 1.19 | 1.28 |
| Cdk10 | ENSRNOG00000016088 | 7.15 | 27.67 | 24.03 | 21.75 | 32.00 | 33.90 | 28.00 | 26.73 | 1.13 | 1.28 |
| Nudt9 | ENSRNOG00000002186 | 9.88 | 35.06 | 18.66 | 18.57 | 23.59 | 25.79 | 20.12 | 20.35 | 1.16 | 1.28 |
| Hnrnpf | ENSRNOG00000014562 | 15.06 | 47.42 | 48.98 | 44.92 | 69.92 | 65.59 | 54.59 | 51.83 | 1.26 | 1.28 |
| Twistnb | ENSRNOG00000010750 | 1.93 | 7.03 | 7.01 | 7.10 | 9.59 | 10.04 | 7.88 | 7.94 | 1.20 | 1.28 |
| Alg12 | ENSRNOG00000004591 | 2.86 | 10.11 | 5.70 | 5.64 | 7.27 | 7.73 | 5.97 | 6.12 | 1.20 | 1.28 |
| Atf2 | ENSRNOG00000001597 | 5.78 | 6.41 | 10.97 | 10.77 | 14.93 | 15.44 | 12.04 | 12.23 | 1.22 | 1.27 |
| Cnpy2 | ENSRNOG00000003549 | 40.28 | 54.86 | 61.70 | 69.60 | 49.71 | 46.77 | 56.69 | 60.08 | -1.16 | -1.27 |
| Ocrl | ENSRNOG00000003875 | 1.23 | 2.89 | 3.75 | 3.73 | 2.97 | 2.59 | 3.53 | 3.33 | -1.21 | -1.27 |
| Mybbp1a | ENSRNOG00000015236 | 4.87 | 28.83 | 23.07 | 21.58 | 28.95 | 29.92 | 25.35 | 23.76 | 1.13 | 1.27 |
| Scap | ENSRNOG00000020853 | 8.42 | 12.42 | 13.74 | 15.62 | 12.78 | 11.50 | 14.20 | 14.75 | -1.13 | -1.27 |
| Desi2 | ENSRNOG00000004524 | 3.66 | 10.70 | 10.29 | 10.21 | 14.71 | 14.10 | 10.82 | 11.20 | 1.34 | 1.27 |
| Ttc3 | ENSRNOG00000001682 | 1.93 | 6.35 | 15.47 | 15.59 | 12.32 | 11.15 | 15.97 | 14.27 | -1.32 | -1.27 |
| Chd6 | ENSRNOG00000016744 | 0.90 | 1.20 | 2.20 | 2.82 | 1.94 | 2.00 | 2.30 | 2.55 | -1.20 | -1.26 |
| Fam120b | ENSRNOG00000001487 | 3.01 | 3.75 | 4.25 | 5.03 | 3.67 | 3.41 | 4.39 | 4.34 | -1.21 | -1.26 |
| Spast | ENSRNOG00000027136 | 2.01 | 4.06 | 4.92 | 5.33 | 4.25 | 3.78 | 5.04 | 4.81 | -1.20 | -1.26 |
| Btaf1 | ENSRNOG00000017938 | 2.14 | 4.13 | 4.86 | 4.50 | 6.51 | 6.87 | 5.25 | 5.50 | 1.22 | 1.26 |
| Tomm40 | ENSRNOG00000018556 | 7.05 | 36.20 | 28.20 | 24.10 | 37.45 | 34.22 | 30.73 | 27.40 | 1.20 | 1.26 |
| Nudt18 | ENSRNOG00000011831 | 5.15 | 24.51 | 18.70 | 21.89 | 14.32 | 14.42 | 17.25 | 18.31 | -1.22 | -1.26 |
| Atp6v0a1 | ENSRNOG00000036814 | 8.42 | 9.33 | 7.56 | 7.69 | 5.24 | 5.52 | 7.17 | 7.01 | -1.39 | -1.26 |
| Thumpd3 | ENSRNOG00000006941 | 3.61 | 14.61 | 11.95 | 11.62 | 14.42 | 14.96 | 12.67 | 11.99 | 1.12 | 1.26 |
| Phka1 | ENSRNOG00000003063 | 0.57 | 1.14 | 1.82 | 2.02 | 1.52 | 1.36 | 1.80 | 1.72 | -1.20 | -1.26 |
| Ccdc181 | ENSRNOG00000002860 | 0.59 | 8.20 | 8.09 | 8.40 | 6.46 | 5.69 | 7.62 | 7.23 | -1.20 | -1.26 |
| Zmym3 | ENSRNOG00000003707 | 2.33 | 4.99 | 5.59 | 7.05 | 4.74 | 4.83 | 5.56 | 6.12 | -1.19 | -1.25 |
| Ndfip1 | ENSRNOG00000013562 | 68.90 | 112.61 | 116.75 | 130.33 | 97.35 | 93.68 | 114.47 | 118.32 | -1.19 | -1.25 |
| Rabac1 | ENSRNOG00000020233 | 37.13 | 80.26 | 82.60 | 82.82 | 65.80 | 55.70 | 77.00 | 70.39 | -1.19 | -1.25 |
| Capn1 | ENSRNOG00000020935 | 2.64 | 13.52 | 17.15 | 16.76 | 13.62 | 12.66 | 16.95 | 15.99 | -1.26 | -1.25 |
| Ppp4r1 | ENSRNOG00000013733 | 3.66 | 12.41 | 17.62 | 17.52 | 23.22 | 24.23 | 19.39 | 19.54 | 1.18 | 1.25 |
| Taf1d | ENSRNOG00000010921 | 2.48 | 23.81 | 14.70 | 14.78 | 19.58 | 20.95 | 16.15 | 16.90 | 1.19 | 1.25 |
| Gpatch4 | ENSRNOG00000018969 | 0.50 | 6.73 | 4.69 | 4.45 | 6.77 | 5.99 | 5.26 | 4.84 | 1.27 | 1.25 |
| Ddx56 | ENSRNOG00000004670 | 4.89 | 21.38 | 12.85 | 12.33 | 17.06 | 15.81 | 14.03 | 12.77 | 1.20 | 1.25 |
| Wdr5 | ENSRNOG00000008212 | 3.06 | 15.83 | 12.81 | 14.16 | 17.72 | 18.04 | 14.54 | 14.58 | 1.20 | 1.25 |
| Chmp7 | ENSRNOG00000016939 | 5.78 | 11.44 | 15.17 | 14.32 | 11.66 | 11.65 | 15.04 | 14.66 | -1.31 | -1.25 |
| Rad52 | ENSRNOG00000009742 | 2.98 | 12.52 | 9.70 | 10.71 | 7.80 | 7.14 | 9.37 | 9.00 | -1.22 | -1.25 |
| Hdac7 | ENSRNOG00000008308 | 0.06 | 1.94 | 10.86 | 11.73 | 8.70 | 8.78 | 11.11 | 11.07 | -1.29 | -1.25 |
| Shisa5 | ENSRNOG00000020667 | 5.48 | 9.92 | 12.69 | 14.53 | 10.54 | 10.59 | 12.45 | 13.32 | -1.20 | -1.25 |
| Rabepk | ENSRNOG00000018591 | 1.64 | 5.01 | 4.74 | 5.58 | 4.30 | 3.89 | 5.13 | 4.89 | -1.21 | -1.25 |
| Cactin | ENSRNOG00000039852 | 1.71 | 6.20 | 5.80 | 5.55 | 7.66 | 7.56 | 6.61 | 6.12 | 1.14 | 1.25 |
| Ppp1r13b | ENSRNOG00000012653 | 1.08 | 2.25 | 3.94 | 4.33 | 3.07 | 3.34 | 3.80 | 4.20 | -1.26 | -1.24 |
| Pomk | ENSRNOG00000014628 | 3.12 | 11.02 | 10.96 | 12.19 | 9.35 | 9.11 | 10.80 | 11.42 | -1.17 | -1.24 |
| Ttc12 | ENSRNOG00000008595 | 0.15 | 1.93 | 5.39 | 6.68 | 4.07 | 4.63 | 5.02 | 5.80 | -1.25 | -1.24 |
| Uso1 | ENSRNOG00000002301 | 28.53 | 37.07 | 35.81 | 35.37 | 43.51 | 45.35 | 39.31 | 36.87 | 1.09 | 1.24 |
| Txndc15 | ENSRNOG00000000133 | 23.35 | 35.80 | 37.52 | 41.92 | 32.01 | 28.97 | 35.61 | 36.22 | -1.13 | -1.24 |
| Fzd6 | ENSRNOG00000004660 | 0.30 | 2.16 | 5.48 | 5.96 | 4.83 | 4.79 | 5.66 | 5.98 | -1.19 | -1.24 |
| Gabarapl2 | ENSRNOG00000019425 | 21.07 | 16.38 | 19.57 | 21.53 | 14.99 | 15.84 | 18.44 | 19.77 | -1.25 | -1.24 |
| Rbm28 | ENSRNOG00000005468 | 2.05 | 7.44 | 8.32 | 7.71 | 10.28 | 10.61 | 8.83 | 8.66 | 1.15 | 1.24 |
| Ncl | ENSRNOG00000018273 | 22.83 | 91.77 | 86.93 | 78.79 | 114.27 | 111.97 | 95.47 | 91.51 | 1.18 | 1.23 |
| Cyp4f6 | ENSRNOG00000034157 | 33.90 | 26.02 | 25.87 | 29.75 | 21.22 | 20.55 | 25.25 | 25.60 | -1.21 | -1.23 |
| Trim5 | ENSRNOG00000017191 | 13.57 | 18.72 | 17.23 | 17.89 | 13.80 | 13.53 | 17.05 | 16.86 | -1.25 | -1.23 |
| Men1 | ENSRNOG00000021054 | 3.82 | 7.11 | 8.93 | 9.79 | 11.12 | 11.81 | 9.35 | 9.66 | 1.17 | 1.23 |
| Rabggtb | ENSRNOG00000009992 | 23.18 | 52.39 | 36.68 | 38.24 | 49.05 | 47.46 | 41.00 | 38.83 | 1.18 | 1.23 |
| Nit1 | ENSRNOG00000003881 | 59.56 | 89.01 | 92.77 | 92.52 | 75.21 | 68.92 | 86.41 | 85.73 | -1.17 | -1.23 |
| Tekt1 | ENSRNOG00000014973 | 0.00 | 0.96 | 4.51 | 4.86 | 3.37 | 3.72 | 4.04 | 4.63 | -1.22 | -1.23 |
| Tmem19 | ENSRNOG00000003985 | 13.24 | 40.28 | 32.61 | 27.80 | 22.40 | 20.42 | 28.13 | 25.37 | -1.27 | -1.23 |
| Hps6 | ENSRNOG00000018433 | 0.35 | 2.54 | 3.02 | 3.88 | 2.56 | 2.75 | 2.97 | 3.41 | -1.18 | -1.23 |
| Camsap3 | ENSRNOG00000000986 | 1.67 | 5.02 | 4.26 | 4.60 | 3.63 | 3.55 | 4.18 | 4.41 | -1.17 | -1.23 |
| Arf6 | ENSRNOG00000004791 | 14.86 | 50.27 | 53.94 | 56.49 | 74.47 | 76.62 | 60.48 | 62.88 | 1.21 | 1.23 |
| Cluh | ENSRNOG00000002669 | 33.72 | 29.11 | 21.38 | 19.15 | 26.63 | 28.07 | 22.36 | 23.06 | 1.17 | 1.23 |
| Snapin | ENSRNOG00000013356 | 15.75 | 34.35 | 37.30 | 39.22 | 29.58 | 27.34 | 35.18 | 33.89 | -1.21 | -1.23 |
| Ndst2 | ENSRNOG00000027171 | 3.42 | 3.26 | 3.66 | 4.70 | 3.66 | 3.52 | 4.23 | 4.36 | -1.17 | -1.23 |
| Ppcdc | ENSRNOG00000018711 | 1.26 | 2.42 | 1.80 | 1.78 | 2.55 | 2.21 | 1.89 | 1.82 | 1.33 | 1.23 |
| Cul2 | ENSRNOG00000015292 | 9.93 | 13.94 | 12.51 | 11.99 | 15.83 | 16.17 | 13.66 | 13.33 | 1.14 | 1.22 |
| Usp43 | ENSRNOG00000003785 | 0.00 | 0.52 | 2.29 | 2.47 | 2.63 | 2.55 | 2.08 | 2.10 | 1.25 | 1.22 |
| Pigx | ENSRNOG00000033623 | 5.31 | 30.29 | 37.67 | 40.48 | 30.27 | 30.14 | 36.00 | 37.21 | -1.21 | -1.22 |
| Exoc2 | ENSRNOG00000033643 | 2.88 | 7.22 | 10.11 | 11.25 | 8.32 | 8.20 | 9.99 | 10.11 | -1.22 | -1.22 |
| Faf2 | ENSRNOG00000017607 | 9.58 | 17.22 | 18.20 | 16.17 | 21.49 | 22.76 | 18.01 | 18.81 | 1.18 | 1.22 |
| Cd81 | ENSRNOG00000020451 | 292.14 | 992.03 | 1166.54 | 1179.09 | 934.52 | 887.53 | 1085.89 | 1092.31 | -1.18 | -1.22 |
| Tp53bp2 | ENSRNOG00000003237 | 3.39 | 4.15 | 6.62 | 7.16 | 5.83 | 5.86 | 7.07 | 7.21 | -1.23 | -1.22 |
| Armc9 | ENSRNOG00000025418 | 0.66 | 2.91 | 4.32 | 4.42 | 3.76 | 3.31 | 4.29 | 4.07 | -1.16 | -1.22 |
| Tle2 | ENSRNOG00000005874 | 2.31 | 4.41 | 5.58 | 6.53 | 4.65 | 4.44 | 5.52 | 5.46 | -1.20 | -1.22 |
| Ppp1r2 | ENSRNOG00000001733 | 14.08 | 35.96 | 88.89 | 81.17 | 134.16 | 120.37 | 107.20 | 99.75 | 1.23 | 1.22 |
| Timm9 | ENSRNOG00000008222 | 9.28 | 24.10 | 10.67 | 12.86 | 15.88 | 16.12 | 11.62 | 13.36 | 1.35 | 1.22 |
| Gabpa | ENSRNOG00000001549 | 3.41 | 6.40 | 7.96 | 7.15 | 10.12 | 9.61 | 8.72 | 7.97 | 1.14 | 1.22 |
| Rwdd4 | ENSRNOG00000022500 | 8.49 | 14.18 | 14.20 | 12.34 | 16.81 | 16.57 | 14.19 | 13.75 | 1.17 | 1.22 |
| Smpdl3b | ENSRNOG00000042326 | 0.28 | 2.75 | 4.56 | 4.09 | 3.44 | 3.21 | 4.01 | 3.94 | -1.18 | -1.22 |
| Tp53 | ENSRNOG00000010756 | 8.74 | 57.37 | 58.12 | 55.74 | 74.08 | 73.13 | 64.82 | 60.72 | 1.13 | 1.22 |
| Mfap1a | ENSRNOG00000015984 | 3.61 | 6.91 | 8.27 | 7.58 | 10.76 | 10.87 | 8.82 | 9.02 | 1.20 | 1.21 |
| Ptpra | ENSRNOG00000021223 | 3.21 | 8.56 | 14.69 | 16.00 | 13.10 | 12.48 | 14.28 | 15.29 | -1.11 | -1.21 |
| Myl12a | ENSRNOG00000015278 | 18.85 | 415.50 | 663.53 | 669.87 | 554.89 | 506.25 | 637.90 | 619.99 | -1.17 | -1.21 |
| Ift81 | ENSRNOG00000001294 | 0.82 | 6.10 | 7.46 | 8.76 | 6.32 | 6.66 | 7.64 | 8.15 | -1.23 | -1.21 |
| Smg9 | ENSRNOG00000019596 | 1.45 | 7.39 | 5.29 | 5.95 | 7.54 | 7.94 | 5.94 | 6.61 | 1.25 | 1.21 |
| Zfp275 | ENSRNOG00000018419 | 0.12 | 1.11 | 3.72 | 3.54 | 3.21 | 2.77 | 3.81 | 3.38 | -1.21 | -1.21 |
| Dkc1 | ENSRNOG00000048151 | 8.91 | 37.60 | 28.06 | 30.22 | 38.01 | 37.92 | 31.28 | 31.60 | 1.20 | 1.21 |
| Me3 | ENSRNOG00000017311 | 5.07 | 3.62 | 4.57 | 4.43 | 3.67 | 3.08 | 4.39 | 3.77 | -1.21 | -1.21 |
| Acadvl | ENSRNOG00000018114 | 84.82 | 56.56 | 34.14 | 36.25 | 27.74 | 28.09 | 31.98 | 34.28 | -1.17 | -1.21 |
| Rbpms | ENSRNOG00000013328 | 22.24 | 147.18 | 141.43 | 184.65 | 123.77 | 130.84 | 149.83 | 159.67 | -1.23 | -1.21 |
| RGD1302996 | ENSRNOG00000000812 | 5.26 | 11.70 | 8.10 | 7.92 | 7.44 | 6.41 | 8.52 | 7.82 | -1.16 | -1.21 |
| Pelo | ENSRNOG00000023127 | 5.17 | 20.98 | 18.81 | 19.70 | 26.67 | 25.33 | 21.09 | 21.17 | 1.25 | 1.21 |
| Pwp2 | ENSRNOG00000001210 | 1.02 | 6.31 | 6.23 | 5.88 | 8.60 | 7.81 | 6.85 | 6.54 | 1.24 | 1.21 |
| Ltv1 | ENSRNOG00000015217 | 3.92 | 14.26 | 13.61 | 13.18 | 16.28 | 16.28 | 14.31 | 13.61 | 1.12 | 1.21 |
| Gtf2f2 | ENSRNOG00000029316 | 2.97 | 10.41 | 5.52 | 6.12 | 8.33 | 8.21 | 6.31 | 6.88 | 1.30 | 1.20 |
| Hsbp1 | ENSRNOG00000014415 | 50.13 | 101.68 | 100.98 | 109.35 | 89.95 | 79.97 | 100.86 | 97.17 | -1.14 | -1.20 |
| Sp110 | ENSRNOG00000033747 | 3.14 | 11.80 | 12.05 | 12.96 | 10.10 | 9.59 | 12.05 | 11.65 | -1.21 | -1.20 |
| Mpv17l2 | ENSRNOG00000019394 | 5.32 | 19.82 | 12.54 | 11.26 | 16.47 | 14.84 | 13.29 | 12.46 | 1.22 | 1.20 |
| Pno1 | ENSRNOG00000005524 | 3.97 | 31.66 | 23.21 | 22.61 | 29.59 | 29.47 | 25.06 | 24.74 | 1.16 | 1.20 |
| Akap8l | ENSRNOG00000006355 | 17.02 | 14.52 | 13.59 | 18.25 | 11.94 | 13.36 | 14.84 | 16.21 | -1.26 | -1.20 |
| Hmgcr | ENSRNOG00000016122 | 14.46 | 17.15 | 14.72 | 13.52 | 19.30 | 18.50 | 16.84 | 15.54 | 1.13 | 1.20 |
| Commd3 | ENSRNOG00000016383 | 32.98 | 77.07 | 90.87 | 93.49 | 82.10 | 73.66 | 90.04 | 89.28 | -1.11 | -1.20 |
| Elk4 | ENSRNOG00000007887 | 2.03 | 6.61 | 8.26 | 8.66 | 6.37 | 6.90 | 8.45 | 8.36 | -1.35 | -1.20 |
| Rnps1 | ENSRNOG00000008703 | 6.37 | 29.18 | 34.60 | 35.64 | 46.69 | 47.20 | 39.19 | 39.68 | 1.17 | 1.20 |
| Cdkn2aip | ENSRNOG00000022736 | 3.71 | 7.52 | 11.87 | 13.14 | 15.51 | 16.17 | 12.97 | 13.60 | 1.18 | 1.20 |
| Mavs | ENSRNOG00000025295 | 6.55 | 4.70 | 4.47 | 5.17 | 4.02 | 3.91 | 4.64 | 4.73 | -1.17 | -1.20 |
| Hccs | ENSRNOG00000025910 | 4.54 | 8.08 | 6.37 | 6.62 | 7.81 | 8.17 | 6.87 | 6.87 | 1.12 | 1.20 |
| Pyurfl1 | ENSRNOG00000006858 | 10.10 | 23.10 | 15.21 | 15.84 | 11.95 | 11.02 | 14.28 | 13.32 | -1.21 | -1.20 |
| Aldh2 | ENSRNOG00000001344 | 529.18 | 281.09 | 150.96 | 166.03 | 136.22 | 128.71 | 151.53 | 155.50 | -1.13 | -1.20 |
| Eml5 | ENSRNOG00000004207 | 1.10 | 1.22 | 1.61 | 1.76 | 1.38 | 1.55 | 1.69 | 1.87 | -1.24 | -1.20 |
| LOC364561 | ENSRNOG00000020474 | 10.62 | 48.82 | 44.71 | 41.51 | 58.82 | 50.74 | 49.10 | 42.79 | 1.18 | 1.20 |
| Zmynd19 | ENSRNOG00000007752 | 1.09 | 4.30 | 5.46 | 5.50 | 7.81 | 6.98 | 5.71 | 5.89 | 1.35 | 1.20 |
| Tmcc1 | ENSRNOG00000011614 | 5.64 | 5.11 | 7.41 | 7.24 | 9.65 | 9.99 | 7.95 | 8.43 | 1.20 | 1.20 |
| Abtb2 | ENSRNOG00000008510 | 7.83 | 8.19 | 7.33 | 7.17 | 8.29 | 8.52 | 7.24 | 7.19 | 1.13 | 1.19 |
| Wdr45b | ENSRNOG00000036662 | 6.11 | 23.14 | 26.78 | 23.10 | 30.71 | 29.12 | 26.90 | 24.59 | 1.12 | 1.19 |
| Wdr41 | ENSRNOG00000025462 | 2.44 | 9.35 | 10.25 | 11.02 | 9.31 | 8.58 | 10.29 | 10.31 | -1.12 | -1.19 |
| Ano10 | ENSRNOG00000000219 | 9.03 | 15.82 | 20.57 | 22.04 | 16.69 | 17.60 | 19.67 | 21.14 | -1.20 | -1.19 |
| Lonp2 | ENSRNOG00000015162 | 114.86 | 43.85 | 46.18 | 49.65 | 39.99 | 38.45 | 45.00 | 46.18 | -1.14 | -1.19 |
| Bnip3l | ENSRNOG00000009820 | 11.12 | 31.11 | 47.62 | 52.47 | 38.42 | 39.40 | 46.81 | 47.32 | -1.24 | -1.19 |
| ND1 | ENSRNOG00000030644 | 6897.28 | 6680.83 | 4622.12 | 4927.95 | 3915.33 | 3613.46 | 4879.75 | 4336.95 | -1.26 | -1.19 |
| Uba2 | ENSRNOG00000021113 | 2.01 | 5.20 | 6.71 | 6.57 | 9.53 | 9.24 | 7.72 | 7.85 | 1.22 | 1.19 |
| Tnpo3 | ENSRNOG00000021758 | 3.63 | 12.26 | 15.38 | 14.25 | 19.35 | 19.14 | 16.52 | 16.26 | 1.15 | 1.19 |
| Anapc16 | ENSRNOG00000000576 | 3.35 | 13.26 | 11.34 | 13.57 | 15.60 | 15.36 | 11.88 | 13.04 | 1.29 | 1.19 |
| Prpf31 | ENSRNOG00000013504 | 2.83 | 10.60 | 9.45 | 8.99 | 11.66 | 11.27 | 10.04 | 9.58 | 1.15 | 1.19 |
| Ogfod3 | ENSRNOG00000036668 | 3.32 | 6.68 | 10.02 | 10.84 | 8.90 | 8.54 | 10.31 | 10.21 | -1.18 | -1.19 |
| Tfip11 | ENSRNOG00000000663 | 3.59 | 12.16 | 8.94 | 8.91 | 10.99 | 11.58 | 9.22 | 9.86 | 1.18 | 1.19 |
| Smek1 | ENSRNOG00000027773 | 7.06 | 9.13 | 13.59 | 14.40 | 18.78 | 19.35 | 15.43 | 16.48 | 1.20 | 1.19 |
| Yy1 | ENSRNOG00000004339 | 7.53 | 15.47 | 18.09 | 18.49 | 24.78 | 24.73 | 20.32 | 21.09 | 1.20 | 1.18 |
| Dmap1 | ENSRNOG00000019407 | 4.07 | 14.36 | 9.19 | 9.80 | 7.37 | 7.59 | 8.96 | 9.05 | -1.23 | -1.18 |
| Plscr1 | ENSRNOG00000008048 | 0.06 | 3.03 | 14.16 | 13.05 | 10.17 | 9.68 | 12.39 | 11.55 | -1.24 | -1.18 |
| Hp1bp3 | ENSRNOG00000014445 | 35.48 | 42.48 | 71.78 | 82.85 | 65.98 | 64.90 | 73.54 | 77.29 | -1.13 | -1.18 |
| Gba | ENSRNOG00000049281 | 6.53 | 18.51 | 31.68 | 31.83 | 27.35 | 25.27 | 30.52 | 30.08 | -1.13 | -1.18 |
| Ube2r2 | ENSRNOG00000010727 | 22.13 | 40.72 | 57.97 | 64.84 | 49.68 | 48.46 | 61.00 | 57.65 | -1.25 | -1.18 |
| Zfp259 | ENSRNOG00000018481 | 5.91 | 29.37 | 17.61 | 17.42 | 22.28 | 21.76 | 20.06 | 18.64 | 1.09 | 1.18 |
| Gata6 | ENSRNOG00000023433 | 1.34 | 3.17 | 7.33 | 8.75 | 6.76 | 7.20 | 7.83 | 8.55 | -1.18 | -1.18 |
| Fam63a | ENSRNOG00000021131 | 7.13 | 15.50 | 26.55 | 26.05 | 22.29 | 20.87 | 26.73 | 24.81 | -1.22 | -1.18 |
| Tmed7 | ENSRNOG00000003691 | 33.60 | 28.94 | 28.89 | 28.97 | 33.67 | 34.58 | 30.15 | 29.67 | 1.10 | 1.18 |
| Slc35d2 | ENSRNOG00000027229 | 8.16 | 26.60 | 19.43 | 20.25 | 16.75 | 15.64 | 19.27 | 18.57 | -1.17 | -1.18 |
| Zmiz1 | ENSRNOG00000010488 | 1.05 | 4.23 | 7.07 | 7.73 | 5.97 | 5.94 | 7.49 | 7.04 | -1.27 | -1.17 |
| Edem1 | ENSRNOG00000007944 | 35.96 | 20.22 | 25.15 | 28.44 | 34.19 | 35.81 | 27.54 | 30.80 | 1.22 | 1.17 |
| Ddx18 | ENSRNOG00000025430 | 3.38 | 14.91 | 11.90 | 11.56 | 15.28 | 14.85 | 13.65 | 12.76 | 1.10 | 1.17 |
| Hebp2 | ENSRNOG00000011409 | 0.16 | 10.13 | 14.50 | 14.72 | 11.16 | 10.91 | 13.57 | 12.89 | -1.23 | -1.17 |
| Traf4 | ENSRNOG00000013169 | 5.49 | 17.92 | 25.69 | 26.79 | 33.37 | 31.34 | 29.44 | 27.01 | 1.12 | 1.17 |
| Wapal | ENSRNOG00000039630 | 10.09 | 14.27 | 19.37 | 18.74 | 24.44 | 26.41 | 20.73 | 22.76 | 1.16 | 1.17 |
| Gatad2a | ENSRNOG00000022173 | 6.80 | 18.43 | 18.40 | 17.99 | 23.88 | 22.29 | 20.76 | 19.24 | 1.13 | 1.17 |
| Itm2b | ENSRNOG00000016271 | 268.05 | 219.27 | 377.23 | 390.16 | 289.13 | 295.35 | 346.00 | 348.28 | -1.21 | -1.17 |
| Zfp180 | ENSRNOG00000029336 | 1.41 | 3.10 | 5.19 | 5.89 | 7.58 | 7.50 | 6.13 | 6.48 | 1.22 | 1.17 |
| Morc3 | ENSRNOG00000026236 | 2.88 | 9.97 | 11.02 | 11.02 | 15.19 | 14.37 | 13.05 | 12.43 | 1.15 | 1.17 |
| Calu | ENSRNOG00000006197 | 23.31 | 44.72 | 95.00 | 98.82 | 115.81 | 116.14 | 100.78 | 100.48 | 1.13 | 1.17 |
| Plcg1 | ENSRNOG00000016340 | 4.76 | 12.46 | 34.55 | 37.54 | 32.49 | 31.22 | 36.34 | 36.73 | -1.14 | -1.17 |
| Hcfc2 | ENSRNOG00000011072 | 1.34 | 3.33 | 3.28 | 3.31 | 4.37 | 3.97 | 3.58 | 3.44 | 1.20 | 1.17 |
| Auh | ENSRNOG00000011684 | 27.83 | 27.31 | 31.45 | 29.03 | 26.32 | 24.02 | 29.12 | 28.24 | -1.12 | -1.17 |
| Cd164 | ENSRNOG00000000304 | 356.24 | 140.79 | 122.03 | 145.72 | 116.78 | 113.39 | 128.42 | 133.31 | -1.12 | -1.16 |
| Dapk2 | ENSRNOG00000017332 | 3.83 | 27.50 | 53.82 | 54.23 | 48.57 | 43.81 | 55.62 | 51.49 | -1.16 | -1.16 |
| Anapc4 | ENSRNOG00000004130 | 3.89 | 17.45 | 18.93 | 20.42 | 17.56 | 16.15 | 19.27 | 18.98 | -1.11 | -1.16 |
| Qsox2 | ENSRNOG00000018574 | 0.84 | 3.28 | 4.09 | 4.25 | 5.46 | 5.76 | 4.64 | 5.00 | 1.16 | 1.16 |
| Mt-nd4 | ENSRNOG00000029707 | 6788.65 | 6674.73 | 4282.14 | 5036.77 | 3767.42 | 3894.39 | 4592.70 | 4570.88 | -1.24 | -1.16 |
| Kxd1 | ENSRNOG00000019971 | 15.21 | 26.01 | 18.59 | 18.24 | 22.92 | 23.61 | 19.65 | 20.50 | 1.15 | 1.16 |
| Hnrnpul2 | ENSRNOG00000019507 | 11.86 | 25.05 | 37.31 | 33.73 | 45.60 | 46.23 | 40.19 | 40.12 | 1.12 | 1.16 |
| Cdk2ap2 | ENSRNOG00000018391 | 43.07 | 36.83 | 25.10 | 30.01 | 35.19 | 32.62 | 26.51 | 28.34 | 1.31 | 1.16 |
| Ublcp1 | ENSRNOG00000004477 | 3.90 | 15.11 | 15.48 | 16.35 | 13.35 | 13.48 | 15.57 | 15.79 | -1.18 | -1.16 |
| Strap | ENSRNOG00000007134 | 14.11 | 48.91 | 34.33 | 34.00 | 43.50 | 39.20 | 36.58 | 34.09 | 1.17 | 1.16 |
| Dnajc11 | ENSRNOG00000008802 | 6.80 | 19.14 | 14.97 | 15.16 | 18.95 | 19.76 | 16.37 | 17.20 | 1.14 | 1.16 |
| St3gal4 | ENSRNOG00000009850 | 2.57 | 5.76 | 16.09 | 15.20 | 23.32 | 21.59 | 19.94 | 18.79 | 1.15 | 1.16 |
| Polr1b | ENSRNOG00000018349 | 1.28 | 5.16 | 3.34 | 2.91 | 4.27 | 3.81 | 3.55 | 3.32 | 1.18 | 1.16 |
| Morc2 | ENSRNOG00000019624 | 1.07 | 3.43 | 5.95 | 6.26 | 7.36 | 7.74 | 6.55 | 6.74 | 1.11 | 1.16 |
| Samd4a | ENSRNOG00000010489 | 1.76 | 16.93 | 12.66 | 12.51 | 11.07 | 10.63 | 12.46 | 12.42 | -1.14 | -1.16 |
| Ccdc53 | ENSRNOG00000005102 | 15.98 | 29.38 | 24.40 | 27.56 | 19.69 | 20.78 | 22.39 | 24.27 | -1.15 | -1.16 |
| Gyg1 | ENSRNOG00000011146 | 3.08 | 19.58 | 24.93 | 26.56 | 21.29 | 21.23 | 24.72 | 24.79 | -1.18 | -1.16 |
| Pde12 | ENSRNOG00000013264 | 1.90 | 6.91 | 5.44 | 5.17 | 7.41 | 7.09 | 6.14 | 6.19 | 1.19 | 1.16 |
| Pkn3 | ENSRNOG00000025892 | 1.74 | 7.73 | 8.79 | 8.38 | 11.21 | 11.04 | 9.74 | 9.66 | 1.13 | 1.15 |
| Cnp | ENSRNOG00000017496 | 2.23 | 24.53 | 26.93 | 28.30 | 24.01 | 23.34 | 25.98 | 27.16 | -1.10 | -1.15 |
| Tbc1d9b | ENSRNOG00000003050 | 3.97 | 12.36 | 13.16 | 12.89 | 12.02 | 10.87 | 13.27 | 12.65 | -1.12 | -1.15 |
| Rpia | ENSRNOG00000005576 | 1.26 | 5.35 | 7.21 | 7.59 | 10.29 | 9.62 | 8.78 | 8.41 | 1.15 | 1.15 |
| Fundc1 | ENSRNOG00000003470 | 3.56 | 8.34 | 9.68 | 9.90 | 7.70 | 7.48 | 9.18 | 8.69 | -1.21 | -1.15 |
| Kif1c | ENSRNOG00000031364 | 13.95 | 68.90 | 101.82 | 108.38 | 96.00 | 91.81 | 108.88 | 106.65 | -1.15 | -1.15 |
| Inpp5k | ENSRNOG00000004041 | 3.14 | 9.94 | 11.37 | 13.78 | 10.56 | 10.43 | 11.83 | 12.11 | -1.14 | -1.15 |
| Hnrnpk | ENSRNOG00000019113 | 58.19 | 119.23 | 169.68 | 158.98 | 200.41 | 202.80 | 181.10 | 177.95 | 1.09 | 1.15 |
| Eif4e | ENSRNOG00000013183 | 8.66 | 25.68 | 27.32 | 26.96 | 32.02 | 33.32 | 28.66 | 29.23 | 1.10 | 1.15 |
| Baz1b | ENSRNOG00000001453 | 3.22 | 4.76 | 9.09 | 8.80 | 12.94 | 11.54 | 10.52 | 10.13 | 1.21 | 1.15 |
| Mak16 | ENSRNOG00000010783 | 2.24 | 8.90 | 10.58 | 10.49 | 14.05 | 14.00 | 12.44 | 12.29 | 1.11 | 1.15 |
| Med6 | ENSRNOG00000006976 | 4.74 | 15.90 | 14.84 | 15.46 | 17.86 | 18.64 | 14.89 | 16.36 | 1.18 | 1.15 |
| Gmcl1 | ENSRNOG00000017838 | 4.06 | 7.07 | 10.88 | 11.46 | 9.45 | 9.11 | 10.81 | 10.56 | -1.16 | -1.15 |
| Pld2 | ENSRNOG00000019604 | 0.07 | 5.33 | 7.21 | 7.68 | 6.71 | 6.24 | 7.68 | 7.23 | -1.16 | -1.15 |
| Dnajc22 | ENSRNOG00000015837 | 42.18 | 39.11 | 24.70 | 25.20 | 29.32 | 28.47 | 25.99 | 25.06 | 1.11 | 1.15 |
| Bcl3 | ENSRNOG00000043416 | 4.26 | 18.93 | 13.88 | 14.67 | 21.50 | 19.38 | 17.07 | 17.07 | 1.24 | 1.15 |
| Tmem168 | ENSRNOG00000006690 | 7.00 | 12.47 | 11.54 | 11.56 | 13.73 | 14.59 | 11.99 | 12.84 | 1.13 | 1.15 |
| Zfp637 | ENSRNOG00000023065 | 4.10 | 20.15 | 19.09 | 19.10 | 14.56 | 14.73 | 17.65 | 17.00 | -1.23 | -1.14 |
| Galk2 | ENSRNOG00000009289 | 5.40 | 11.96 | 18.03 | 17.60 | 15.50 | 15.14 | 17.27 | 17.46 | -1.13 | -1.14 |
| Ilf3 | ENSRNOG00000022741 | 2.67 | 8.55 | 12.40 | 12.61 | 15.92 | 16.09 | 13.61 | 14.21 | 1.15 | 1.14 |
| Enoph1 | ENSRNOG00000002262 | 3.23 | 13.30 | 14.31 | 14.86 | 17.45 | 17.19 | 15.37 | 15.19 | 1.12 | 1.14 |
| Trappc8 | ENSRNOG00000022202 | 6.15 | 6.41 | 7.98 | 8.05 | 9.55 | 10.10 | 8.04 | 8.93 | 1.17 | 1.14 |
| Pkn1 | ENSRNOG00000004131 | 3.41 | 12.36 | 15.31 | 14.46 | 12.92 | 11.65 | 14.51 | 13.42 | -1.14 | -1.14 |
| Acsl1 | ENSRNOG00000010633 | 528.56 | 33.37 | 47.23 | 36.29 | 33.92 | 30.83 | 39.19 | 35.44 | -1.17 | -1.14 |
| Nup54 | ENSRNOG00000002247 | 2.49 | 9.15 | 10.09 | 10.36 | 13.27 | 12.55 | 11.13 | 11.13 | 1.18 | 1.14 |
| Rab11fip1 | ENSRNOG00000045531 | 0.70 | 5.95 | 6.37 | 5.86 | 7.17 | 6.93 | 6.34 | 6.15 | 1.11 | 1.14 |
| Mt-nd5 | ENSRNOG00000029971 | 1855.56 | 2029.42 | 1557.65 | 1728.24 | 1490.29 | 1420.67 | 1686.48 | 1628.34 | -1.15 | -1.14 |
| Eif4a3 | ENSRNOG00000045791 | 14.04 | 41.50 | 46.44 | 45.93 | 56.31 | 56.23 | 48.91 | 50.01 | 1.13 | 1.13 |
| Syncrip | ENSRNOG00000000204 | 6.76 | 18.85 | 24.55 | 22.27 | 30.37 | 27.95 | 26.43 | 24.86 | 1.13 | 1.13 |
| Eps15l1 | ENSRNOG00000013502 | 1.90 | 6.30 | 10.69 | 11.19 | 9.81 | 9.78 | 11.19 | 11.18 | -1.16 | -1.13 |
| Grpel2 | ENSRNOG00000042682 | 2.56 | 4.20 | 6.96 | 6.74 | 9.37 | 9.20 | 7.90 | 8.21 | 1.17 | 1.13 |
| Ankh | ENSRNOG00000010960 | 22.21 | 15.16 | 23.12 | 21.85 | 18.07 | 18.24 | 21.31 | 20.81 | -1.20 | -1.13 |
| Ryk | ENSRNOG00000008593 | 6.83 | 22.05 | 28.61 | 29.51 | 26.67 | 26.33 | 28.82 | 29.97 | -1.10 | -1.13 |
| Sdr39u1 | ENSRNOG00000020544 | 11.95 | 12.56 | 11.27 | 14.02 | 10.67 | 10.84 | 12.12 | 12.33 | -1.15 | -1.13 |
| Adprhl2 | ENSRNOG00000010849 | 4.97 | 24.43 | 22.34 | 24.04 | 28.83 | 28.56 | 23.58 | 25.59 | 1.20 | 1.13 |
| Fkbp1a | ENSRNOG00000008822 | 41.64 | 240.14 | 283.60 | 283.67 | 255.57 | 256.34 | 280.83 | 291.43 | -1.12 | -1.13 |
| Gramd1a | ENSRNOG00000021106 | 1.96 | 4.91 | 10.54 | 11.21 | 10.22 | 9.73 | 11.42 | 11.05 | -1.13 | -1.12 |
| Smarcd1 | ENSRNOG00000034268 | 0.72 | 3.74 | 6.12 | 6.97 | 5.38 | 5.50 | 6.57 | 6.24 | -1.24 | -1.12 |
| Stau1 | ENSRNOG00000007781 | 15.99 | 22.33 | 23.01 | 25.36 | 21.53 | 21.38 | 23.33 | 24.25 | -1.10 | -1.12 |
| Ufm1 | ENSRNOG00000038176 | 12.41 | 20.65 | 13.41 | 14.08 | 15.72 | 15.81 | 13.80 | 14.22 | 1.12 | 1.12 |
| LOC100365902 | ENSRNOG00000025383 | 12.60 | 22.45 | 32.72 | 33.07 | 29.61 | 27.78 | 32.92 | 31.42 | -1.13 | -1.12 |
| Pex1 | ENSRNOG00000025991 | 9.06 | 5.70 | 6.61 | 6.91 | 5.92 | 5.84 | 6.42 | 6.60 | -1.10 | -1.12 |
| Cpox | ENSRNOG00000001654 | 20.43 | 24.83 | 17.72 | 16.71 | 20.27 | 19.73 | 18.32 | 17.79 | 1.09 | 1.12 |
| Lrpprc | ENSRNOG00000005877 | 19.49 | 22.56 | 19.53 | 17.03 | 23.37 | 21.55 | 20.68 | 19.44 | 1.11 | 1.12 |
| Psmd5 | ENSRNOG00000018809 | 11.89 | 44.38 | 25.21 | 27.69 | 34.02 | 32.63 | 28.19 | 29.52 | 1.19 | 1.11 |
| Tmem127 | ENSRNOG00000022727 | 3.82 | 8.83 | 13.27 | 14.27 | 11.48 | 11.94 | 13.30 | 13.42 | -1.18 | -1.11 |
| Ftsj3 | ENSRNOG00000009857 | 2.85 | 25.92 | 16.23 | 16.25 | 20.81 | 19.46 | 18.42 | 17.64 | 1.11 | 1.11 |
| Fbxo11 | ENSRNOG00000016396 | 2.50 | 7.07 | 12.04 | 13.51 | 10.63 | 10.95 | 12.85 | 12.29 | -1.23 | -1.11 |
| Cfl2 | ENSRNOG00000045892 | 12.53 | 55.35 | 81.34 | 76.17 | 69.28 | 66.03 | 79.10 | 74.14 | -1.16 | -1.11 |
| Armc1 | ENSRNOG00000013253 | 8.70 | 7.42 | 6.89 | 8.03 | 9.38 | 8.90 | 7.86 | 8.07 | 1.18 | 1.11 |
| Mgat2 | ENSRNOG00000004234 | 9.31 | 14.60 | 14.78 | 16.36 | 19.58 | 18.62 | 16.09 | 16.90 | 1.20 | 1.11 |
| Rchy1 | ENSRNOG00000002546 | 6.45 | 14.23 | 13.68 | 13.51 | 15.71 | 14.36 | 13.21 | 13.04 | 1.17 | 1.11 |
| Stxbp3 | ENSRNOG00000020392 | 7.21 | 14.05 | 16.65 | 17.41 | 14.67 | 15.26 | 16.65 | 17.01 | -1.15 | -1.10 |
| Gnai3 | ENSRNOG00000019465 | 28.53 | 77.21 | 63.89 | 65.82 | 75.12 | 77.40 | 68.69 | 70.72 | 1.08 | 1.10 |
| RGD1566359 | ENSRNOG00000047934 | 3.76 | 11.82 | 17.75 | 18.03 | 16.25 | 16.52 | 18.34 | 18.40 | -1.15 | -1.10 |
| Dennd5a | ENSRNOG00000012206 | 3.02 | 15.53 | 27.32 | 29.03 | 25.10 | 24.96 | 28.03 | 27.79 | -1.13 | -1.10 |
| Msantd4 | ENSRNOG00000022245 | 6.67 | 12.14 | 11.88 | 14.09 | 11.02 | 11.61 | 12.09 | 12.91 | -1.11 | -1.10 |
| Sav1 | ENSRNOG00000005264 | 2.72 | 7.98 | 10.39 | 11.59 | 10.02 | 10.65 | 11.17 | 11.85 | -1.13 | -1.10 |
| Ncstn | ENSRNOG00000005355 | 13.19 | 21.13 | 29.40 | 32.44 | 28.13 | 28.95 | 30.38 | 32.18 | -1.10 | -1.10 |
| Brd7 | ENSRNOG00000014419 | 12.04 | 26.02 | 29.24 | 29.04 | 35.20 | 34.57 | 31.94 | 31.68 | 1.09 | 1.10 |
| Zdhhc16 | ENSRNOG00000046530 | 3.80 | 13.32 | 14.77 | 16.56 | 13.49 | 13.66 | 14.63 | 15.16 | -1.10 | -1.10 |
| Kifap3 | ENSRNOG00000002544 | 2.36 | 11.07 | 15.02 | 16.79 | 13.11 | 13.56 | 14.86 | 15.05 | -1.15 | -1.10 |
| Micu2 | ENSRNOG00000011168 | 10.01 | 28.54 | 29.99 | 30.97 | 27.90 | 27.09 | 29.82 | 29.78 | -1.08 | -1.09 |
| Os9 | ENSRNOG00000025570 | 53.47 | 49.05 | 70.46 | 67.12 | 60.33 | 58.39 | 67.55 | 63.41 | -1.14 | -1.08 |
