# Supplementary material for: Transcriptome-Wide Analysis of Hepatitis B Virus-Mediated Changes to Normal Hepatocyte Gene Expression
Source: PLoS Pathog. 2016 Feb 18;12(2):e1005438. doi: 10.1371/journal.ppat.1005438 (PMC4758756; doi:10.1371/journal.ppat.1005438)
Supplement: S3 Table — (DOCX) [file ppat.1005438.s009.docx]

S3 Table. Secondary dataset RNA-seq overview.

| **Sample** | **Total Reads** | **Mapped** | **% Mapped** | **Uniquely Mapped** | **% Unique Mapped** | **% Unmapped** | **Multi-mapping Reads** | **% Multiple Mapping** | **Total Mappings of Reads** |
| --- | --- | --- | --- | --- | --- | --- | --- | --- | --- |
| gfp_48_1 | 62,035,925 | 54,699,531 | 88.17% | 45,279,986 | 72.99% | 11.83% | 9,419,545 | 15.18% | 75,019,296 |
| gfp_48_2 | 65,451,761 | 60,356,560 | 92.22% | 53,126,437 | 81.17% | 7.78% | 7,230,123 | 11.05% | 74,599,751 |
| gfp_48_3 | 72,267,486 | 66,252,133 | 91.68% | 58,169,751 | 80.49% | 8.32% | 8,082,382 | 11.18% | 81,543,851 |
| gfp_72_1 | 73,097,534 | 68,327,976 | 93.48% | 60,585,755 | 82.88% | 6.52% | 7,742,221 | 10.59% | 83,247,054 |
| gfp_72_2 | 66,572,995 | 62,081,699 | 93.25% | 54,839,530 | 82.38% | 6.75% | 7,242,169 | 10.88% | 76,977,086 |
| gfp_72_3 | 66,903,351 | 61,813,748 | 92.39% | 54,577,976 | 81.58% | 7.61% | 7,235,772 | 10.82% | 76,944,856 |
| hbv_48_1 | 54,228,235 | 50,317,733 | 92.79% | 43,575,294 | 80.36% | 7.21% | 6,742,439 | 12.43% | 64,463,333 |
| hbv_48_2 | 66,242,876 | 61,179,224 | 92.36% | 53,795,112 | 81.21% | 7.64% | 7,384,112 | 11.15% | 75,013,066 |
| hbv_48_3 | 59,761,079 | 54,826,230 | 91.74% | 47,973,672 | 80.28% | 8.26% | 6,852,558 | 11.47% | 68,091,603 |
| hbv_72_1 | 68,226,176 | 62,726,736 | 91.94% | 55,527,851 | 81.39% | 8.06% | 7,198,885 | 10.55% | 76,985,743 |
| hbv_72_2 | 68,525,390 | 63,206,866 | 92.24% | 55,804,636 | 81.44% | 7.76% | 7,402,230 | 10.80% | 78,067,158 |
| hbv_72_3 | 69,793,432 | 64,649,015 | 92.63% | 56,680,020 | 81.21% | 7.37% | 7,968,995 | 11.42% | 82,054,442 |
