# Supplementary material for: Transcriptome-Wide Analysis of Hepatitis B Virus-Mediated Changes to Normal Hepatocyte Gene Expression
Source: PLoS Pathog. 2016 Feb 18;12(2):e1005438. doi: 10.1371/journal.ppat.1005438 (PMC4758756; doi:10.1371/journal.ppat.1005438)
Supplement: S4 Table — (DOCX) [file ppat.1005438.s010.docx]

S4 Table. qPCR primers for detection of differentially expressed transcripts

| Primer Name | 5' -> 3' Sequence |
| --- | --- |
| **Rat Primers** |  |
| Plek2 F | CAGCCCTGTACACTTTTGCTG |
| Plek2 R | ATCGGCGTACCTTCCAGTTC |
| Commd3 F | GAAATCCTACTGGGAAGTATAGGT |
| Commd3 R | GAGTTGCTCTTTCCAGGCTTT |
| Mat2a F | AGAGTCGCCTTCTCTCATCG |
| Mat2aR | AGCCACTTTAGCATCAGGGT |
| Cps1 F | GCTCCTAGCTTTGCAGTGGA |
| Cps1 R | TCCTTGTTGGGACAGATGCC |
| Lonp1 F | GGTGTTCCCGCGCTTTATCA |
| Lonp1 R | TGTGAATCCTTCTGTGGCCTGTA |
| Kdm1a F | CAAGCGGGCGAAGGTAGAAT |
| Kdm1a R | GATGCTTGGCCGTCTCCATA |
| Cdkn1a F | ACAGTGAGCAGTTGAGCCGCG |
| Cdkn1a R | TCCAGTGGCGTCTCAGTGG |
|  |  |
| **Human Primers** |  |
| Slco5a1 F | ACACAACGGGGAATCTGACA |
| Slco5a1 R | ACTTGTCGGCTTTGGACACA |
| Commd3 F_H | TCCTACTGGGAAGTATAGGCAGA |
| Commd3 R_H | TCCAGGCTTTTCGAAGCATCT |
| Mat2a F_H | CTTCCACGAGGCGTTCATC |
| Mat2a R_H | ACAGCTTCACGAACCACTTTC |
| Cps1_F_H | TGAAGATTTAGCCGAGGCCCA |
| Cps1_R_H | CACCAGCAACAGAGGATGGA |
| p21 F_H | GCCGAAGTCAGTTCCTTGTG |
| p21 R_H | TCGAAGTTCCATCGCTCACG |
| Slco5a1 F_H | ACACTCAGGTCCGTAGAAGATG |
| Slco5a1 R_H | GCATGCAGGTGGTGTCAATG |
